# Characterising variation in wheat traits under hostile soil conditions in India

**DOI:** 10.1371/journal.pone.0179208

**Published:** 2017-06-12

**Authors:** Jaswant S. Khokhar, Sindhu Sareen, Bhudeva S. Tyagi, Gyanendra Singh, Apurba K. Chowdhury, Tapamay Dhar, Vinod Singh, Ian P. King, Scott D. Young, Martin R. Broadley

**Affiliations:** 1 School of Biosciences, University of Nottingham, Sutton Bonington Campus, Loughborough, United Kingdom; 2 Indian Institute of Wheat & Barley Research, Aggarsain Marg, Karnal (Haryana), India; 3 Uttar Banga Krishi Viswavidyalaya, Pundibari, District Cooch Behar (West Bengal), India; 4 Narendra Deva University of Agriculture and Technology, Kumarganj, Faizabad (Uttar Pradesh), India; National Institute of Plant Genome Research, INDIA

## Abstract

Intensive crop breeding has increased wheat yields and production in India. Wheat improvement in India typically involves selecting yield and component traits under non-hostile soil conditions at regional scales. The aim of this study is to quantify G*E interactions on yield and component traits to further explore site-specific trait selection for hostile soils. Field experiments were conducted at six sites (pH range 4.5–9.5) in 2013–14 and 2014–15, in three agro-climatic regions of India. At each site, yield and component traits were measured on 36 genotypes, representing elite varieties from a wide genetic background developed for different regions. Mean grain yields ranged from 1.0 to 5.5 t ha^-1^ at hostile and non-hostile sites, respectively. Site (E) had the largest effect on yield and component traits, however, interactions between genotype and site (G*E) affected most traits to a greater extent than genotype alone. Within each agro-climatic region, yield and component traits correlated positively between hostile and non-hostile sites. However, some genotypes performed better under hostile soils, with site-specific relationships between yield and component traits, which supports the value of ongoing site-specific selection activities.

## Introduction

### Wheat breeding in India

Wheat is the world’s third most widely consumed crop after rice and maize. It accounts directly for ~21% of energy intake for the world’s population [1, 2] and more than 50% of the energy intake in the Indian population (www.dwr.in). In India, wheat production increased from 11 Mt to 94 Mt from 1961 to 2016 [3, 4] due to selective breeding for high yielding semi-dwarf varieties. Seeds of varieties including Sonara 63, Sonara 64, Mayo 64 and Lerma Rojo 64 along with 613 segregating lines were introduced in India from the International Maize and Wheat Improvement Center (CIMMYT) and led to the release of five commercial varieties (PV 18, Kalyan Sona, Sonalika, Choti Lerma and Safed Lerma) which led to the Green Revolution in India [5] (www.dwr.in). At present, wheat breeding programmes in India are co-ordinated by the IIWBR (Indian Institute of Wheat & Barley Research), formerly DWR (Directorate of Wheat Research), based at Karnal, through the All India Coordinated Wheat & Barley Improvement Programme (AICW&BIP). Since the establishment of AICW&BIP, there have been major developments in yield potentials, productivity and sustainability of wheat in India. For example, the area, productivity and production of wheat increased by 123%, 226%, and 630%, respectively, in 2006–07, compared to 1965–66 (www.dwr.in).

New wheat varieties are developed in India through a systematic testing procedure. The IIWBR coordinates multi-location testing of wheat and barley varieties and technologies at 31 centres located in State Agricultural Universities (SAUs) and other centres across six agro-climatic zones for wheat cultivation. These zones are: 1. Northern Hill Zone (NHZ), 2. North-Western Plains Zone (NWPZ), 3. North-Eastern Plains Zone (NEPZ), 4. Central Zone (CZ), 5. Peninsular Zone (PZ), 6. Southern Hills Zone (SHZ). Variety trials, grain multiplication and the field evaluation trials involves evaluation for yield and contributing traits, disease incidence, agronomic practices and grain quality traits. Each year, ~500 accessions/entries are tested in ~450 varietal trials at ~130 locations (www.dwr.in).

### Marginal soils are widespread in India and can contribute to sustainable intensification

There are several major abiotic constraints to further increases in wheat production in India, including widespread soil stresses. In India, 6.7 Mha land under wheat cultivation is affected by salt including 3 Mha by salinity and 3.7 Mha by sodicity/alkalinity, distributed across 15 of the 29 states. Out of these 15 states, eight contribute ~97% of national wheat production and have ~5.6 Mha affected by salt. Soil acidity affects 25 Mha of Indian soils, including ~30% of current areas under cultivation [6, 7]. The IIWBR has a Salinity/Alkalinity Screening Nursery (SATSN) programme which runs at a small number of locations each year. However, soils at most locations used for screening new varieties are classified as ‘normal’ within each cultivation zone and trials on marginal soils (e.g. saline, sodic, low pH) are not routinely conducted.

Saline soils are defined as having an electric conductivity (EC_e_) >4 dS m^-1^, an Exchangeable Sodium Percentage (ESP) <15, a Sodium Adsorption Ratio (SAR) <13 and a pH <8.2 [8, 9]. Saline soils are typically dominated by chloride and sulphate ions and are associated with arid and semiarid climates. Sodic soils have a higher pH (>8.2), ESP >15 and are also referred as alkaline soils [10]. Sodic soils are dominated by sodium ions, and carbonate and bicarbonate of sodium on exchange sites. These salts are responsible for the poor physical condition of the soil and give the soil black colour due to dispersion of organic matter and clay [11, 12]. Excessive salt in soil and water affects plants morphological, physiological and biochemical properties; resulting in reduction of crop growth and productivity [13]. Salinity may cause osmotic stress, ionic toxicity through osmotic and biochemical processes and nutritional imbalances through reduced uptake of essential elements [14–16]. Considerable fundamental knowledge has been gained in plant biology under salt stressed conditions at cellular and tissues level [15, 17, 18]. However, to our knowledge, new salt tolerant varieties have yet to be adopted following the targeting of specific physiological processes. Crops grown on acidic soils with pH <5.5 typically suffer from aluminum (Al) toxicities and deficiencies of phosphorus (P), boron (B) and molybdenum (Mo) [19].

Small-scale programs to screen for varieties within improved salt tolerance use a limited number of genotypes at a few sites in India. However, it is not yet clear which traits from main breeding programs under ‘normal’ soils will have value in breeding programs on hostile soils, which limit the deployment of trait-based selection. The aim of this study is therefore to quantify the contribution of environmental and genotypic sources of variation on grain yield and component traits in Indian wheat, using a common reference panel of diverse wheat genotypes. These data could help to identify which traits might be more amenable for crop improvement for marginal soils, based on site-specific selection.

## Materials and methods

### Experimental sites

The study was carried out at six field sites in major wheat growing areas of India, during *rabi* (winter) seasons in 2013/14 and 2014/15. Two sites were selected from within an agro-climatic region of the North Western Plains Zone (NWPZ); four sites were selected from the North Eastern Plains Zone (NEPZ), two sites from each of two different agro-climatic regions in NEPZ. Within each region, one site was selected to represent a ‘normal’ soil, the other a ‘hostile’ soil (Table 1). The NWPZ sites were, (1) IIWBR, Karnal, Haryana (HR); (2) IIWBR, Hisar (HR); NEPZ were, (3) Narendra Deva University of Agriculture and Technology (NDUA&T), Kumarganj, Faizabad, Uttar Pradesh (UP), reclaimed site; (4) NDUA&T, Kumarganj, Faizabad (UP), sodic site; (5) Uttar Banga Krishi Viswavidyalaya (UBKV), Pundibari, Cooch Behar, West Bengal (WB); (6) UBKV, Regional Research Sub-Station (RRSS), Mathurapur, Malda (WB). Sites (2), (4) and (5) represent ‘hostile’ soils (pH range 4.5–9.5), and sites (1), (3) and (6) represent ‘normal’ soils (pH range 7.2–8.3). Site 3 was reclaimed using heavy applications of Farm Yard Manure (FYM), >10 years ago. The experiments were conducted at research farms of IIWBR, Karnal; NDUA&T, Kumarganj and UBKV, Pundibari with their research director’s permission and study received the permission from Indian Council of Agricultural Research (ICAR) and Department of Biotechnology (DBT) under a collaborative project between India and University of Nottingham, UK, grant number BT/IN/UK/12/IS/2012.

**Table 1:** Sowing dates and cropping pattern of field experiments.

Site	Growing zone^a^	Grid	Sowing	Harvesting	Year	Soil type	Cropping pattern
**IIWBR, Karnal**	NWPZ	29.70° N	20.11.13	20.04.14	2013/14	Normal	Rice-Wheat
		76.99° E	26.11.14	23.04.15	2014/15		
**IIWBR, Hisar**	NWPZ	29.18° N	21.11.13	24.04.14	2013/14	Hostile	Cotton-Wheat
		75.70° E	19.11.14	22.04.15	2014/15		
**NDUA&T, Kumarganj-reclaimed**	NEPZ	26.43° N	28.11.13	26.04.14	2013/14	Normal	Rice-Wheat
		82.17° E	09.12.14	23.04.15	2014/15		
**NDUA&T, Kumarganj-sodic**	NEPZ	26.43° N	02.12.13	04.05.14	2013/14	Hostile	Rice-Wheat
		82.17° E	09.12.14	23.04.15	2014/15		
**RRSS, Mathurapur, Malda**	NEPZ	25.57° N	04.12.13	04.04.14	2013/14	Normal	Rice-Wheat
		87.10° E	25.11.14	08.04.15	2014/15		
**UBKV, Pundibari**	NEPZ	26.32° N	25.11.13	01.04.14	2013/14	Hostile	Rice-Wheat
		89.45° E	26.11.14	03.04.15	2014/15		

^a^NEPZ, North Eastern Plain Zone; NWPZ, North Western Plain Zone

### Germplasm

A panel of elite wheat genotypes comprising *Triticum aestivum* L. (n = 34) and *Triticum durum* L. (n = 2) were selected, represented a diverse genetic background and adaptations to a range of climatic and soil environments (Table 2).

**Table 2:** Wheat genotypes used in field trials during 2013/14 and 2014/15.

Variety	Pedigree	Date of release	Adoption Area^a^	Production Condition^b^
**Kharchia-65**	Kharchia Local/EG 953	1970	All zones	TS,IR
**HD-2009**	LR 64 A / NAI 60	1975	NWPZ	TS,IR
**UP-262**	S 308/BJ 66	1978	NEPZ	TS,IR
**KRL 1–4**	Kharchia 65/WL 711	1990	All zones	TS,IR
**HI-8498**	CR ‘S’-GS’S’//A-9-30-1/Raj 911	1999	CZ	TS,IR
**HW-2044**	HD 226*5/Sunstar*6/C-80-1	1999	SHZ	TS,IR
**KRL-19**	PBW 255/KRL 1–4	2000	All zones	TS,IR
**HD-2733**	Attila/3/Tui/Carc//Chen/Chto/4/Atila	2001	NEPZ	TS,IR
**GW-322**	PBW 173/GW 196	2002	CZ,PZ	TS,IR
**DBW-14**	Raj 3765/PBW 343	2002	NEPZ	LS,IR
**NW-1067**	TR 380-16-3-614/chat’S’	2004	UP	TS,IR
**DBW-16**	Raj 3765/WR 484//HUW 468	2006	NEPZ	TS,IR
**K-0307**	K 8321/UP 2003	2007	NEPZ	TS,IR
**DBW-17**	Cmh79a.95/3*Cno79//Raj 3777	2007	NWPZ	TS, IR
**WH-1021**	Nyot 95/Sonak	2007	NWPZ	LS,IR
**HD-2932**	Kauz/Star//HD 2643	2008	CZ	TS,IR
**CBW-38**	Cndo/r143//Ente/Mexi-2/3/Ae.squarrosa(taus)/4/Weaver/5/2*Pastor	2008	NEPZ	TS,IR
**DBW-39**	Attila/Hui	2010	NEPZ	TS,IR
**DBW-51**	Site/Milan	2010	NEPZ	LS,IR
**KRL-213**	Cndo/r143//Ente/Mexi-2/3Aegilops squarrosa(taus)/4/Weaver/5/2*Kauz	2010	NWPZ	TS,IR
**PDW-314**	Ajaia12/F3Local(SEL.Ethio.135.85)//Platai 13/3/Somat3/4/Sooty/Rascon37	2010	NWPZ	TS,IR
**KRL-210**	PBW 65/2*Pastor	2010	NWPZ/NEPZ	TS,IR
**MACS-6222**	HD 2189*2//MACS 2496	2010	PZ	TS,IR
**HI-1563**	MACS 2496*2MC 10	2011	NEPZ	LS,IR
**HD-2967**	Ald/Coc//User/HD 2160m/HD22778	2011	NWPZ	TS,IR
**DPW-621-50**	Kauz//Altar84/aOS/3/Milan/Kauz/4/Huites	2011	NWPZ	TS,IR
**RAJ-4238**	HW 2021/Raj 3765	2012	CZ	LS,IR
**RAJ-4229**	HW 2048/Raj 4000	2012	NEPZ	TS,IR
**NW-4018**	-	2013	NWPZ	LS,IR
**WH-1105**	Milan/S87230//Babax	2013	NWPZ	TS,IR
**DBW-71**	Prinia/UP 2425	2013	NWPZ	LS,IR
**RW-3684**	Pastor.florkwa.1//Pastor	Stock	-	TS,RF
**NW-4092**	Site/MO/Milan/3/PBW 343	Stock	NWPZ	LS,IR
**KRL 3–4**	HD 1982/Kharchia 65	^c^Stock (2009)	All zones	TS,IR
**BH-1146**	Eonta ponta grossa 1//Fretes/Martin	Stock (1987)	-	-
**DBW-46**	PBW 343/Inq21	Stock (2011)	-	-

^a^NEPZ, North Eastern Plain Zone; NWPZ, North Western Plain Zone; CZ, Central Zone; PZ, Peninsular Zone; SHZ, Southern Hills Zone

^b^TS, Timely Sown; LS, Late Sown; IR, Irrigated; RF, Rain fed

^c^Stock refers to advanced breeding line used in breeding programme

### Field management

Wheat was sown in *rabi* (winter) season in November/December and harvested during April/May in 2013/14 and 2014/15, at all the sites (Table 1). After conventional operations, including field preparation, fertilizing, disking, levelling and furrowing, seeds were manually placed in four rows per plot at 25 cm spacing between rows. The plot length was 2.5 m. The number of seeds sown for each genotype was the same for each plot (~624 seed). The plots were arranged in a simple lattice design (6x6) with two replicates according to standard IIWBR practices.

Fertilizers were applied at a rate of 150 kg N, 60 kg P_2_O_5_, and 40 kg K_2_O ha^-1^ as urea, triple super phosphate (TSP) and muriate of potash (MOP), respectively. All fertilizers, including one third of the N, were applied uniformly in the field during final land preparation. One third of the N was top dressed at nodal root initiation (NRI) stage, ~21 days after sowing (DAS), and the remaining N was applied at the time of second irrigation, ~45 DAS. Experiments were irrigated when required to avoid water stress to the plants, typically on three occasions, to bring the soil moisture close to the field capacity during NRI, booting and grain filling stages. Weeds were controlled manually at 30 DAS by hand weeding and then crops were protected from pests and disease as required. Crops were harvested at maturity, sun-dried and then threshed.

### Traits scored

Pre-harvest traits, for example, Grain number per spike (GNS); Grain weight per spike (GWS, g) and Plant height at maturity (PHT, cm) were recorded on five randomly selected plants per plot, which were tagged for subsequent measurements at maturity, alongside plot-scale measurements. Data were recorded on the following traits: Grain yield per plot at 12–13% dry weight (GYD, g); GNS; GWS; 1000 grain weight (TGW, g); Productive tillers per meter (PTM); Biological yield per plot (BYD, g); Harvest index (HI, %); Days to 75% heading (DTH, d); Days to anthesis (DTA, d); Days to 75% maturity (DTM, d); PHT.

### Statistical analysis

Variance components were calculated for all yield and component traits using Residual Maximum Likelihood (REML) analyses. Two-way analysis of variance (ANOVA) was used to test for differences in yield and yield component trait due to genotype and site factors, for each year, within each of the three agro-climatic regions. Means of yield and yield component traits were compared using Least Significant Difference (LSD). Differences between sites and genotypes for grain yield were considered significant at P<0.01. Pearson correlation coefficients were calculated to explore correlations among yield and component traits, for each site in each year. A principal component analysis (PCA) on data variates was performed to construct a biplot for grain yield to study the effect of genotype, and genotype by environment interaction, to explore the adaptation of genotypes to the specific sites. All data analyses were conducted using GenStat 17^th^ Edition (VSN International Ltd, Hemel Hempstead, UK).

## Results

### Contribution of G, E and G*E interaction to yield and components traits

Primary data are provide in S2 Table. Site accounted for most of the variation in yield and component traits in both years. For example, 76% and 70% of the variation in GYD was due to site factor in 2013/14 and 2014/15, respectively. Site also accounted for most of the variation in DTA (81 and 91%) and DTM (90 and 92%) in 2013/14 and 2014/15, respectively. In 2013/14, PHT was controlled by E (76%) factor to a larger extent than G (16%); in 2014/15 both G and G*E interaction affect PHT similarly (40 and 49% respectively). G and G*E interaction factors had a much greater influence on TGW, GWS, HI and PHT than on GYD, DTA and DTM during both the years.

### Variation in yield and component traits under normal and hostile soils

#### Trait comparisons between Karnal (normal) and Hisar (saline) sites

**Fig 1:**
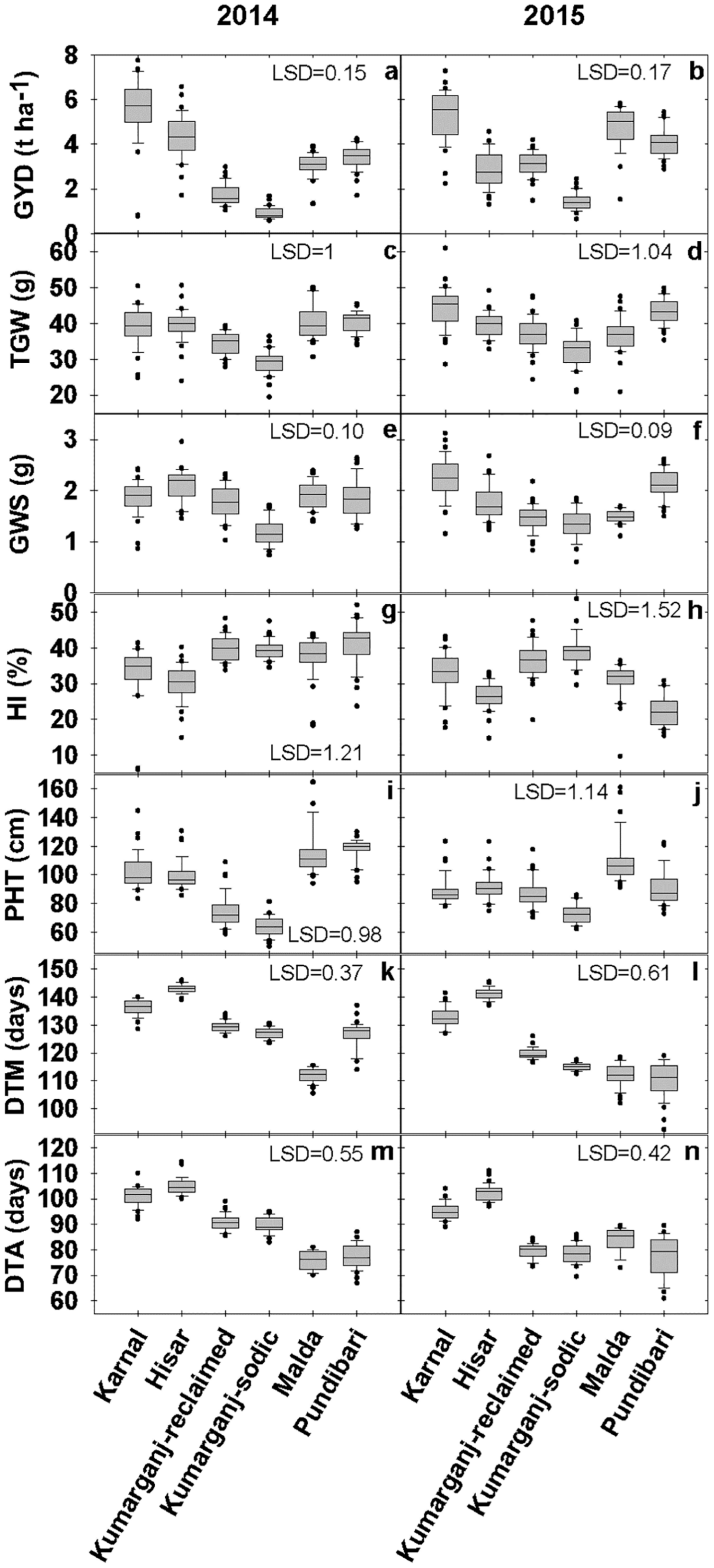
Yield and component traits at the six sites during 2013/14 and 2014/15. Data represent two replicate plots per site of a panel of 36 elite genotypes (n = 72). Boxes represent the two quartiles with the median drawn; whiskers are the 95% confidence limits plus outliers. Traits: GYD (grain yield, t ha^-1^), TGW (1000 grain weight, g), GWS (grain weight per spike, g), HI (harvest index, %), PHT (plant height at maturity, cm), DTM (days to maturity, days), DTA (days to anthesis, days).

Yield and component traits were typically greater at Karnal than Hisar. The GYD was greater at Karnal than at Hisar in both years (Fig 1a and 1b). In 2013/14, GYD was 5.5 and 4.4 t ha^-1^ at Karnal and Hisar, respectively (LSD = 0.44). In 2014/15, GYD was 5.3 and 2.9 t ha^-1^ at Karnal and Hisar, respectively (LSD = 0.34). The marginal and substantial yield reductions at Karnal and Hisar, respectively, in 2014/15 were due to heavy later rains (post-heading) (S1 Table). The TGW did not differ significantly between Karnal and Hisar in 2013/14 but was greater at Karnal in 2014/15 (Fig 1c and 1d). In 2013/14, TGW was 39.4 and 39.6 g at Karnal and Hisar, respectively (LSD = 1.87). In 2014/15, TGW was 44.5 and 39.8 at Karnal and Hisar, respectively (LSD = 1.76). Conversely, the GWS was slightly lower at Karnal than Hisar in 2013/14 (Fig 1e and 1f). In 2013/14, GWS was 1.9 and 2.1 g at Karnal and Hisar, respectively (LSD = 0.12). In 2014/15, GWS was 2.2 and 1.8 g at Karnal and Hisar, respectively (LSD = 0.15).

The HI was greater at Karnal than at Hisar in both years (Fig 1g and 1h). In 2013/14, HI was 33 and 30.5% at Karnal and Hisar, respectively (LSD = 2.32). In 2014/15, HI was 33.2 and 26.5% at Karnal and Hisar, respectively (LSD = 1.74). The PHT did not differ significantly between Karnal and Hisar in either year (Fig 1i and 1j). In 2013/14, PHT was 101.9 and 99.2 cm at Karnal and Hisar, respectively (LSD = 3.76). In 2014/15, PHT was 88.3 and 91.4 cm at Karnal and Hisar respectively (LSD = 3.06).

Crop development was slightly faster at Karnal than Hisar. Thus, the phenological stages DTM and DTA were earlier at Karnal than at Hisar in both years (Fig 1k–1n). In 2013/14, DTM was 136 and 143 days at Karnal and Hisar, respectively (LSD = 0.81). In 2014/15, DTM was 133 and 141 days at Karnal and Hisar, respectively (LSD = 1.03). In 2013/14, DTA was 101 and 105 days at Karnal and Hisar, respectively (LSD = 1.18). In 2014/15, DTA was 95 and 103 days at Karnal and Hisar, respectively (LSD = 1.07).

Two-way analysis of variance (ANOVA) was used to assess the variation in traits due to genotype and site factors across both years, showed that most of the yield and component traits differed significantly between sites, except PHT (*P* = 0.8) 8(S3 Table).

#### Trait comparisons between Kumarganj-reclaimed (normal/saline) and Kumarganj-sodic (sodic) sites

Yield and component traits were greater at the Kumarganj-reclaimed than the Kumarganj-sodic sites. The GYD was greater at the reclaimed than at the sodic site in both years. In 2013/14, GYD was 1.8 and 0.9 t ha^-1^ at reclaimed and sodic sites, respectively (LSD = 0.13). In 2014/15, GYD was 3.1 and 1.4 t ha^-1^ at reclaimed and sodic sites, respectively (LSD = 0.19) (Fig 1a and 1b). The TGW was greater at the reclaimed site than at the sodic site in both years. In 2013/14, TGW was 34.5 and 29.1 g at reclaimed and sodic sites, respectively (LSD = 1.15). In 2014/15, TGW was 37 and 32.3 g at reclaimed and sodic sites, respectively (LSD = 1.65) (Fig 1c and 1d). However, the GWS was greater at the reclaimed site than at the sodic site in both years. In 2013/14, the GWS was 1.8 and 1.2 g at reclaimed and sodic sites, respectively (LSD = 0.11). In 2014/15, GWS was 1.5 and 1.4 g at reclaimed and sodic sites, respectively (LSD = 0.09) (Fig 1e and 1f).

The HI did not differ significantly between reclaimed and sodic sites in 2013/14, but was greater at the sodic site in 2014/15. In 2013/14, HI was 39.9 and 39.3% at reclaimed and sodic sites, respectively (LSD = 1.15). In 2014/15, HI was 36.5 and 39.5% at reclaimed and sodic sites, respectively (LSD = 2.25) (Fig 1g and 1h). The PHT was higher at the reclaimed site than at the sodic site in both years. In 2013/14, PHT was 74.4 and 63.8 cm at reclaimed and sodic sites, respectively (LSD = 3.1). In 2014/15, PHT was 86.7 and 72.8 cm at reclaimed and sodic sites, respectively (LSD = 2.87) (Fig 1i and 1j).

Crop development was slightly slower at the reclaimed site than at the sodic site. Thus, DTM was greater at the reclaimed site than at the sodic site in both years. In 2013/14, DTM was 130 and 127 days at reclaimed and sodic sites, respectively (LSD = 0.67). In 2014/15, DTM was 120 and 115 days at reclaimed and sodic sites, respectively (LSD = 0.58) (Fig 1k and 1l). However, The DTA did not differ significantly between reclaimed and sodic sites in either year. In 2013/14, DTA was 91 and 90 days at reclaimed and sodic sites, respectively (LSD = 1.08). In 2014/15, DTA was 79 days at both reclaimed and sodic sites (LSD = 1.11) (Fig 1m and 1n).

Two-way ANOVA showed that all of the yield and component traits differed significantly between the Kumarganj-reclaimed and Kumarganj-sodic sites, except for HI (*P* = 0.06) and DTA (*P* = 0.24) (S3 Table).

#### Trait comparisons between Malda (normal) and Pundibari (acidic) sites

The GYD was lower at Malda than Pundibari in 2013/14 while in 2014/15 Malda had greater GYD than Pundibari (Fig 1a and 1b). In 2013/14, GYD was 3.1 and 3.4 t ha^-1^ at Malda and Pundibari, respectively (LSD = 0.21). In 2014/15, GYD was 4.8 and 4.1 t ha^-1^ at Malda and Pundibari, respectively (LSD = 0.29). The TGW and GWS did not differ significantly between Malda and Pundibari sites in 2013/14 but were greater at Pundibari than at Malda in 2014/15. In 2013/14, TGW was 40.4 and 40.5 g at Malda and Pundibari, respectively (LSD = 1.52). In 2014/15, TGW was 36.7 and 43.4 g at Malda and Pundibari, respectively (LSD = 1.58) (Fig 1c and 1d). In 2013/14, GWS was 1.9 and 1.8 g at Malda and Pundibari, respectively (LSD = 0.14). In 2014/15, GWS was 1.5 and 2.1 g at Malda and Pundibari, respectively (LSD = 0.09) (Fig 1e and 1f).

The HI did not show consistent differences between Malda and Pundibari sites between years. In 2013/14, HI was 37.5 and 41.3% at Malda and Pundibari, respectively (LSD = 2.13). In 2014/15, HI was 30.8 and 22.3% at Malda and Pundibari, respectively (LSD = 1.73) (Fig 1g and 1h). The PHT did not differ significantly between Malda and Pundibari sites in 2013/14 but was greater at Malda than at Pundibari in 2014/15. In 2013/14, PHT was 115.2 and 117.6 cm at Malda and Pundibari, respectively (LSD = 4.23). In 2014/15, PHT was 109.6 and 91.2 cm at Malda and Pundibari respectively (LSD = 4.82) (Fig 1i and 1j).

Crop development did not show overall consistent differences between sites, between each year. The DTM differed significantly between Malda and Pundibari in 2013/14 but not in 2014/15. In 2013/14, DTM was 112 and 127 days at Malda and Pundibari, respectively (LSD = 1.23). In 2014/15, DTM was 112 and 111 days at Malda and Pundibari, respectively (LSD = 1.79) (Fig 1k and 1l).In 2013/14, DTA was 76 and 77 days at Malda and Pundibari, respectively (LSD = 1.46). In 2014/15, DTA was 84 and 78 days at Malda and Pundibari, respectively (LSD = 2.12) (Fig 1m and 1n).

### Variation in grain yield in panels of 36 genotypes under normal and hostile soils

**Fig 2:**
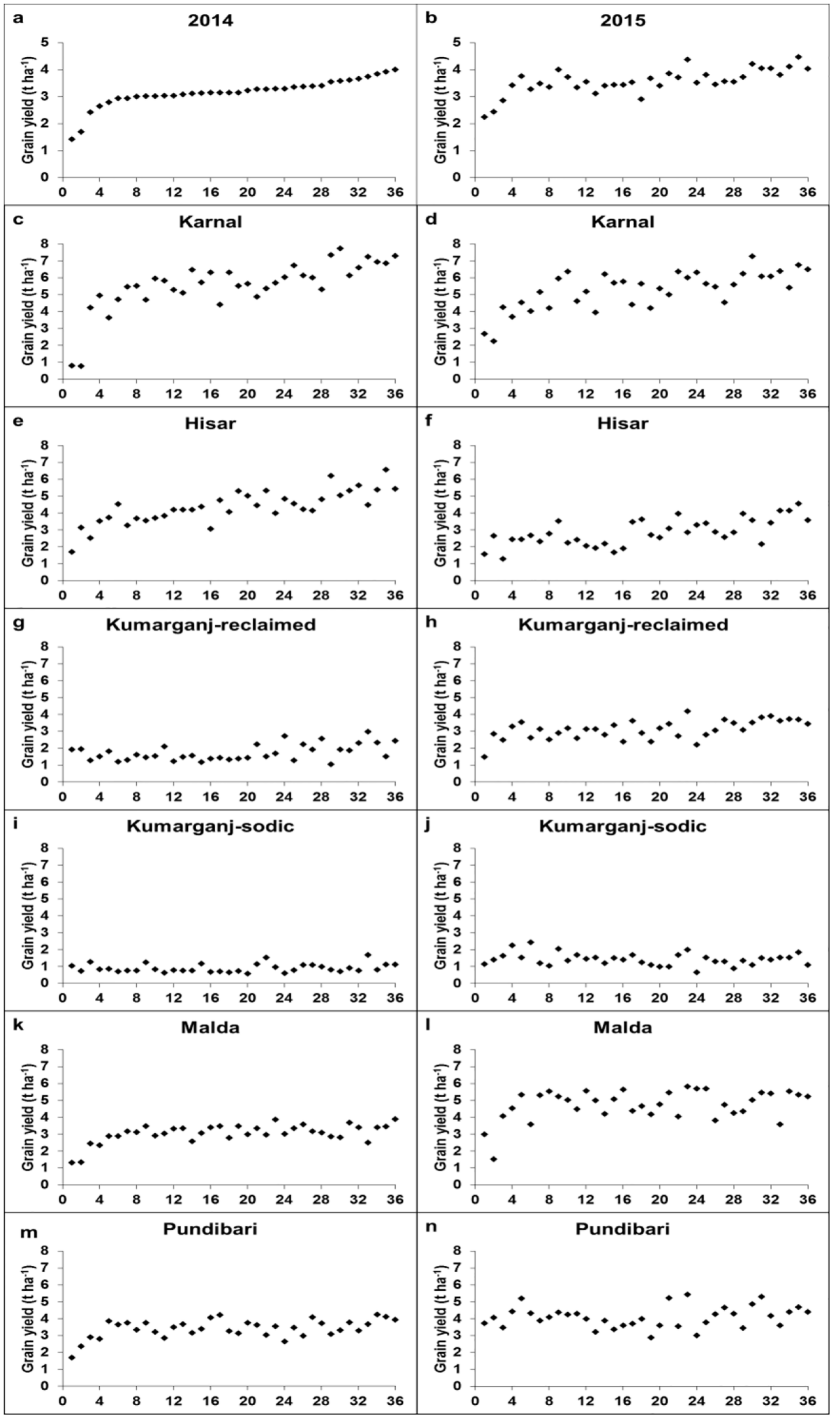
Mean grain yield of wheat harvested in 2013/14 (a,c,e,g,i,k,m) and 2014/15 (b,d,f,h,j,l,n) at six sites. In panels (a) and (b), data are means of each genotype at all sites. For all other panels, data are means of two replicate plots per site each year. Genotypes 1–36 are labelled in the same order on the x-axis as: (1) Kharchia-65; (2) KRL 3–4; (3) KRL 19; (4) BH 1146; (5) UP 262; (6) Raj 4238; (7) DBW 14; (8) Raj 4229; (9) HD 2932; (10) NW 4092; (11) HD 2009; (12) GW 322; (13) HI 1563; (14) NW 4018; (15) HW 2044; (16) KRL 1–4; (17) HD 2733; (18) HI 8498; (19) DBW 51; (20) MACS 6222; (21) DBW 16; (22) NW 1067; (23) KRL 213; (24) PDW 314; (25) WH 1021; (26) DBW 46; (27) K 0307; (28) DBW 17; (29) WH 1105; (30) RW 3684; (31) DBW 71; (32) CBW 38; (33) DPW 621–50; (34) HD 2967; (35) KRL 210; (36) DBW 39.

In general, there were positive relationships in grain yield between years across all sites within an agro-climatic region (Fig 2). However, there is the potential for some genotypes to perform better at specific sites, i.e. the highest and lowest yielding genotypes were not usually consistent between sites.

#### Yield comparison of different genotypes between Karnal (normal) and Hisar (saline) sites over two seasons

There was a strong positive relationship in GYD between Karnal and Hisar in both 2013/14 (r = 0.65; P<0.001) and 2014/15 (r = 0.51; P<0.01). At Karnal, the highest yielding genotype was RW 3684, which had the highest GYD in 2013/14 (7.8 t ha^-1^) and 2014/15 (7.3 t ha^-1^) (Fig 2c and 2d). At Hisar, RW 3684 was the 9^th^ highest GYD producing genotype in 2013/14 (5.1 t ha^-1^) and 8^th^ in 2014/15 (3.6 t ha^-1^) (Fig 2e and 2f). The lowest yielding genotype at Karnal was KRL 3–4 in 2013/14 (0.8 t ha^-1^) and 2014/15 (2.2 t ha^-1^). At Hisar, KRL 3–4 was the 4^th^ and 16^th^ lowest yielding genotype in 2013/14 (3.1 t ha^-1^) and 2014/15 (2.7 t ha^-1^), respectively.

At Hisar, the highest yielding genotype was KRL 210, which had the highest GYD in 2013/14 (6.6 t ha^-1^) and 2014/15 (4.6 t ha^-1^). At Karnal, KRL 210 was the 6^th^ and 2^nd^ highest GYD producing genotype in 2013/14 (6.9 t ha^-1^) and 2014/15 (6.8 t ha^-1^), respectively. The lowest yielding genotype at Hisar was Kharchia 65 in 2013/14 (1.7 t ha^-1^) and KRL 19 in 2014/15 (1.3 t ha^-1^). At Karnal, Kharchia 65 and KRL 19 were the 2^nd^ and 8^th^ lowest yielding genotypes in 2013/14 (0.8 t ha^-1^) and 2014/15 (4.3 t ha^-1^), respectively.

#### Yield comparisons between Kumarganj-reclaimed (normal/saline) and Kumarganj-sodic (sodic) sites over two seasons

There was a weak and non-significant relationship in GYD between reclaimed and sodic sites in both 2013/14 (r = 0.25; P = 0.141) and 2014/15 (r = 0.19; P = 0.267). At the reclaimed site, the highest yielding genotypes were DPW 621–50 and KRL 213, which had the highest GYD in 2013/14 (2.9 t ha^-1^) and 2014/15 (4.2 t ha^-1^), respectively (Fig 2g and 2h). At the sodic site, DPW 621–50 and KRL 213 were the 1^st^ and 4^th^ highest GYD producing genotypes in 2013/14 (1.7 t ha^-1^) and 2014/15 (2 t ha^-1^), respectively (Fig 2i and 2j). The lowest yielding genotype at the reclaimed site was WH 1105 in 2013/14 (1 t ha^-1^) and Kharchia 65 in 2014/15 (1.5 t ha^-1^). At the sodic site, WH 1105 and Kharchia 65 were the 18^th^ and 9th^th^ lowest yielding genotypes in 2013/14 (0.8 t ha^-1^) and 2014/15 (1.2 t ha^-1^), respectively.

At the sodic site, the highest yielding genotypes were DPW 621–50 and Raj 4238, which had the highest GYD in 2013/14 (1.7 t ha^-1^) and 2014/15 (2.5 t ha^-1^), respectively. At the reclaimed site, DPW 621–50 and Raj 4238 were the 1^st^ and 29^th^ highest GYD producing genotypes in 2013/14 (2.9 t ha^-1^) and 2014/15 (2.6 t ha^-1^), respectively. The lowest yielding genotype at the sodic site was MACS 6222 in 2013/14 (0.6 t ha^-1^) and PDW 314 in 2014/15 (0.7 t ha^-1^). At the reclaimed site, MACS 6222 and PDW 314 were the 12^th^ and 2^nd^ lowest yielding genotypes in 2013/14 (1.4 t ha^-1^) and 2014/15 (2.2 t ha^-1^), respectively.

#### Yield comparisons between Malda (normal) and Pundibari (acidic) sites over two seasons

There was a strong positive relationship in GYD between Malda and Pundibari sites in 2013/14 (r = 0.71; P<0.001) and a weak and non-significant relationship in 2014/15 (r = 0.256; P = 0.132).

At Malda, the highest yielding genotypes were DBW 39 and KRL 213, which had the highest GYD in 2013/14 (3.9 t ha^-1^) and 2014/15 (5.8 t ha^-1^) respectively (Fig 2k and 2l). At Pundibari, DBW 39 and KRL 213 were the 6^th^ and 1^st^ highest GYD producing genotypes in 2013/14 (3.9 t ha^-1^) and 2014/15 (5.4 t ha^-1^), respectively (Fig 2m and 2n). The lowest yielding genotype at Malda was Kharchia 65 in 2013/14 (1.1 t ha^-1^) and KRL 3–4 in 2014/15 (1.5 t ha^-1^). At Pundibari, Kharchia 65 and KRL 3–4 were the 1^st^ and 18^th^ lowest yielding genotypes in 2013/14 (1.7 t ha^-1^) and 2014/15 (4.1 t ha^-1^), respectively.

At Pundibari, the highest yielding genotypes were HD 2967 and KRL 213, which had the highest GYD in 2013/14 (4.3 t ha^-1^) and 2014/15 (5.4 t ha^-1^), respectively. At Malda, HD 2967 and KRL 213 were the 9^th^ and 1^st^ highest GYD producing genotypes in 2013/14 (3.4 t ha^-1^) and 2014/15 (5.8 t ha^-1^), respectively. The lowest yielding genotype at Pundibari was Kharchia 65 in 2013/14 (1.7 t ha^-1^) and HI 8498 in 2014/15 (2.9 t ha^-1^). At Malda, Kharchia 65 and HI 8498 were the 1^st^ and 8^th^ lowest yielding genotypes in 2013/14 (1.3 t ha^-1^) and 2014/15 (4.2 t ha^-1^), respectively.

### Biplot analysis for grain yield

**Fig 3:**
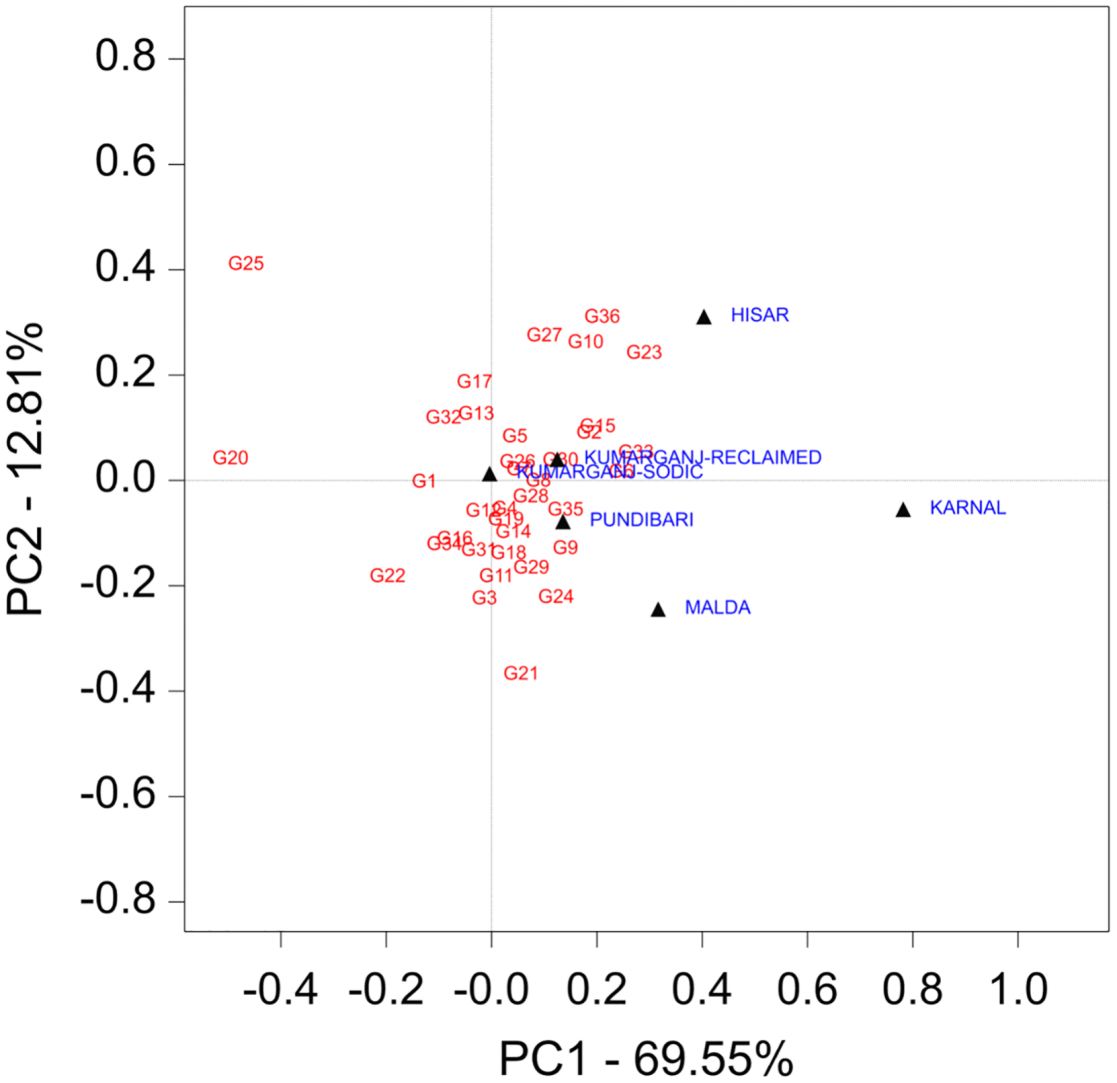
Biplot for grain yield of 36 genotypes evaluated at 6 sites over two years. Genotypes G1-G36 are as: G1 (BH 1146); G2 (CBW 28); G3 (DBW 14); G4 (DBW 16); G5 (DBW 17); G6 (DBW 39); G7 (DBW 46); G8 (DBW 51); G9 (DBW 71); G10 (DPW 621–50); G11 (GW 322); G12 (HD 2009); G13 (HD 2733); G14 (HD 2932); G15 (HD 2967); G16 (HI 1563); G17 (HI 8498); G18 (HW 2044); G19 (K 0307); G20 (Kharchia 65); G21 (KRL 1–4); G22 (KRL 19); G23 (KRL 210); G24 (KRL 213); G25 (KRL 3–4); G26 (MACS 6222); G27 (NW 1067); G28 (NW 4018); G29 (NW 4092); G30 (PDW 314); G31 (Raj 4229); G32 (Raj 4238); G33 (RW 3684); G34 (UP 262); G35 (WH 1021); G36 (WH 1105).

The genotype and genotype by environment effects were visualised using a biplot (Fig 3). The biplot explained 82.4% of total variation with the contribution of 69.6% from principal component-1 (PC-1) and 12.8% from principal component 1 (PC-2). The genotypes G4 (DBW 16), G5 (DBW 17), G7 (DBW 46), G8 (DBW 51), G26 (MACS 6222) and G15 (HD 2967) which have the PC1 value >0 and PC2 value near to zero being considered the more stable genotypes across all the sites (Fig 3). Genotypes which are closest to specific sites indicates that they are likely to be better suited to that particular site (for grain yield). For example; G23 (KRL 210), G36 (WH 1105) and G10 (DPW 621–50) showed higher grain yield at Hisar site. Similarly, G21 (KRL 1–4), G24 (KRL 213), G3 (DBW 14), G11 (GW 322) and G29 (NW 4092) are better suited for Malda site in terms of grain yield.

### Correlations among yield and contributing traits

**Fig 4:**
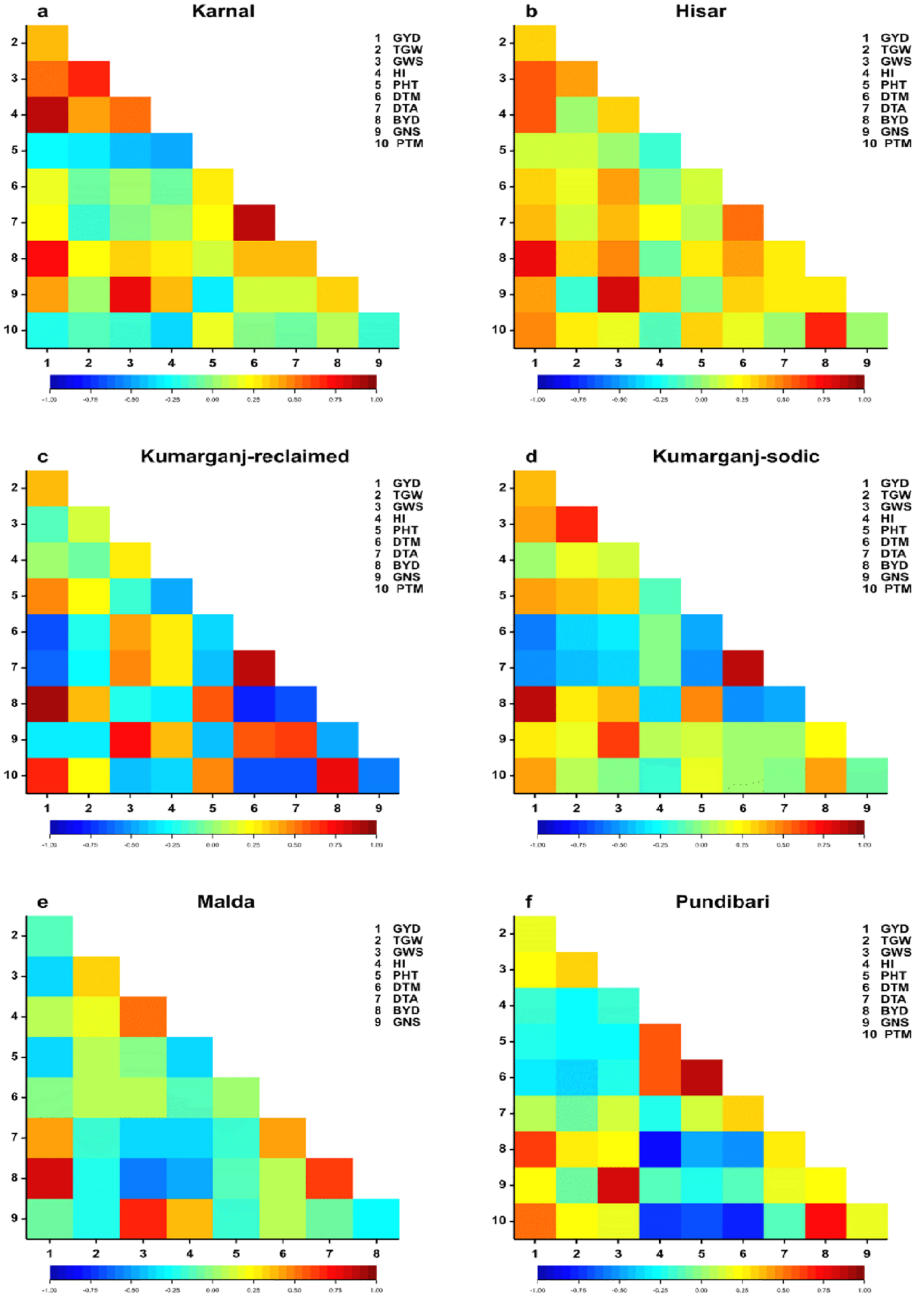
Correlation coefficients among yield and component traits of a panel of elite 36 wheat genotypes grown at six sites in India in 2013/14 and 2014/15. Colour represents strength of correlation from strongly negative (dark blue) to strongly positive (dark red). Traits measured 1–10 are labelled as: (1) grain yield (GYD); (2) 1000 grain weight (TGW); (3) grain weight per spike (GWS); (4) harvest index (HI); (5) Plant height at maturity (PHT); (6) Days to maturity (DTM); (7) Days to anthesis (DTA); (8) biological yield (BYD); (9) grain number per spike (GNS); (10) Productive tillers per meter (PTM).

There were significant positive correlations between GYD and BYD at all the sites. There were also positive significant correlations between GYD and TGW at all the sites except Malda and Pundibari (Fig 4). Among other quantitative traits, there was a positive significant correlation between TGW and GWS, between GNS and GWS, and between DTM and DTA at all sites. There was a positive significant correlation between GNS and HI at all the sites except for Kumarganj-sodic and Pundibari sites.

#### Correlations among yield and component traits at Karnal (normal) and Hisar (saline) sites

At Karnal, there was a significant positive correlation between GYD and TGW, GWS, HI, BYD and GNS, but GYD did not correlate with PHT, DTM, DTA and PTM (Fig 4a). At Hisar, there was a significant positive correlation between GYD and all of the traits except PHT (Fig 4b).

#### Correlations among yield and component traits at Kumarganj-reclaimed (normal/saline) and Kumarganj-sodic (sodic) sites

At the reclaimed site, there was a significant positive correlation between GYD and TGW, PHT, BYD and PTM, and a significant negative correlation between GYD and DTM, DTA and GNS (Fig 4c). At the sodic site, there was a significant positive correlation between GYD and TGW, GWS, PHT, BYD, GNS and PTM, and a significant negative correlation between GYD and DTM and DTA (Fig 4d). GYD did not correlate with HI at either site.

#### Correlations among yield and component traits at Malda (normal) and Pundibari (acidic) sites

At Malda, there was a significant positive correlation between GYD and DTA and BYD, and a significant negative correlation between GYD and GWS and PHT (Fig 4e). However, GYD did not correlate with TGW, HI, DTM or GNS. At Pundibari, there was a significant positive correlation between GYD and BYD and PTM, and a significant negative significant correlation between GYD and DTM (Fig 4f). However, GYD did not correlate with TGW, GWS, HI, PHT, DTA or GNS.

## Discussion

Grain yield is typically the most important trait in wheat breeding programmes. It is a result of interaction of many contributing traits which affect the grain yield directly and indirectly. Although site-specific selection has long-been adopted in cereal breeding, it remains important to quantify and understand relationships between grain yield and contributing traits under as many environments as possible. The contribution of genotypic (G) and environmental (site and year, E) sources of variation on grain yield and component traits in Indian wheat was determined to identify which traits might be most useful for site-specific selection on hostile soils. Although E had the largest effect on yield and component traits, yield and component traits correlated positively between hostile and non-hostile sites within each agro-climatic region, reflecting a large contributory effect of G. However, for most traits, more of the variation was associated with interactions between genotype and site terms (G*E) than it was to G alone. Thus, some genotypes performed better under hostile soils and site-specific correlations between yield and component traits were observed.

### Effects of site on trait variation

Given the diverse selection of hostile and non-hostile soils from within three agro-climatic regions, it is unsurprising that site accounted most of the variation in all the traits. The hostile and non-hostile soils differed considerably in their characteristics. For example, Karnal soils were pH 7.5, with an EC of 0.2, dS m^-1^, and organic carbon (OC) content of 0.45%. In contrast, Hisar soils were pH 8.1, with an EC of 0.41 dS m^-1^ and OC of 0.58%. Grain yield averaged across all genotypes and year ranged from 5.4 to 3.6 t ha^-1^ at Karnal and Hisar, respectively. Kumarganj-reclaimed site soils were pH 8.2, with an EC of 0.2, dS m^-1^, and OC content of 0.43%. On contrast, Kumarganj-sodic site soils were pH 9.4, with an EC of 0.7, dS m^-1^ and OC of 0.34%. Grain yield averaged across all genotypes and year ranged from 2.4 to 1.1 t ha^-1^ at reclaimed and sodic sites, respectively. Reduction in GYD in wheat under hostile soils (saline/sodic) is consistent with studies of Munns and Tester [15], James *et al*. [20], Ashraf *et al*. [14], Lenis *et al*. [16], Zhang *et al*. [21], and Wright and Rajper [22] who reported yield reduction in wheat under sodic soils were due to fewer grains per plant and reduced grain weight.

Malda soils were pH 7.7, with an EC of 0.2, dS m^-1^, and OC content of 0.72%. Conversely, Pundibari soils were pH 5.7, with an EC of 0.2, dS m^-1^, and OC of 0.74%. Grain yield averaged across all genotypes and year ranged from 3.9 to 3.7 t ha^-1^ at Malda and Pundibari, respectively. Reduction in GYD under acidic soils is consistent with Tang *et al*. [23], Tang *et al*. [24], Haling *et al*. [25], and Bian *et al*. [26].

Soil management has been adopted to increase wheat production under salt affected soils. For example, soil management by using gypsum (calcium sulphate, CaSO_4_), adequate drainage, land levelling, farm yard manure (FYM) and green manure [27]. Installing PVC pipes below the soil surface is also used to provide subsurface drainage to promote leaching. Approximately 50,000 ha of waterlogged saline soils have been reclaimed in India using these technologies [28]. Liming is the most economical and widely used method to reclaim acidic soils in India. Typically, ground limestone from calcite (CaCO_3_), dolomite (CaCO_3_, MgCO_3_) is used to neutralize acidic soils. Generally, lime is broadcast on the soil surface and mixed with soil during tillage [29]. Soil liming can increase the crop-availability of phosphorus (P) and other nutrients in the soil [30]. However, chemical and mechanical management of hostile soils increase input costs and, where farmers have small land holdings is likely to require subsidies. There is therefore a need to develop varieties tolerant to hostile soils [19].

### Effects of genotype on trait variation

The elite panel of 36 genotypes were selected to encompass a wide genetic diversity with extreme morphologies, including wide variations in height, maturity date, and yield. For all yield and component traits, the variation accounted for by G was less than the variation accounted for by E. Similarly, the variation accounted for by G*E interactions accounted for more variation than G alone for most yield and component traits, with the exceptions of PHT and DTA.

Genotypes showed a strong positive correlation in GYD between Karnal and Hisar sites, and between Malda and Pundibari sites. However, there was a weak and non-significant correlation in GYD between Kumarganj-reclaimed and Kumarganj-sodic sites. Thus, whilst genotypes did not perform identically on hostile and non-hostile soils, there are common traits which can be selected for hostile conditions under standard screening. Within each agro-climatic region, many yield component traits showed positive correlations between hostile and non-hostile soils. However, TGW, GWS and HI showed only weak and non-significant correlations between Kumarganj-reclaimed and Kumarganj-sodic sites, indicating that genotypes did not perform consistently between these sites and that there is scope for site-specific selection of traits.

### Effects of G*E on trait variation

The G*E interaction term was associated with more of the variation in TGW, GWS, HI and GYD than PHT, DTA and DTM. Therefore, there is potentially more scope for site-specific selection for these traits. In addition, within each agro-climatic region, correlations between yield and component traits differed between hostile and non-hostile soils.

#### Karnal (normal) and Hisar (saline) sites

There was a positive correlation between GYD and DTA, DTM and PTM at Hisar, but not at Karnal; Kharchia 65 and KRL 3–4 had greater GYD at Hisar than Karnal. Similarly, there was a significant positive correlation between TGW and BYD and PTM at Hisar but not at Karnal. Genotypes HI 1563, MACS 6222, NW 1067 and WH 1105 had greater TGW at Hisar than Karnal. There was significant positive correlation between GWS and DTM and DTA at Hisar, but not at Karnal. Genotypes HD 2967, HI 1563, Kharchia 65, KRL 210, KRL 213, KRL 3–4, Raj 4229 and Raj 4238 had greater GWS at Hisar than Karnal. Similarly, there was a positive significant correlation between BYD and TGW, PHT and PTM at Hisar, but not at Karnal; KRL 210 had greater BYD at Hisar than Karnal.

#### Kumarganj-reclaimed (normal/saline) and Kumarganj-sodic (sodic) sites

There was positive significant correlation between GYD and GWS and GNS at the sodic site, but not at the reclaimed site. Similarly, there was positive significant correlation between TGW and GWS and PHT at sodic site, but not at reclaimed site. Genotypes HW 2044 and KRL 210 had greater TGW at the sodic site than at the reclaimed site, however NW 4092 and HD 2932 had similar TGW at both sites. There was positive significant correlation between GWS and GYD, TGW, PHT and BYD at the sodic site, but not at the reclaimed site; HW 2044, Kharchia 65, KRL 210 and KRL 3–4 had greater GWS at sodic site than reclaimed site.

#### Malda (normal) and Pundibari (acidic) sites

There was positive significant correlation between GYD and PTM at Pundibari, but not at Malda; approximately, 30% of genotypes had greater GYD at Pundibari than Malda. Similarly, there was positive significant correlation between TGW and BYD at Pundibari, but not at Malda and most of the genotypes had greater TGW at Pundibari than Malda. There was positive significant correlation between BYD and TGW and PTM at Pundibari, but not at Malda and most of the genotypes had greater BYD at Pundibari than Malda. There was positive significant correlation between HI and PHT and DTM at Pundibari, but not at Malda. Genotypes DBW 14, DPW 621–50, HI 8498, HW 2044, Kharchia 65, KRL 3–4, Raj 4229, Raj 4238 and WH 1105 had greater HI at Pundibari than Malda.

### Some traits are more amenable to site-specific selection

#### Saline/sodic sites

In general, salinity/sodicity reduced the GYD, TGW, GWS, GNS, HI and BYD. Reduction in TGW in wheat under salt stress was reported by Noori *et al*. [31], Turki *et al*. [32] and Chamekh *et al*. [33]. Mass and Grieve [34] reported that high salt (NaCl) in the soil decreased spikelets per spike, thus decreasing GNS at the vegetative stage. Salinity shortened the grain filling duration which cause reduction in grain number and weight [35]. These observations are consistent with previous studies of yield component traits under hostile soil conditions [36–39]. Given that GYD correlated positively with TGW, GWS, BYD, GNS and PTM under saline and sodic soils, routine selection based on these traits could improve grain yield under hostile soils. In particular, spike associated traits like GNS and GWS could be favourable traits for selecting high performing genotypes under hostile soils, because they increase the grain yield. DTA and DTM had negative significant correlations with GYD at Kumarganj-sodic and Kumarganj-reclaimed sites [39, 33]. Early flowering and maturity might favour the grain yield under hostile soils or a way to escape salt stresses [40].

Notably, some genotypes performed better for yield and component traits under salt affected soils. KRL 3–4 had a greater GYD at Hisar than Karnal and KRL 210 had the similar GYD at Hisar and Karnal sites. These varieties have Kharchia 65 background, which have been selected from salt affected areas of Rajasthan, India [41, 42]. The better performance of these genotypes is generally due to site-specific greater TGW, GWS, GNS and BYD. Beneficial salt tolerance mechanisms which might have been transferred into these genotypes from Kharchia 65 include high leaf K^+^ and K^+^:Na^+^ concentrations and water content [43]. An ability to retain more K^+^ in the leaves by excluding Na^+^ and maintaining a higher K^+^:Na^+^ ratio helps to retain water and is the subject of extensive studies for breeding salt tolerance [44, 45, 15, 46].

#### Acidic sites

Overall, most of the genotypes showed reduced GYD, PHT and HI at Pundibari than Malda. Genotypes KRL 210, KRL 213 and HD 2967 performed well on the acid soil by maintaining greater TGW, DTM and BYD, which might be useful targets for site-specific selection. Acidic soils affect plant growth by reducing uptake of water and essential nutrients and by increases in solubility of toxic metals in the soil solution [47, 23, 48]. Tolerant genotypes might therefore have increased Al tolerance by exudation of citrate and malate organic acids which bond to Al and form non available organic acid and prevent Al to affect root apices [49–51]. Most genotypes showed increased TGW, GWS and GNS at Pundibari than Malda. This contrasts with findings of Rahman *et al*. [52] who reported reduction in TGW, GWS and GNS under acidic soils. The presence of higher amount of organic matter in acid soils at Pundibari compared to many acid soils might offset the phytotoxicity of Al through the formation of complexes with Al which are less available to plants [53]. Such phenomenon are likely to be important for site-specific selection, especially for low-input systems, e.g. under Conservation Agriculture [54]. Durum genotypes (HI 8498 and PDW 314) showed extreme sensitivity for acidic soils and had the lowest yield at Pundibari. These observations are consistent with studies of Tang *et al*. [24] who also showed durum wheat was sensitive for acidic soils.

## Supporting information

S1 Table Weather data of five sites in 2013/14 and 2014/15. Data are means of minimum and maximum temperatures and total rainfall at pre-heading and post-heading in 2013/14 and 2014/15. Data for Malda are not available.
(PDF)

S2 Table Raw data of yield and component traits of 36 genotypes evaluated at six sites over two years (2013/14 and 2014/15). (PDF)

S3 Table Two-way analysis of variance (ANOVA) for assessing the variation in traits due to genotype and site factors between sites in each growing regions. (PDF)
