# Correction: Characterising variation in wheat traits under hostile soil conditions in India

**DOI:** 10.1371/journal.pone.0196168

**Published:** 2018-04-17

**Authors:** Jaswant S. Khokhar, Sindhu Sareen, Bhudeva S. Tyagi, Gyanendra Singh, Apurba K. Chowdhury, Tapamay Dhar, Vinod Singh, Ian P. King, Scott D. Young, Martin R. Broadley

There is an error in Table 2. Please see the corrected Table 2 here.

**Table 2:** Wheat genotypes used in field trials during 2013/14 and 2014/15.

Variety	Pedigree	Date of release	Adoption Area^a^	Production Condition^b^
**Kharchia-65**	Kharchia Local/EG 953	1970	All zones	TS,IR
**HD-2009**	LR 64 A / NAI 60	1975	NWPZ	TS,IR
**UP-262**	S 308/BJ 66	1978	NEPZ	TS,IR
**KRL 1–4**	Kharchia 65/WL 711	1990	All zones	TS,IR
**HI-8498**	CR ‘S’-GS’S’//A-9-30-1/Raj 911	1999	CZ	TS,IR
**HW-2044**	HD 226*5/Sunstar*6/C-80-1	1999	SHZ	TS,IR
**KRL-19**	PBW 255/KRL 1–4	2000	All zones	TS,IR
**HD-2733**	Attila/3/Tui/Carc//Chen/Chto/4/Atila	2001	NEPZ	TS,IR
**GW-322**	PBW 173/GW 196	2002	CZ,PZ	TS,IR
**DBW-14**	Raj 3765/PBW 343	2002	NEPZ	LS,IR
**NW-1067**	TR 380-16-3-614/chat’S’	2004	UP	TS,IR
**DBW-16**	Raj 3765/WR 484//HUW 468	2006	NEPZ	TS,IR
**K-0307**	K 8321/UP 2003	2007	NEPZ	TS,IR
**DBW-17**	Cmh79a.95/3*Cno79//Raj 3777	2007	NWPZ	TS, IR
**WH-1021**	Nyot 95/Sonak	2007	NWPZ	LS,IR
**HD-2932**	Kauz/Star//HD 2643	2008	CZ	TS,IR
**CBW-38**	Cndo/r143//Ente/Mexi-2/3/Ae.squarrosa(taus)/4/Weaver/5/2*Pastor	2008	NEPZ	TS,IR
**DBW-39**	Attila/Hui	2010	NEPZ	TS,IR
**DBW-51**	Site/Milan	2010	NEPZ	LS,IR
**KRL-213**	Cndo/r143//Ente/Mexi-2/3Aegilops squarrosa(taus)/4/Weaver/5/2*Kauz	2010	NWPZ	TS,IR
**PDW-314**	Ajaia12/F3Local(SEL.Ethio.135.85)//Platai 13/3/Somat3/4/Sooty/Rascon37	2010	NWPZ	TS,IR
**KRL-210**	PBW 65/2*Pastor	2010	NWPZ/NEPZ	TS,IR
**MACS-6222**	HD 2189*2//MACS 2496	2010	PZ	TS,IR
**HI-1563**	MACS 2496*2MC 10	2011	NEPZ	LS,IR
**HD-2967**	Ald/Coc//User/HD 2160m/HD22778	2011	NWPZ	TS,IR
**DPW-621-50**	Kauz//Altar84/aOS/3/Milan/Kauz/4/Huites	2011	NWPZ	TS,IR
**RAJ-4238**	HW 2021/Raj 3765	2012	CZ	LS,IR
**RAJ-4229**	HW 2048/Raj 4000	2012	NEPZ	TS,IR
**NW-4018**	-	2013	NWPZ	LS,IR
**WH-1105**	Milan/S87230//Babax	2013	NWPZ	TS,IR
**DBW-71**	Prinia/UP 2425	2013	NWPZ	LS,IR
**RW-3684**	Pastor.florkwa.1//Pastor	Stock	-	TS,RF
**NW-4092**	Site/MO/Milan/3/PBW 343	Stock	NWPZ	LS,IR
**KRL 3–4**	HD 1982/Kharchia 65	^c^Stock (2009)	All zones	TS,IR
**BH-1146**	Ponta grossa 1//Fronteira/Mentana	Stock (1987)	-	-
**DBW-46**	PBW 343/Inq21	Stock (2011)	-	-

^a^NEPZ, North Eastern Plain Zone; NWPZ, North Western Plain Zone; CZ, Central Zone; PZ, Peninsular Zone; SHZ, Southern Hills Zone

^b^TS, Timely Sown; LS, Late Sown; IR, Irrigated; RF, Rain fed

^c^Stock refers to advanced breeding line used in breeding programme
