# Supplementary material for: Characterising variation in wheat traits under hostile soil conditions in India
Source: PLoS One. 2017 Jun 12;12(6):e0179208. doi: 10.1371/journal.pone.0179208 (PMC5467898; doi:10.1371/journal.pone.0179208)
Supplement: S1 Table — Data are means of minimum and maximum temperatures and total rainfall at pre-heading and post-heading in 2013/14 and 2014/15. Data for Malda are not available. (PDF) [file pone.0179208.s001.pdf]

Supplementary Table 1. Weather data of five sites in 2013/14 and 2014/15. Data are means of minimum and maximum temperatures and total rainfall at pre-heading and post-heading in 2013/14 and 2014/15. Data for Malda are not available.

	2013-14						2014-15					
	Pre-heading			Post-heading			Pre-heading			Post-heading		
Locations	Min. temp.	Max. temp.	Total rainfall	Min. temp.	Max. temp.	Total rainfall	Min. temp.	Max. temp.	Total rainfall	Min. temp.	Max. temp.	Total rainfall
**Karnal**	7.55	20.70	67.60	11.46	25.53	112.20	7.49	19.22	21.40	14.84	26.83	419.40
**Hisar**	6.97	21.27	4.20	10.81	25.25	65.80	6.49	19.72	24.40	13.03	27.11	218.40
**Kumarganj-reclaimed**	8.30	21.64	64.30	13.19	30.17	57.40	7.74	17.86	80.10	14.73	29.23	68.80
**Kumarganj-sodic**	8.15	21.20	64.30	13.61	30.68	57.40	7.75	17.65	80.10	14.73	29.23	68.80
**Pundibari**	11.51	22.60	0.00	13.65	26.25	34.00	11.93	23.02	22.50	15.90	29.19	68.90
