# Supplementary material for: Characterising variation in wheat traits under hostile soil conditions in India
Source: PLoS One. 2017 Jun 12;12(6):e0179208. doi: 10.1371/journal.pone.0179208 (PMC5467898; doi:10.1371/journal.pone.0179208)
Supplement: S2 Table — (PDF) [file pone.0179208.s002.pdf]

Supplementary Table 2. Raw data of yield and component traits of 36 genotypes evaluated at six sites over two years (2013/14 and 2014/15).

Samples!	Year!	Year1!	Year2!	Site_Name!	Site2014!	site2015!	ZONE!	Soil!	HOSTILE_NOT!	SOIL_ pH!	Replication!	Genotype!	Species!	Plot_No!	DTH	DTA	PHT	PTM	DTM	TGW	GNS	GWS	GYD	BYD	HI
1	2014	2014		KARNAL	KARNAL		NWPZ	NORMAL	NOT_HOSTILE	7.5	1	WH_1105	Triticum_aestivum	1	95	103	95	93	138	38.35	62	2.4	8.0	4950	40.5
2	2014	2014		KARNAL	KARNAL		NWPZ	NORMAL	NOT_HOSTILE	7.5	1	NW_4092	Triticum_aestivum	2	99	105	108	89	139	36.46	63	2.3	6.4	4900	32.9
3	2014	2014		KARNAL	KARNAL		NWPZ	NORMAL	NOT_HOSTILE	7.5	1	DBW_16	Triticum_aestivum	3	97	104	96	117	140	38.64	41	1.6	5.4	4200	32.4
4	2014	2014		KARNAL	KARNAL		NWPZ	NORMAL	NOT_HOSTILE	7.5	1	NW_4018	Triticum_aestivum	4	96	104	108	88	136	33.52	63	2.1	6.4	4450	35.8
5	2014	2014		KARNAL	KARNAL		NWPZ	NORMAL	NOT_HOSTILE	7.5	1	HD_2733	Triticum_aestivum	5	97	103	100	128	136	32.15	40	1.3	3.9	3500	27.7
6	2014	2014		KARNAL	KARNAL		NWPZ	NORMAL	NOT_HOSTILE	7.5	1	DBW_39	Triticum_aestivum	6	93	102	100	117	137	41.35	47	2.0	7.5	5050	37.2
7	2014	2014		KARNAL	KARNAL		NWPZ	NORMAL	NOT_HOSTILE	7.5	1	CBW_38	Triticum_aestivum	7	94	102	105	122	137	40.83	43	1.8	7.2	5150	35.0
8	2014	2014		KARNAL	KARNAL		NWPZ	NORMAL	NOT_HOSTILE	7.5	1	NW_1067	Triticum_aestivum	8	94	103	94	75	139	33.57	57	1.9	5.3	4050	32.8
9	2014	2014		KARNAL	KARNAL		NWPZ	NORMAL	NOT_HOSTILE	7.5	1	DBW_51	Triticum_aestivum	9	93	102	110	74	139	44.18	47	2.1	6.6	4500	36.9
10	2014	2014		KARNAL	KARNAL		NWPZ	NORMAL	NOT_HOSTILE	7.5	1	KRL_19	Triticum_aestivum	10	85	98	93	95	134	28.69	58	1.7	3.9	3700	26.1
11	2014	2014		KARNAL	KARNAL		NWPZ	NORMAL	NOT_HOSTILE	7.5	1	HI_1563	Triticum_aestivum	11	85	97	96	77	131	39.50	44	1.7	5.8	4500	32.1
12	2014	2014		KARNAL	KARNAL		NWPZ	NORMAL	NOT_HOSTILE	7.5	1	KHARCHIA_65	Triticum_aestivum	12	90	100	133	124	138	34.76	49	1.7	1.4	3650	9.3
13	2014	2014		KARNAL	KARNAL		NWPZ	NORMAL	NOT_HOSTILE	7.5	1	HW_2044	Triticum_aestivum	13	82	93	96	137	130	45.53	53	2.4	6.1	4300	35.6
14	2014	2014		KARNAL	KARNAL		NWPZ	NORMAL	NOT_HOSTILE	7.5	1	DBW_46	Triticum_aestivum	14	97	104	116	115	138	44.26	52	2.3	6.1	4700	32.6
15	2014	2014		KARNAL	KARNAL		NWPZ	NORMAL	NOT_HOSTILE	7.5	1	RAJ_4238	Triticum_aestivum	15	84	98	93	91	131	40.23	43	1.7	5.3	3950	33.7
16	2014	2014		KARNAL	KARNAL		NWPZ	NORMAL	NOT_HOSTILE	7.5	1	RAJ_4229	Triticum_aestivum	16	85	98	90	80	132	43.48	41	1.8	5.9	3900	37.9
17	2014	2014		KARNAL	KARNAL		NWPZ	NORMAL	NOT_HOSTILE	7.5	1	HI_8498	Triticum_turgidum	17	97	105	89	73	140	50.67	42	2.1	5.9	3950	37.2
18	2014	2014		KARNAL	KARNAL		NWPZ	NORMAL	NOT_HOSTILE	7.5	1	KRL_1-4	Triticum_aestivum	18	86	96	100	102	134	39.02	53	2.1	6.1	4250	35.8
19	2014	2014		KARNAL	KARNAL		NWPZ	NORMAL	NOT_HOSTILE	7.5	1	UP_262	Triticum_aestivum	19	91	97	107	121	138	38.07	33	1.3	4.3	3700	29.2
20	2014	2014		KARNAL	KARNAL		NWPZ	NORMAL	NOT_HOSTILE	7.5	1	DPW_621-50	Triticum_aestivum	20	96	104	99	110	139	42.26	50	2.1	7.3	4700	38.9
21	2014	2014		KARNAL	KARNAL		NWPZ	NORMAL	NOT_HOSTILE	7.5	1	KRL_213	Triticum_aestivum	21	98	104	94	96	140	33.70	62	2.1	5.9	4200	34.9
22	2014	2014		KARNAL	KARNAL		NWPZ	NORMAL	NOT_HOSTILE	7.5	1	KRL_3-4	Triticum_aestivum	22	91	99	123	145	134	24.74	27	0.7	0.6	3000	4.7
23	2014	2014		KARNAL	KARNAL		NWPZ	NORMAL	NOT_HOSTILE	7.5	1	HD_2967	Triticum_aestivum	23	96	103	102	102	140	42.92	51	2.2	6.7	4500	37.3
24	2014	2014		KARNAL	KARNAL		NWPZ	NORMAL	NOT_HOSTILE	7.5	1	GW_322	Triticum_aestivum	24	92	102	98	108	135	41.24	50	2.1	5.7	4250	33.3
25	2014	2014		KARNAL	KARNAL		NWPZ	NORMAL	NOT_HOSTILE	7.5	1	WH_1021	Triticum_aestivum	25	91	99	108	121	136	35.57	49	1.7	6.7	4950	33.6
26	2014	2014		KARNAL	KARNAL		NWPZ	NORMAL	NOT_HOSTILE	7.5	1	HD_2932	Triticum_aestivum	26	92	102	97	99	139	35.92	52	1.9	4.8	4100	29.3
27	2014	2014		KARNAL	KARNAL		NWPZ	NORMAL	NOT_HOSTILE	7.5	1	MACS_6222	Triticum_aestivum	27	97	105	98	78	139	38.01	57	2.2	5.4	4050	33.1
28	2014	2014		KARNAL	KARNAL		NWPZ	NORMAL	NOT_HOSTILE	7.5	1	DBW_17	Triticum_aestivum	28	93	101	87	99	135	39.20	50	2.0	5.8	3750	38.9
29	2014	2014		KARNAL	KARNAL		NWPZ	NORMAL	NOT_HOSTILE	7.5	1	RW_3684	Triticum_aestivum	29	97	103	107	93	138	41.78	46	1.9	7.1	4550	38.8
30	2014	2014		KARNAL	KARNAL		NWPZ	NORMAL	NOT_HOSTILE	7.5	1	PDW_314	Triticum_turgidum	30	102	110	92	91	140	44.44	52	2.3	6.5	4250	38.1
31	2014	2014		KARNAL	KARNAL		NWPZ	NORMAL	NOT_HOSTILE	7.5	1	DBW_14	Triticum_aestivum	31	83	91	82	96	128	44.88	34	1.5	5.5	3450	40.1
32	2014	2014		KARNAL	KARNAL		NWPZ	NORMAL	NOT_HOSTILE	7.5	1	KRL_210	Triticum_aestivum	32	91	98	95	78	135	45.90	43	2.0	5.8	3700	39.1
33	2014	2014		KARNAL	KARNAL		NWPZ	NORMAL	NOT_HOSTILE	7.5	1	HD_2009	Triticum_aestivum	33	91	98	117	125	134	41.29	45	1.9	6.3	4500	34.8
34	2014	2014		KARNAL	KARNAL		NWPZ	NORMAL	NOT_HOSTILE	7.5	1	DBW_71	Triticum_aestivum	34	86	97	94	111	133	45.29	42	1.9	5.6	3450	40.9
35	2014	2014		KARNAL	KARNAL		NWPZ	NORMAL	NOT_HOSTILE	7.5	1	BH_1146	Triticum_aestivum	35	91	97	147	113	133	46.88	38	1.8	5.1	4900	25.8
36	2014	2014		KARNAL	KARNAL		NWPZ	NORMAL	NOT_HOSTILE	7.5	1	K_0307	Triticum_aestivum	36	96	103	113	136	139	41.66	64	2.7	7.1	5050	35.1
37	2014	2014		KARNAL	KARNAL		NWPZ	NORMAL	NOT_HOSTILE	7.5	2	GW_322	Triticum_aestivum	37	91	101	94	116	136	35.71	52	1.9	4.9	3850	31.8
38	2014	2014		KARNAL	KARNAL		NWPZ	NORMAL	NOT_HOSTILE	7.5	2	WH_1105	Triticum_aestivum	38	94	103	96	100	137	38.41	64	2.5	6.7	4250	39.4
39	2014	2014		KARNAL	KARNAL		NWPZ	NORMAL	NOT_HOSTILE	7.5	2	DBW_17	Triticum_aestivum	39	92	100	92	112	136	33.63	43	1.4	4.8	3450	34.7
40	2014	2014		KARNAL	KARNAL		NWPZ	NORMAL	NOT_HOSTILE	7.5	2	BH_1146	Triticum_aestivum	40	91	97	142	129	135	44.78	36	1.6	4.9	4400	27.6
41	2014	2014		KARNAL	KARNAL		NWPZ	NORMAL	NOT_HOSTILE	7.5	2	RAJ_4229	Triticum_aestivum	41	85	100	91	133	134	42.32	48	2.0	5.1	3900	32.9
42	2014	2014		KARNAL	KARNAL		NWPZ	NORMAL	NOT_HOSTILE	7.5	2	NW_1067	Triticum_aestivum	42	95	104	90	115	139	33.24	50	1.7	5.4	4450	30.6
43	2014	2014		KARNAL	KARNAL		NWPZ	NORMAL	NOT_HOSTILE	7.5	2	HI_1563	Triticum_aestivum	43	83	93	94	140	135	31.35	47	1.5	4.5	3850	28.9
44	2014	2014		KARNAL	KARNAL		NWPZ	NORMAL	NOT_HOSTILE	7.5	2	RAJ_4238	Triticum_aestivum	44	84	100	93	96	134	35.89	47	1.7	4.1	3400	30.4
45	2014	2014		KARNAL	KARNAL		NWPZ	NORMAL	NOT_HOSTILE	7.5	2	KRL_213	Triticum_aestivum	45	97	104	93	93	140	31.65	55	1.7	5.6	4250	32.7
46	2014	2014		KARNAL	KARNAL		NWPZ	NORMAL	NOT_HOSTILE	7.5	2	HD_2932	Triticum_aestivum	46	92	102	96	85	138	34.73	45	1.6	4.6	4000	28.8
47	2014	2014		KARNAL	KARNAL		NWPZ	NORMAL	NOT_HOSTILE	7.5	2	K_0307	Triticum_aestivum	47	96	103	111	90	138	39.25	43	1.7	4.9	3700	33.1
48	2014	2014		KARNAL	KARNAL		NWPZ	NORMAL	NOT_HOSTILE	7.5	2	NW_4018	Triticum_aestivum	48	96	104	111	74	137	40.49	49	2.0	6.6	4600	35.8
49	2014	2014		KARNAL	KARNAL		NWPZ	NORMAL	NOT_HOSTILE	7.5	2	WH_1021	Triticum_aestivum	49	90	99	109	147	135	40.88	50	2.0	6.8	4950	34.3
50	2014	2014		KARNAL	KARNAL		NWPZ	NORMAL	NOT_HOSTILE	7.5	2	DBW_51	Triticum_aestivum	50	94	102	109	74	137	46.84	49	2.3	6.0	3950	38.1
51	2014	2014		KARNAL	KARNAL		NWPZ	NORMAL	NOT_HOSTILE	7.5	2	HD_2733	Triticum_aestivum	51	96	103	97	94	137	43.62	40	1.7	4.9	3950	31.2
52	2014	2014		KARNAL	KARNAL		NWPZ	NORMAL	NOT_HOSTILE	7.5	2	HW_2044	Triticum_aestivum	52	82	93	95	89	132	37.60	51	1.9	5.3	3850	34.7
53	2014	2014		KARNAL	KARNAL		NWPZ	NORMAL	NOT_HOSTILE	7.5	2	DPW_621-50	Triticum_aestivum	53	96	104	101	102	137	43.71	51	2.2	7.2	4850	37.0
54	2014	2014		KARNAL	KARNAL		NWPZ	NORMAL	NOT_HOSTILE	7.5	2	DBW_71	Triticum_aestivum	54	86	97	97	102	132	45.30	43	2.0	6.6	3950	41.9
55	2014	2014		KARNAL	KARNAL		NWPZ	NORMAL	NOT_HOSTILE	7.5	2	KHARCHIA_65	Triticum_aestivum	55	91	101	125	139	137	14.93	15	0.2	0.3	2750	2.5
56	2014	2014		KARNAL	KARNAL		NWPZ	NORMAL	NOT_HOSTILE	7.5	2	UP_262	Triticum_aestivum	56	91	98	109	74	137	39.43	39	1.5	3.0	3100	24.0
57	2014	2014		KARNAL	KARNAL		NWPZ	NORMAL	NOT_HOSTILE	7.5	2	DBW_46	Triticum_aestivum	57	97	105	110	82	138	46.22	48	2.2	6.2	4150	37.2
58	2014	2014		KARNAL	KARNAL		NWPZ	NORMAL	NOT_HOSTILE	7.5	2	MACS_6222	Triticum_aestivum	58	96	105	96	97	138	47.44	55	2.6	6.0	3950	37.8
59	2014	2014		KARNAL	KARNAL		NWPZ	NORMAL	NOT_HOSTILE	7.5	2	DBW_16	Triticum_aestivum	59	97	105	93	117	139	38.97	46	1.8	4.3	3200	33.6
60	2014	2014		KARNAL	KARNAL		NWPZ	NORMAL	NOT_HOSTILE	7.5	2	HD_2009	Triticum_aestivum	60	91	99	112	153	135	36.28	53	1.9	5.4	4500	30.0
61	2014	2014		KARNAL	KARNAL		NWPZ	NORMAL	NOT_HOSTILE	7.5	2	KRL_210	Triticum_aestivum	61	90	98	98	77	136	45.47	46	2.1	7.9	4900	40.5
62	2014	2014		KARNAL	KARNAL		NWPZ	NORMAL	NOT_HOSTILE	7.5	2	CBW_38	Triticum_aestivum	62	95	102	100	89	137	40.36	45	1.8	6.0	4200	35.8
63	2014	2014		KARNAL	KARNAL		NWPZ	NORMAL	NOT_HOSTILE	7.5	2	PDW_314	Triticum_turgidum	63	102	110	92	118	140	35.60	49	1.7	5.6	4000	35.1
64	2014	2014		KARNAL	KARNAL		NWPZ	NORMAL	NOT_HOSTILE	7.5	2	KRL_3-4	Triticum_aestivum	64	91	99	128	108	136	26.60	39	1.0	1.0	3100	8.1
65	2014	2014		KARNAL	KARNAL		NWPZ	NORMAL	NOT_HOSTILE	7.5	2	HI_8498	Triticum_turgidum	65	98	105	90	78	140	50.28	44	2.2	5.1	3550	36.3
66	2014	2014		KARNAL	KARNAL		NWPZ	NORMAL	NOT_HOSTILE	7.5	2	NW_4092	Triticum_aestivum	66	98	104	110	89	139	30.89	56	1.7	5.5	4700	29.3
67	2014	2014		KARNAL	KARNAL		NWPZ	NORMAL	NOT_HOSTILE	7.5	2	KRL_1-4	Triticum_aestivum	67	86	96	102	91	135	45.25	44	2.0	6.6	4500	36.5
68	2014	2014		KARNAL	KARNAL		NWPZ	NORMAL	NOT_HOSTILE	7.5	2	DBW_14	Triticum_aestivum	68	83	93	85	88	129	43.09	38	1.6	5.4	3650	36.8
69	2014	2014		KARNAL	KARNAL		NWPZ	NORMAL	NOT_HOSTILE	7.5	2	DBW_39	Triticum_aestivum	69	93	101	104	95	136	44.86	49	2.2	7.1	4600	38.6
70	2014	2014		KARNAL	KARNAL		NWPZ	NORMAL	NOT_HOSTILE	7.5	2	HD_2967	Triticum_aestivum	70	96	103	100	68	137	45.51	41	1.9	7.2	4600	38.9
71	2014	2014		KARNAL	KARNAL		NWPZ	NORMAL	NOT_HOSTILE	7.5	2	KRL_19	Triticum_aestivum	71	85	99	90	83	134	31.82	49	1.6	4.6	3450	33.4
72	2014	2014		KARNAL	KARNAL		NWPZ	NORMAL	NOT_HOSTILE	7.5	2	RW_3684	Triticum_aestivum	72	96	102	104	120	138	43.33	43	1.9	8.4	5200	40.5
73	2014	2014		HISAR	HISAR		NWPZ	SALINE	HOSTILE	8.1	1	KRL_3-4	Triticum_aestivum	1	95	100	124	112	140	40.7	37	1.5	3.0	2700	28.1
74	2014	2014		HISAR	HISAR		NWPZ	SALINE	HOSTILE	8.1	1	KRL_19	Triticum_aestivum	2	95	99	85	66	140	25.3	58	1.5	2.2	2400	22.5
75	2014	2014		HISAR	HISAR		NWPZ	SALINE	HOSTILE	8.1	1	GW_322	Triticum_aestivum	3	96	100	93	103	140	56.2	41	2.3	4.2	3000	35.0
76	2014	2014		HISAR	HISAR		NWPZ	SALINE	HOSTILE	8.1	1	NW_1067	Triticum_aestivum	4	99	103	91	87	143	33.2	62	2.1	4.3	3300	32.8
77	2014	2014		HISAR	HISAR		NWPZ	SALINE	HOSTILE	8.1	1	DBW_17	Triticum_aestivum	5	97	102	86	97	139	36.7	53	1.9	4.4	3000	36.5
78	2014	2014		HISAR	HISAR		NWPZ	SALINE	HOSTILE	8.1	1	HD_2967	Triticum_aestivum	6	97	102	98	87	139	38.5	57	2.2	5.7	3750	37.8
79	2014	2014		HISAR	HISAR		NWPZ	SALINE	HOSTILE	8.1	1	KRL_1-4	Triticum_aestivum	7	101	106	95	66	141	29.6	49	1.5	2.5	2000	31.8
80	2014	2014		HISAR	HISAR		NWPZ	SALINE	HOSTILE	8.1	1	HW_2044	Triticum_aestivum	8	97	102	98	85	141	42.1	56	2.4	4.2	2750	38.2
81	2014	2014		HISAR	HISAR		NWPZ	SALINE	HOSTILE	8.1	1	RAJ_4238	Triticum_aestivum	9	100	107	86	55	141	37.6	55	2.1	4.1	2600	39.6
82	2014	2014		HISAR	HISAR		NWPZ	SALINE	HOSTILE	8.1	1	HD_2932	Triticum_aestivum	10	100	107	96	88	143	32.6	53	1.7	3.9	3250	29.8
83	2014	2014		HISAR	HISAR		NWPZ	SALINE	HOSTILE	8.1	1	UP_262	Triticum_aestivum	11	99	104	111	90	142	39.5	44	1.7	4.4	3600	30.3
84	2014	2014		HISAR	HISAR		NWPZ	SALINE	HOSTILE	8.1	1	PDW_314	Triticum_turgidum	12	110	115	91	102	145	42.4	55	2.3	5.8	3750	38.4
85	2014	2014		HISAR	HISAR		NWPZ	SALINE	HOSTILE	8.1	1	BH_1146	Triticum_aestivum	13	97	102	127	92	142	39.4	43	1.7	3.3	2800	29.6
86	2014	2014		HISAR	HISAR		NWPZ	SALINE	HOSTILE	8.1	1	K_0307	Triticum_aestivum	14	104	108	104	103	144	40.8	57	2.3	3.7	2500	37.4
87	2014	2014		HISAR	HISAR		NWPZ	SALINE	HOSTILE	8.1	1	DBW_71	Triticum_aestivum	15	99	103	92	66	144	42.7	58	2.5	4.8	3000	40.0
88	2014	2014		HISAR	HISAR		NWPZ	SALINE	HOSTILE	8.1	1	NW_4092	Triticum_aestivum	16	102	108	101	69	145	38.3	70	2.7	3.6	2600	34.3
89	2014	2014		HISAR	HISAR		NWPZ	SALINE	HOSTILE	8.1	1	CBW_38	Triticum_aestivum	17	99	103	100	101	143	39.9	57	2.3	5.4	3500	38.6
90	2014	2014		HISAR	HISAR		NWPZ	SALINE	HOSTILE	8.1	1	DBW_46	Triticum_aestivum	18	101	107	102	79	145	39.0	75	2.9	5.0	3600	34.9
91	2014	2014		HISAR	HISAR		NWPZ	SALINE	HOSTILE	8.1	1	HD_2733	Triticum_aestivum	19	101	107	88	102	142	42.3	47	2.0	4.6	3300	34.5
92	2014	2014		HISAR	HISAR		NWPZ	SALINE	HOSTILE	8.1	1	DBW_51	Triticum_aestivum	20	97	101	98	123	144	43.2	53	2.3	4.2	3600	29.2
93	2014	2014		HISAR	HISAR		NWPZ	SALINE	HOSTILE	8.1	1	NW_4018	Triticum_aestivum	21	103	109	102	95	145	38.9	57	2.2	4.2	4000	26.0
94	2014	2014		HISAR	HISAR		NWPZ	SALINE	HOSTILE	8.1	1	HI_1563	Triticum_aestivum	22	97	102	91	80	146	38.9	69	2.7	4.4	4000	27.3
95	2014	2014		HISAR	HISAR		NWPZ	SALINE	HOSTILE	8.1	1	RAJ_4229	Triticum_aestivum	23	96	103	88	87	144	40.3	61	2.5	3.4	3200	26.6
96	2014	2014		HISAR	HISAR		NWPZ	SALINE	HOSTILE	8.1	1	HD_2009	Triticum_aestivum	24	97	102	101	87	145	46.9	54	2.5	4.0	5600	18.0
97	2014	2014		HISAR	HISAR		NWPZ	SALINE	HOSTILE	8.1	1	HI_8498	Triticum_turgidum	25	107	113	90	51	146	50.0	61	3.1	4.5	3100	36.5
98	2014	2014		HISAR	HISAR		NWPZ	SALINE	HOSTILE	8.1	1	RW_3684	Triticum_aestivum	26	101	106	106	102	144	41.9	54	2.3	5.0	3900	32.2
99	2014	2014		HISAR	HISAR		NWPZ	SALINE	HOSTILE	8.1	1	WH_1105	Triticum_aestivum	27	102	108	94	87	144	41.1	64	2.6	5.8	4200	34.3
100	2014	2014		HISAR	HISAR		NWPZ	SALINE	HOSTILE	8.1	1	DPW_621-50	Triticum_aestivum	28	100	105	100	110	146	42.4	48	2.0	5.0	5000	25.0
101	2014	2014		HISAR	HISAR		NWPZ	SALINE	HOSTILE	8.1	1	DBW_14	Triticum_aestivum	29	95	104	99	105	144	38.1	43	1.6	2.4	3500	17.4
102	2014	2014		HISAR	HISAR		NWPZ	SALINE	HOSTILE	8.1	1	KRL_210	Triticum_aestivum	30	99	105	99	172	144	40.3	53	2.1	6.6	5500	29.9
103	2014	2014		HISAR	HISAR		NWPZ	SALINE	HOSTILE	8.1	1	DBW_16	Triticum_aestivum	31	99	103	95	86	142	33.2	57	1.9	4.1	3000	34.0
104	2014	2014		HISAR	HISAR		NWPZ	SALINE	HOSTILE	8.1	1	MACS_6222	Triticum_aestivum	32	100	105	98	97	145	41.3	52	2.2	4.8	4100	29.2
105	2014	2014		HISAR	HISAR		NWPZ	SALINE	HOSTILE	8.1	1	WH_1021	Triticum_aestivum	33	100	105	101	127	146	40.7	45	1.8	4.2	5700	18.3
106	2014	2014		HISAR	HISAR		NWPZ	SALINE	HOSTILE	8.1	1	KRL_213	Triticum_aestivum	34	102	107	99	82	147	34.8	70	2.4	3.1	4000	19.2
107	2014	2014		HISAR	HISAR		NWPZ	SALINE	HOSTILE	8.1	1	KHARCHIA_65	Triticum_aestivum	35	99	104	127	95	144	37.3	46	1.7	1.3	3800	8.3
108	2014	2014		HISAR	HISAR		NWPZ	SALINE	HOSTILE	8.1	1	DBW_39	Triticum_aestivum	36	99	105	108	104	142	48.2	48	2.3	6.0	5000	29.8
109	2014	2014		HISAR	HISAR		NWPZ	SALINE	HOSTILE	8.1	2	WH_1021	Triticum_aestivum	37	99	103	104	84	140	41.0	47	1.9	5.0	3750	33.1
110	2014	2014		HISAR	HISAR		NWPZ	SALINE	HOSTILE	8.1	2	DBW_51	Triticum_aestivum	38	97	101	102	66	141	39.3	53	2.1	3.9	3000	32.8
111	2014	2014		HISAR	HISAR		NWPZ	SALINE	HOSTILE	8.1	2	DBW_46	Triticum_aestivum	39	100	106	104	83	142	43.0	44	1.9	3.5	3300	26.2
112	2014	2014		HISAR	HISAR		NWPZ	SALINE	HOSTILE	8.1	2	DPW_621-50	Triticum_aestivum	40	101	106	93	68	141	42.0	52	2.2	4.0	2900	34.4
113	2014	2014		HISAR	HISAR		NWPZ	SALINE	HOSTILE	8.1	2	HD_2932	Triticum_aestivum	41	99	107	98	83	139	38.2	49	1.9	3.2	3200	25.2
114	2014	2014		HISAR	HISAR		NWPZ	SALINE	HOSTILE	8.1	2	GW_322	Triticum_aestivum	42	96	101	93	75	139	39.0	57	2.2	4.2	3000	35.1
115	2014	2014		HISAR	HISAR		NWPZ	SALINE	HOSTILE	8.1	2	PDW_314	Triticum_turgidum	43	108	114	92	90	145	44.0	55	2.4	4.0	2900	34.1
116	2014	2014		HISAR	HISAR		NWPZ	SALINE	HOSTILE	8.1	2	KRL_3-4	Triticum_aestivum	44	96	102	126	79	142	38.0	41	1.6	3.2	3000	27.0
117	2014	2014		HISAR	HISAR		NWPZ	SALINE	HOSTILE	8.1	2	KHARCHIA_65	Triticum_aestivum	45	100	106	122	93	142	34.3	35	1.2	2.2	2500	21.6
118	2014	2014		HISAR	HISAR		NWPZ	SALINE	HOSTILE	8.1	2	HD_2009	Triticum_aestivum	46	97	102	114	77	142	40.8	56	2.3	3.7	3500	26.3
119	2014	2014		HISAR	HISAR		NWPZ	SALINE	HOSTILE	8.1	2	NW_4092	Triticum_aestivum	47	101	109	103	60	142	37.1	57	2.1	3.9	3000	32.3
120	2014	2014		HISAR	HISAR		NWPZ	SALINE	HOSTILE	8.1	2	RW_3684	Triticum_aestivum	48	101	108	103	67	143	36.2	59	2.1	5.1	3500	36.2
121	2014	2014		HISAR	HISAR		NWPZ	SALINE	HOSTILE	8.1	2	HW_2044	Triticum_aestivum	49	95	101	94	73	143	40.2	51	2.0	4.6	3400	33.6
122	2014	2014		HISAR	HISAR		NWPZ	SALINE	HOSTILE	8.1	2	DBW_14	Triticum_aestivum	50	96	102	80	78	143	38.6	56	2.2	4.1	2800	36.8
123	2014	2014		HISAR	HISAR		NWPZ	SALINE	HOSTILE	8.1	2	NW_4018	Triticum_aestivum	51	103	108	98	56	143	40.8	55	2.2	4.3	3000	35.6
124	2014	2014		HISAR	HISAR		NWPZ	SALINE	HOSTILE	8.1	2	BH_1146	Triticum_aestivum	52	97	104	134	92	142	40.3	47	1.9	3.8	3000	31.3
125	2014	2014		HISAR	HISAR		NWPZ	SALINE	HOSTILE	8.1	2	HD_2967	Triticum_aestivum	53	98	105	98	69	143	38.0	68	2.6	5.1	3000	42.7
126	2014	2014		HISAR	HISAR		NWPZ	SALINE	HOSTILE	8.1	2	KRL_213	Triticum_aestivum	54	101	106	92	92	144	32.6	73	2.4	4.9	3500	35.1
127	2014	2014		HISAR	HISAR		NWPZ	SALINE	HOSTILE	8.1	2	DBW_39	Triticum_aestivum	55	99	106	97	87	142	40.0	54	2.2	4.9	4000	30.9
128	2014	2014		HISAR	HISAR		NWPZ	SALINE	HOSTILE	8.1	2	HI_1563	Triticum_aestivum	56	97	103	96	67	143	39.7	44	1.8	4.0	3200	31.5
129	2014	2014		HISAR	HISAR		NWPZ	SALINE	HOSTILE	8.1	2	UP_262	Triticum_aestivum	57	101	107	97	80	142	40.8	45	1.8	3.1	2800	27.9
130	2014	2014		HISAR	HISAR		NWPZ	SALINE	HOSTILE	8.1	2	CBW_38	Triticum_aestivum	58	100	106	97	68	143	39.2	60	2.4	5.9	4000	36.9
131	2014	2014		HISAR	HISAR		NWPZ	SALINE	HOSTILE	8.1	2	DBW_17	Triticum_aestivum	59	99	105	85	100	139	37.5	56	2.1	5.3	3700	35.6
132	2014	2014		HISAR	HISAR		NWPZ	SALINE	HOSTILE	8.1	2	KRL_210	Triticum_aestivum	60	97	104	101	104	140	41.3	55	2.3	6.6	4500	36.5
133	2014	2014		HISAR	HISAR		NWPZ	SALINE	HOSTILE	8.1	2	MACS_6222	Triticum_aestivum	61	103	108	90	98	146	42.6	53	2.3	5.3	5000	26.3
134	2014	2014		HISAR	HISAR		NWPZ	SALINE	HOSTILE	8.1	2	HD_2733	Triticum_aestivum	62	101	107	95	105	143	45.7	48	2.2	5.0	4000	31.2
135	2014	2014		HISAR	HISAR		NWPZ	SALINE	HOSTILE	8.1	2	KRL_1-4	Triticum_aestivum	63	97	104	95	63	144	31.6	54	1.7	3.6	3100	28.9
136	2014	2014		HISAR	HISAR		NWPZ	SALINE	HOSTILE	8.1	2	K_0307	Triticum_aestivum	64	102	108	104	76	145	39.6	52	2.1	4.5	3900	29.2
137	2014	2014		HISAR	HISAR		NWPZ	SALINE	HOSTILE	8.1	2	KRL_19	Triticum_aestivum	65	96	101	95	86	143	22.5	76	1.7	2.9	4100	17.6
138	2014	2014		HISAR	HISAR		NWPZ	SALINE	HOSTILE	8.1	2	HI_8498	Triticum_turgidum	66	108	114	99	86	146	51.3	55	2.8	6.1	5200	29.5
139	2014	2014		HISAR	HISAR		NWPZ	SALINE	HOSTILE	8.1	2	WH_1105	Triticum_aestivum	67	103	109	97	100	142	37.0	61	2.3	6.7	5000	33.3
140	2014	2014		HISAR	HISAR		NWPZ	SALINE	HOSTILE	8.1	2	RAJ_4229	Triticum_aestivum	68	95	100	92	75	142	42.3	49	2.1	4.0	4600	21.6
141	2014	2014		HISAR	HISAR		NWPZ	SALINE	HOSTILE	8.1	2	NW_1067	Triticum_aestivum	69	100	106	98	97	145	37.0	51	1.9	6.3	5000	31.7
142	2014	2014		HISAR	HISAR		NWPZ	SALINE	HOSTILE	8.1	2	DBW_71	Triticum_aestivum	70	96	102	101	100	146	44.3	47	2.1	5.9	5500	26.7
143	2014	2014		HISAR	HISAR		NWPZ	SALINE	HOSTILE	8.1	2	RAJ_4238	Triticum_aestivum	71	95	100	95	121	143	44.0	51	2.2	4.9	4200	29.4
144	2014	2014		HISAR	HISAR		NWPZ	SALINE	HOSTILE	8.1	2	DBW_16	Triticum_aestivum	72	99	104	95	113	144	38.0	52	2.0	4.9	5500	22.1
145	2014	2014		KUMARGANJ-RECLAIMED	KUMARGANJ-RECLAIMED		NEPZ	SALINE	NOT_HOSTILE	8.3	1	PDW_314	Triticum_turgidum	1	92	97	70	87	132	40.5	61	2.5	2.8	1800	38.7
146	2014	2014		KUMARGANJ-RECLAIMED	KUMARGANJ-RECLAIMED		NEPZ	SALINE	NOT_HOSTILE	8.3	1	HD_2967	Triticum_aestivum	2	88	92	72	80	130	38.5	62	2.4	2.2	1350	41.0
147	2014	2014		KUMARGANJ-RECLAIMED	KUMARGANJ-RECLAIMED		NEPZ	SALINE	NOT_HOSTILE	8.3	1	DPW_621-50	Triticum_aestivum	3	86	92	81	74	130	38.1	71	1.7	2.8	1700	41.5
148	2014	2014		KUMARGANJ-RECLAIMED	KUMARGANJ-RECLAIMED		NEPZ	SALINE	NOT_HOSTILE	8.3	1	DBW_17	Triticum_aestivum	4	84	88	68	81	129	37.5	56	2.1	2.5	1450	42.4
149	2014	2014		KUMARGANJ-RECLAIMED	KUMARGANJ-RECLAIMED		NEPZ	SALINE	NOT_HOSTILE	8.3	1	K_0307	Triticum_aestivum	5	84	90	84	81	130	36.4	60	2.4	1.9	1300	36.3
150	2014	2014		KUMARGANJ-RECLAIMED	KUMARGANJ-RECLAIMED		NEPZ	SALINE	NOT_HOSTILE	8.3	1	BH_1146	Triticum_aestivum	6	84	88	111	84	131	34.9	47	1.7	1.6	1150	33.8
151	2014	2014		KUMARGANJ-RECLAIMED	KUMARGANJ-RECLAIMED		NEPZ	SALINE	NOT_HOSTILE	8.3	1	RAJ_4229	Triticum_aestivum	7	81	88	67	72	130	34.9	58	2.2	2.0	1200	40.9
152	2014	2014		KUMARGANJ-RECLAIMED	KUMARGANJ-RECLAIMED		NEPZ	SALINE	NOT_HOSTILE	8.3	1	DBW_14	Triticum_aestivum	8	80	84	60	62	129	38.7	38	1.5	1.2	700	43.9
153	2014	2014		KUMARGANJ-RECLAIMED	KUMARGANJ-RECLAIMED		NEPZ	SALINE	NOT_HOSTILE	8.3	1	DBW_46	Triticum_aestivum	9	86	91	80	50	133	35.2	66	2.5	2.2	1400	38.6
154	2014	2014		KUMARGANJ-RECLAIMED	KUMARGANJ-RECLAIMED		NEPZ	SALINE	NOT_HOSTILE	8.3	1	KHARCHIA_65	Triticum_aestivum	10	84	88	103	88	130	36.4	45	1.7	2.0	1400	35.9
155	2014	2014		KUMARGANJ-RECLAIMED	KUMARGANJ-RECLAIMED		NEPZ	SALINE	NOT_HOSTILE	8.3	1	DBW_16	Triticum_aestivum	11	87	93	71	78	132	37.6	53	2.0	2.4	1450	41.0
156	2014	2014		KUMARGANJ-RECLAIMED	KUMARGANJ-RECLAIMED		NEPZ	SALINE	NOT_HOSTILE	8.3	1	HI_8498	Triticum_turgidum	12	88	91	74	64	135	37.6	56	2.2	1.5	1000	38.0
157	2014	2014		KUMARGANJ-RECLAIMED	KUMARGANJ-RECLAIMED		NEPZ	SALINE	NOT_HOSTILE	8.3	1	MACS_6222	Triticum_aestivum	13	91	99	77	68	133	33	65	2.2	1.4	800	44.3
158	2014	2014		KUMARGANJ-RECLAIMED	KUMARGANJ-RECLAIMED		NEPZ	SALINE	NOT_HOSTILE	8.3	1	DBW_71	Triticum_aestivum	14	81	86	72	60	129	34.8	50	1.4	1.9	1150	42.1
159	2014	2014		KUMARGANJ-RECLAIMED	KUMARGANJ-RECLAIMED		NEPZ	SALINE	NOT_HOSTILE	8.3	1	NW_4018	Triticum_aestivum	15	89	96	82	53	128	28.8	67	2.0	1.5	950	39.8
160	2014	2014		KUMARGANJ-RECLAIMED	KUMARGANJ-RECLAIMED		NEPZ	SALINE	NOT_HOSTILE	8.3	1	NW_4092	Triticum_aestivum	16	88	94	79	48	131	32.3	45	2.1	1.6	1050	37.4
161	2014	2014		KUMARGANJ-RECLAIMED	KUMARGANJ-RECLAIMED		NEPZ	SALINE	NOT_HOSTILE	8.3	1	WH_1021	Triticum_aestivum	17	83	88	74	69	130	29.2	50	1.5	1.4	830	41.7
162	2014	2014		KUMARGANJ-RECLAIMED	KUMARGANJ-RECLAIMED		NEPZ	SALINE	NOT_HOSTILE	8.3	1	KRL_19	Triticum_aestivum	18	80	87	71	59	128	29.6	74	2.3	1.4	925	37.0
163	2014	2014		KUMARGANJ-RECLAIMED	KUMARGANJ-RECLAIMED		NEPZ	SALINE	NOT_HOSTILE	8.3	1	DBW_51	Triticum_aestivum	19	88	94	77	73	128	34	53	1.8	1.3	775	41.7
164	2014	2014		KUMARGANJ-RECLAIMED	KUMARGANJ-RECLAIMED		NEPZ	SALINE	NOT_HOSTILE	8.3	1	HI_1563	Triticum_aestivum	20	81	86	59	59	126	33.2	54	1.5	1.5	950	39.6
165	2014	2014		KUMARGANJ-RECLAIMED	KUMARGANJ-RECLAIMED		NEPZ	SALINE	NOT_HOSTILE	8.3	1	CBW_38	Triticum_aestivum	21	86	91	73	86	128	33.7	49	1.6	2.2	1350	41.5
166	2014	2014		KUMARGANJ-RECLAIMED	KUMARGANJ-RECLAIMED		NEPZ	SALINE	NOT_HOSTILE	8.3	1	GW_322	Triticum_aestivum	22	83	83	69	61	128	29.1	58	1.7	1.2	900	34.7
167	2014	2014		KUMARGANJ-RECLAIMED	KUMARGANJ-RECLAIMED		NEPZ	SALINE	NOT_HOSTILE	8.3	1	RW_3684	Triticum_aestivum	23	89	94	84	76	132	36.2	50	2.1	1.9	1350	36.1
168	2014	2014		KUMARGANJ-RECLAIMED	KUMARGANJ-RECLAIMED		NEPZ	SALINE	NOT_HOSTILE	8.3	1	KRL_3-4	Triticum_aestivum	24	84	91	100	72	131	36	41	1.5	1.9	1250	38.4
169	2014	2014		KUMARGANJ-RECLAIMED	KUMARGANJ-RECLAIMED		NEPZ	SALINE	NOT_HOSTILE	8.3	1	KRL_1-4	Triticum_aestivum	25	83	88	68	52	126	34.5	43	1.1	1.4	950	37.9
170	2014	2014		KUMARGANJ-RECLAIMED	KUMARGANJ-RECLAIMED		NEPZ	SALINE	NOT_HOSTILE	8.3	1	UP_262	Triticum_aestivum	26	82	87	76	67	128	37.9	51	1.9	1.9	1250	38.9
171	2014	2014		KUMARGANJ-RECLAIMED	KUMARGANJ-RECLAIMED		NEPZ	SALINE	NOT_HOSTILE	8.3	1	HD_2932	Triticum_aestivum	27	85	90	66	71	126	34.9	49	1.6	1.4	850	41.8
172	2014	2014		KUMARGANJ-RECLAIMED	KUMARGANJ-RECLAIMED		NEPZ	SALINE	NOT_HOSTILE	8.3	1	HW_2044	Triticum_aestivum	28	81	86	60	63	130	29.8	41	1.2	1.2	800	36.0
173	2014	2014		KUMARGANJ-RECLAIMED	KUMARGANJ-RECLAIMED		NEPZ	SALINE	NOT_HOSTILE	8.3	1	HD_2009	Triticum_aestivum	29	86	91	89	58	131	34.4	44	2.1	2.1	1250	42.6
174	2014	2014		KUMARGANJ-RECLAIMED	KUMARGANJ-RECLAIMED		NEPZ	SALINE	NOT_HOSTILE	8.3	1	HD_2733	Triticum_aestivum	30	88	92	70	62	130	33.2	47	1.7	1.5	900	40.3
175	2014	2014		KUMARGANJ-RECLAIMED	KUMARGANJ-RECLAIMED		NEPZ	SALINE	NOT_HOSTILE	8.3	1	DBW_39	Triticum_aestivum	31	85	90	72	75	129	38.6	52	2.1	2.4	1400	43.1
176	2014	2014		KUMARGANJ-RECLAIMED	KUMARGANJ-RECLAIMED		NEPZ	SALINE	NOT_HOSTILE	8.3	1	NW_1067	Triticum_aestivum	32	88	94	65	56	132	31	47	1.6	1.4	800	44.3
177	2014	2014		KUMARGANJ-RECLAIMED	KUMARGANJ-RECLAIMED		NEPZ	SALINE	NOT_HOSTILE	8.3	1	WH_1105	Triticum_aestivum	33	85	90	65	49	129	32.5	59	2.0	1.1	700	37.9
178	2014	2014		KUMARGANJ-RECLAIMED	KUMARGANJ-RECLAIMED		NEPZ	SALINE	NOT_HOSTILE	8.3	1	RAJ_4238	Triticum_aestivum	34	82	88	59	58	128	28.3	50	1.4	1.2	600	49.7
179	2014	2014		KUMARGANJ-RECLAIMED	KUMARGANJ-RECLAIMED		NEPZ	SALINE	NOT_HOSTILE	8.3	1	KRL_210	Triticum_aestivum	35	83	90	75	62	131	38.2	42	1.7	1.5	950	40.3
180	2014	2014		KUMARGANJ-RECLAIMED	KUMARGANJ-RECLAIMED		NEPZ	SALINE	NOT_HOSTILE	8.3	1	KRL_213	Triticum_aestivum	36	90	92	69	61	129	34.1	53	1.8	1.7	1100	37.5
181	2014	2014		KUMARGANJ-RECLAIMED	KUMARGANJ-RECLAIMED		NEPZ	SALINE	NOT_HOSTILE	8.3	2	KRL_3-4	Triticum_aestivum	37	85	90	98	89	131	38.3	38	1.2	2.0	1300	38.5
182	2014	2014		KUMARGANJ-RECLAIMED	KUMARGANJ-RECLAIMED		NEPZ	SALINE	NOT_HOSTILE	8.3	2	UP_262	Triticum_aestivum	38	84	90	78	69	128	38.2	50	1.9	1.7	1200	35.1
183	2014	2014		KUMARGANJ-RECLAIMED	KUMARGANJ-RECLAIMED		NEPZ	SALINE	NOT_HOSTILE	8.3	2	KHARCHIA_65	Triticum_aestivum	39	84	89	98	79	129	33.1	47	1.6	1.8	1300	35.5
184	2014	2014		KUMARGANJ-RECLAIMED	KUMARGANJ-RECLAIMED		NEPZ	SALINE	NOT_HOSTILE	8.3	2	DBW_17	Triticum_aestivum	40	82	88	63	76	127	35.6	57	2.0	2.7	1500	45.0
185	2014	2014		KUMARGANJ-RECLAIMED	KUMARGANJ-RECLAIMED		NEPZ	SALINE	NOT_HOSTILE	8.3	2	DBW_71	Triticum_aestivum	41	79	86	72	67	128	36.1	52	2.0	1.8	1100	41.7
186	2014	2014		KUMARGANJ-RECLAIMED	KUMARGANJ-RECLAIMED		NEPZ	SALINE	NOT_HOSTILE	8.3	2	DBW_39	Triticum_aestivum	42	83	88	73	73	129	38.5	50	1.8	2.5	1450	42.3
187	2014	2014		KUMARGANJ-RECLAIMED	KUMARGANJ-RECLAIMED		NEPZ	SALINE	NOT_HOSTILE	8.3	2	WH_1021	Triticum_aestivum	43	85	90	72	68	129	26.5	41	1.1	1.2	700	42.9
188	2014	2014		KUMARGANJ-RECLAIMED	KUMARGANJ-RECLAIMED		NEPZ	SALINE	NOT_HOSTILE	8.3	2	KRL_210	Triticum_aestivum	44	83	91	72	74	129	34.4	47	1.7	1.5	900	41.8
189	2014	2014		KUMARGANJ-RECLAIMED	KUMARGANJ-RECLAIMED		NEPZ	SALINE	NOT_HOSTILE	8.3	2	DBW_16	Triticum_aestivum	45	89	95	71	66	130	36.2	50	1.8	2.1	1350	39.2
190	2014	2014		KUMARGANJ-RECLAIMED	KUMARGANJ-RECLAIMED		NEPZ	SALINE	NOT_HOSTILE	8.3	2	HD_2009	Triticum_aestivum	46	88	92	85	69	130	33.4	45	1.9	2.1	1225	42.7
191	2014	2014		KUMARGANJ-RECLAIMED	KUMARGANJ-RECLAIMED		NEPZ	SALINE	NOT_HOSTILE	8.3	2	CBW_38	Triticum_aestivum	47	87	94	77	84	128	33.1	48	1.6	2.4	1400	42.4
192	2014	2014		KUMARGANJ-RECLAIMED	KUMARGANJ-RECLAIMED		NEPZ	SALINE	NOT_HOSTILE	8.3	2	DPW_621-50	Triticum_aestivum	48	85	91	78	65	129	36	60	1.8	3.1	1850	42.4
193	2014	2014		KUMARGANJ-RECLAIMED	KUMARGANJ-RECLAIMED		NEPZ	SALINE	NOT_HOSTILE	8.3	2	DBW_46	Triticum_aestivum	49	87	92	79	64	134	37.1	56	2.2	2.3	1450	39.5
194	2014	2014		KUMARGANJ-RECLAIMED	KUMARGANJ-RECLAIMED		NEPZ	SALINE	NOT_HOSTILE	8.3	2	RAJ_4238	Triticum_aestivum	50	83	89	60	59	130	29.4	47	1.2	1.2	650	46.9
195	2014	2014		KUMARGANJ-RECLAIMED	KUMARGANJ-RECLAIMED		NEPZ	SALINE	NOT_HOSTILE	8.3	2	NW_4092	Triticum_aestivum	51	90	95	79	50	129	30.3	55	1.8	1.5	1000	37.6
196	2014	2014		KUMARGANJ-RECLAIMED	KUMARGANJ-RECLAIMED		NEPZ	SALINE	NOT_HOSTILE	8.3	2	KRL_1-4	Triticum_aestivum	52	83	89	69	62	128	37.1	57	1.0	1.3	900	37.2
197	2014	2014		KUMARGANJ-RECLAIMED	KUMARGANJ-RECLAIMED		NEPZ	SALINE	NOT_HOSTILE	8.3	2	K_0307	Triticum_aestivum	53	86	92	87	66	129	38.8	50	2.0	2.0	1250	39.2
198	2014	2014		KUMARGANJ-RECLAIMED	KUMARGANJ-RECLAIMED		NEPZ	SALINE	NOT_HOSTILE	8.3	2	GW_322	Triticum_aestivum	54	85	90	72	56	128	30.6	54	1.7	1.2	800	38.0
199	2014	2014		KUMARGANJ-RECLAIMED	KUMARGANJ-RECLAIMED		NEPZ	SALINE	NOT_HOSTILE	8.3	2	DBW_51	Triticum_aestivum	55	89	94	79	65	130	30.9	55	1.7	1.4	800	43.4
200	2014	2014		KUMARGANJ-RECLAIMED	KUMARGANJ-RECLAIMED		NEPZ	SALINE	NOT_HOSTILE	8.3	2	PDW_314	Triticum_turgidum	56	91	96	64	77	131	38.3	54	1.8	2.7	1650	40.7
201	2014	2014		KUMARGANJ-RECLAIMED	KUMARGANJ-RECLAIMED		NEPZ	SALINE	NOT_HOSTILE	8.3	2	MACS_6222	Triticum_aestivum	57	91	99	75	52	131	36.4	53	1.1	1.5	850	42.9
202	2014	2014		KUMARGANJ-RECLAIMED	KUMARGANJ-RECLAIMED		NEPZ	SALINE	NOT_HOSTILE	8.3	2	HD_2932	Triticum_aestivum	58	86	91	70	73	126	36.3	47	1.7	1.5	950	39.6
203	2014	2014		KUMARGANJ-RECLAIMED	KUMARGANJ-RECLAIMED		NEPZ	SALINE	NOT_HOSTILE	8.3	2	KRL_213	Triticum_aestivum	59	88	93	70	75	129	35.7	45	1.7	1.8	1250	35.1
204	2014	2014		KUMARGANJ-RECLAIMED	KUMARGANJ-RECLAIMED		NEPZ	SALINE	NOT_HOSTILE	8.3	2	RAJ_4229	Triticum_aestivum	60	83	90	62	72	128	32.5	48	1.9	1.3	1000	31.9
205	2014	2014		KUMARGANJ-RECLAIMED	KUMARGANJ-RECLAIMED		NEPZ	SALINE	NOT_HOSTILE	8.3	2	WH_1105	Triticum_aestivum	61	86	92	70	54	128	29.8	63	1.9	1.0	650	39.4
206	2014	2014		KUMARGANJ-RECLAIMED	KUMARGANJ-RECLAIMED		NEPZ	SALINE	NOT_HOSTILE	8.3	2	HD_2967	Triticum_aestivum	62	88	93	72	75	129	35.6	67	2.2	2.5	1450	42.6
207	2014	2014		KUMARGANJ-RECLAIMED	KUMARGANJ-RECLAIMED		NEPZ	SALINE	NOT_HOSTILE	8.3	2	HD_2733	Triticum_aestivum	63	90	93	64	70	131	30.7	42	1.3	1.4	850	41.8
208	2014	2014		KUMARGANJ-RECLAIMED	KUMARGANJ-RECLAIMED		NEPZ	SALINE	NOT_HOSTILE	8.3	2	NW_4018	Triticum_aestivum	64	91	96	76	48	127	31.7	67	2.2	1.6	850	48.2
209	2014	2014		KUMARGANJ-RECLAIMED	KUMARGANJ-RECLAIMED		NEPZ	SALINE	NOT_HOSTILE	8.3	2	HI_1563	Triticum_aestivum	65	82	87	64	70	128	35.7	61	2.2	1.5	1000	37.2
210	2014	2014		KUMARGANJ-RECLAIMED	KUMARGANJ-RECLAIMED		NEPZ	SALINE	NOT_HOSTILE	8.3	2	DBW_14	Triticum_aestivum	66	82	87	57	51	127	38.5	48	1.5	1.4	750	46.0
211	2014	2014		KUMARGANJ-RECLAIMED	KUMARGANJ-RECLAIMED		NEPZ	SALINE	NOT_HOSTILE	8.3	2	HI_8498	Triticum_turgidum	67	90	92	69	57	133	35.8	51	1.9	1.3	950	33.6
212	2014	2014		KUMARGANJ-RECLAIMED	KUMARGANJ-RECLAIMED		NEPZ	SALINE	NOT_HOSTILE	8.3	2	BH_1146	Triticum_aestivum	68	86	90	107	77	130	38.4	35	1.4	1.5	1100	33.8
213	2014	2014		KUMARGANJ-RECLAIMED	KUMARGANJ-RECLAIMED		NEPZ	SALINE	NOT_HOSTILE	8.3	2	NW_1067	Triticum_aestivum	69	90	95	67	62	133	29.3	42	1.2	1.6	850	47.3
214	2014	2014		KUMARGANJ-RECLAIMED	KUMARGANJ-RECLAIMED		NEPZ	SALINE	NOT_HOSTILE	8.3	2	RW_3684	Triticum_aestivum	70	89	94	81	62	130	38.3	58	2.4	1.9	1300	37.2
215	2014	2014		KUMARGANJ-RECLAIMED	KUMARGANJ-RECLAIMED		NEPZ	SALINE	NOT_HOSTILE	8.3	2	KRL_19	Triticum_aestivum	71	83	89	69	68	126	31.9	67	2.0	1.2	900	33.3
216	2014	2014		KUMARGANJ-RECLAIMED	KUMARGANJ-RECLAIMED		NEPZ	SALINE	NOT_HOSTILE	8.3	2	HW_2044	Triticum_aestivum	72	80	86	65	51	129	33.6	47	1.3	1.2	850	35.3
217	2014	2014		KUMARGANJ-SODIC	KUMARGANJ-SODIC		NEPZ	SODIC	HOSTILE	9.5	1	PDW_314	Triticum_turgidum	1	89	95	52	34	130	20.8	36	0.9	0.6	350	44.0
218	2014	2014		KUMARGANJ-SODIC	KUMARGANJ-SODIC		NEPZ	SODIC	HOSTILE	9.5	1	HD_2967	Triticum_aestivum	2	86	92	57	44	129	28.1	35	1.1	0.8	550	38.2
219	2014	2014		KUMARGANJ-SODIC	KUMARGANJ-SODIC		NEPZ	SODIC	HOSTILE	9.5	1	DPW_621-50	Triticum_aestivum	3	86	92	66	55	127	28.4	46	1.5	1.7	1000	42.5
220	2014	2014		KUMARGANJ-SODIC	KUMARGANJ-SODIC		NEPZ	SODIC	HOSTILE	9.5	1	DBW_17	Triticum_aestivum	4	84	92	49	56	128	25.4	36	1.0	1.0	575	41.7
221	2014	2014		KUMARGANJ-SODIC	KUMARGANJ-SODIC		NEPZ	SODIC	HOSTILE	9.5	1	K_0307	Triticum_aestivum	5	88	92	64	58	128	32	41	1.2	1.1	700	40.7
222	2014	2014		KUMARGANJ-SODIC	KUMARGANJ-SODIC		NEPZ	SODIC	HOSTILE	9.5	1	BH_1146	Triticum_aestivum	6	88	94	73	60	128	27.5	33	0.8	0.9	575	38.3
223	2014	2014		KUMARGANJ-SODIC	KUMARGANJ-SODIC		NEPZ	SODIC	HOSTILE	9.5	1	RAJ_4229	Triticum_aestivum	7	83	90	52	65	124	28.3	37	1.0	0.7	475	37.9
224	2014	2014		KUMARGANJ-SODIC	KUMARGANJ-SODIC		NEPZ	SODIC	HOSTILE	9.5	1	DBW_14	Triticum_aestivum	8	76	82	48	44	126	29.2	26	1.4	0.7	500	36.0
225	2014	2014		KUMARGANJ-SODIC	KUMARGANJ-SODIC		NEPZ	SODIC	HOSTILE	9.5	1	DBW_46	Triticum_aestivum	9	87	93	70	40	130	32	49	1.6	1.0	625	41.6
226	2014	2014		KUMARGANJ-SODIC	KUMARGANJ-SODIC		NEPZ	SODIC	HOSTILE	9.5	1	KHARCHIA_65	Triticum_aestivum	10	83	90	70	54	128	29.8	41	1.3	1.0	650	40.0
227	2014	2014		KUMARGANJ-SODIC	KUMARGANJ-SODIC		NEPZ	SODIC	HOSTILE	9.5	1	DBW_16	Triticum_aestivum	11	88	91	58	48	131	28.8	35	0.7	1.1	700	40.7
228	2014	2014		KUMARGANJ-SODIC	KUMARGANJ-SODIC		NEPZ	SODIC	HOSTILE	9.5	1	HI_8498	Triticum_turgidum	12	90	95	60	54	128	33.2	34	1.1	0.7	450	37.8
229	2014	2014		KUMARGANJ-SODIC	KUMARGANJ-SODIC		NEPZ	SODIC	HOSTILE	9.5	1	MACS_6222	Triticum_aestivum	13	90	94	60	34	127	25.9	41	1.1	0.5	350	38.6
230	2014	2014		KUMARGANJ-SODIC	KUMARGANJ-SODIC		NEPZ	SODIC	HOSTILE	9.5	1	DBW_71	Triticum_aestivum	14	82	86	58	50	125	28.5	41	1.2	0.9	550	40.0
231	2014	2014		KUMARGANJ-SODIC	KUMARGANJ-SODIC		NEPZ	SODIC	HOSTILE	9.5	1	NW_4018	Triticum_aestivum	15	90	95	69	37	129	24	47	1.2	0.7	450	38.9
232	2014	2014		KUMARGANJ-SODIC	KUMARGANJ-SODIC		NEPZ	SODIC	HOSTILE	9.5	1	NW_4092	Triticum_aestivum	16	88	93	66	36	129	28	37	1.1	0.9	550	39.1
233	2014	2014		KUMARGANJ-SODIC	KUMARGANJ-SODIC		NEPZ	SODIC	HOSTILE	9.5	1	WH_1021	Triticum_aestivum	17	85	88	68	39	126	25.1	46	0.9	0.8	500	38.0
234	2014	2014		KUMARGANJ-SODIC	KUMARGANJ-SODIC		NEPZ	SODIC	HOSTILE	9.5	1	KRL_19	Triticum_aestivum	18	80	85	59	59	124	30.7	41	1.3	1.4	825	41.2
235	2014	2014		KUMARGANJ-SODIC	KUMARGANJ-SODIC		NEPZ	SODIC	HOSTILE	9.5	1	DBW_51	Triticum_aestivum	19	85	90	62	43	127	25.9	36	1.0	0.7	450	36.7
236	2014	2014		KUMARGANJ-SODIC	KUMARGANJ-SODIC		NEPZ	SODIC	HOSTILE	9.5	1	HI_1563	Triticum_aestivum	20	83	89	69	58	123	29.3	38	1.1	0.7	475	37.9
237	2014	2014		KUMARGANJ-SODIC	KUMARGANJ-SODIC		NEPZ	SODIC	HOSTILE	9.5	1	CBW_38	Triticum_aestivum	21	88	94	60	50	124	23.5	37	0.9	0.7	475	37.9
238	2014	2014		KUMARGANJ-SODIC	KUMARGANJ-SODIC		NEPZ	SODIC	HOSTILE	9.5	1	GW_322	Triticum_aestivum	22	82	88	57	44	126	28	39	1.0	0.7	450	41.1
239	2014	2014		KUMARGANJ-SODIC	KUMARGANJ-SODIC		NEPZ	SODIC	HOSTILE	9.5	1	RW_3684	Triticum_aestivum	23	80	94	61	50	129	23	39	0.9	0.6	425	37.6
240	2014	2014		KUMARGANJ-SODIC	KUMARGANJ-SODIC		NEPZ	SODIC	HOSTILE	9.5	1	KRL_3-4	Triticum_aestivum	24	82	89	78	66	128	28	25	0.7	0.7	400	41.3
241	2014	2014		KUMARGANJ-SODIC	KUMARGANJ-SODIC		NEPZ	SODIC	HOSTILE	9.5	1	KRL_1-4	Triticum_aestivum	25	78	85	65	51	124	28.1	38	1.2	0.7	500	34.0

Abbreviations	Description
DTH	Days to heading, days
DTA	Days to anthesis, days
PHT	Plant height at maturity, cm
PTM	Productive tillers per meter, number
DTM	Days to maturity, days
TGW	1000 grain weight, g
GNS	Grain number per spike, number
GWS	Grain weight per spike, g
GYD	Grain yield, t/ha
HI	Harvest index, %

Samples!	Year!	Year1!	Year2!	Site_Name!	Site2014!	site2015!	ZONE!	Soil!	HOSTILE_NOT!	SOIL_ pH!	Replication!	Genotype!	Species!	Plot_No!	DTH	DTA	PHT	PTM	DTM	TGW	GNS	GWS	GYD	BYD	HI		Abbreviations	Description
242	2014	2014		KUMARGANJ-SODIC	KUMARGANJ-SODIC		NEPZ	SODIC	HOSTILE	9.5	1	UP_262	Triticum_aestivum	26	81	86	72	39	126	18.3	34	1.0	0.8	500	40.0			
243	2014	2014		KUMARGANJ-SODIC	KUMARGANJ-SODIC		NEPZ	SODIC	HOSTILE	9.5	1	HD_2932	Triticum_aestivum	27	82	88	67	49	128	32.6	31	1.3	1.3	800	40.6			
244	2014	2014		KUMARGANJ-SODIC	KUMARGANJ-SODIC		NEPZ	SODIC	HOSTILE	9.5	1	HW_2044	Triticum_aestivum	28	80	86	60	60	125	34.8	48	1.7	1.1	600	45.8			
245	2014	2014		KUMARGANJ-SODIC	KUMARGANJ-SODIC		NEPZ	SODIC	HOSTILE	9.5	1	HD_2009	Triticum_aestivum	29	84	90	68	38	127	30.9	44	1.4	0.7	450	37.8			
246	2014	2014		KUMARGANJ-SODIC	KUMARGANJ-SODIC		NEPZ	SODIC	HOSTILE	9.5	1	HD_2733	Triticum_aestivum	30	88	94	57	66	131	28.6	28	0.8	0.7	500	36.0			
247	2014	2014		KUMARGANJ-SODIC	KUMARGANJ-SODIC		NEPZ	SODIC	HOSTILE	9.5	1	DBW_39	Triticum_aestivum	31	80	86	68	55	128	35.7	46	1.7	1.1	750	37.3			
248	2014	2014		KUMARGANJ-SODIC	KUMARGANJ-SODIC		NEPZ	SODIC	HOSTILE	9.5	1	NW_1067	Triticum_aestivum	32	85	90	70	54	130	35.3	49	1.1	1.7	1000	41.5			
249	2014	2014		KUMARGANJ-SODIC	KUMARGANJ-SODIC		NEPZ	SODIC	HOSTILE	9.5	1	WH_1105	Triticum_aestivum	33	84	88	59	39	129	30.4	61	1.9	0.8	500	40.0			
250	2014	2014		KUMARGANJ-SODIC	KUMARGANJ-SODIC		NEPZ	SODIC	HOSTILE	9.5	1	RAJ_4238	Triticum_aestivum	34	82	89	54	52	126	24.8	27	0.7	0.7	450	36.7			
251	2014	2014		KUMARGANJ-SODIC	KUMARGANJ-SODIC		NEPZ	SODIC	HOSTILE	9.5	1	KRL_210	Triticum_aestivum	35	82	88	58	57	125	28.8	45	1.7	1.1	675	39.3			
252	2014	2014		KUMARGANJ-SODIC	KUMARGANJ-SODIC		NEPZ	SODIC	HOSTILE	9.5	1	KRL_213	Triticum_aestivum	36	82	88	58	55	128	26.2	43	1.0	1.0	600	40.8			
253	2014	2014		KUMARGANJ-SODIC	KUMARGANJ-SODIC		NEPZ	SODIC	HOSTILE	9.5	2	KRL_3-4	Triticum_aestivum	37	81	87	84	71	130	32.9	35	1.1	0.8	625	32.0			
254	2014	2014		KUMARGANJ-SODIC	KUMARGANJ-SODIC		NEPZ	SODIC	HOSTILE	9.5	2	UP_262	Triticum_aestivum	38	79	85	67	37	126	20.5	38	0.8	0.9	525	44.8			
255	2014	2014		KUMARGANJ-SODIC	KUMARGANJ-SODIC		NEPZ	SODIC	HOSTILE	9.5	2	KHARCHIA_65	Triticum_aestivum	39	81	87	74	60	129	30.9	46	1.4	1.1	675	39.3			
256	2014	2014		KUMARGANJ-SODIC	KUMARGANJ-SODIC		NEPZ	SODIC	HOSTILE	9.5	2	DBW_17	Triticum_aestivum	40	83	90	51	61	125	26.4	45	1.2	1.0	625	41.6			
257	2014	2014		KUMARGANJ-SODIC	KUMARGANJ-SODIC		NEPZ	SODIC	HOSTILE	9.5	2	DBW_71	Triticum_aestivum	41	80	85	66	59	126	33.9	36	1.3	1.0	600	40.0			
258	2014	2014		KUMARGANJ-SODIC	KUMARGANJ-SODIC		NEPZ	SODIC	HOSTILE	9.5	2	DBW_39	Triticum_aestivum	42	83	89	70	53	127	34.3	42	1.5	1.1	725	38.6			
259	2014	2014		KUMARGANJ-SODIC	KUMARGANJ-SODIC		NEPZ	SODIC	HOSTILE	9.5	2	WH_1021	Triticum_aestivum	43	83	89	74	46	125	30.1	51	1.1	0.8	525	38.1			
260	2014	2014		KUMARGANJ-SODIC	KUMARGANJ-SODIC		NEPZ	SODIC	HOSTILE	9.5	2	KRL_210	Triticum_aestivum	44	80	86	65	61	124	32.6	51	1.7	1.2	700	42.9			
261	2014	2014		KUMARGANJ-SODIC	KUMARGANJ-SODIC		NEPZ	SODIC	HOSTILE	9.5	2	DBW_16	Triticum_aestivum	45	86	92	61	52	128	30.4	38	1.0	1.2	700	41.4			
262	2014	2014		KUMARGANJ-SODIC	KUMARGANJ-SODIC		NEPZ	SODIC	HOSTILE	9.5	2	HD_2009	Triticum_aestivum	46	83	90	71	46	128	27.6	36	1.5	0.6	400	35.5			
263	2014	2014		KUMARGANJ-SODIC	KUMARGANJ-SODIC		NEPZ	SODIC	HOSTILE	9.5	2	CBW_38	Triticum_aestivum	47	86	92	67	68	126	26.5	44	1.0	0.8	600	34.2			
264	2014	2014		KUMARGANJ-SODIC	KUMARGANJ-SODIC		NEPZ	SODIC	HOSTILE	9.5	2	DPW_621-50	Triticum_aestivum	48	84	91	68	57	125	30.8	54	1.7	1.7	925	44.9			
265	2014	2014		KUMARGANJ-SODIC	KUMARGANJ-SODIC		NEPZ	SODIC	HOSTILE	9.5	2	DBW_46	Triticum_aestivum	49	85	92	77	46	131	34.7	52	1.8	1.2	750	38.7			
266	2014	2014		KUMARGANJ-SODIC	KUMARGANJ-SODIC		NEPZ	SODIC	HOSTILE	9.5	2	RAJ_4238	Triticum_aestivum	50	80	87	59	59	124	25.7	28	0.9	0.8	475	40.0			
267	2014	2014		KUMARGANJ-SODIC	KUMARGANJ-SODIC		NEPZ	SODIC	HOSTILE	9.5	2	NW_4092	Triticum_aestivum	51	88	91	70	36	128	30.6	44	1.3	0.8	500	42.0			
268	2014	2014		KUMARGANJ-SODIC	KUMARGANJ-SODIC		NEPZ	SODIC	HOSTILE	9.5	2	KRL_1-4	Triticum_aestivum	52	79	86	63	45	125	25.7	40	1.0	0.7	500	35.0			
269	2014	2014		KUMARGANJ-SODIC	KUMARGANJ-SODIC		NEPZ	SODIC	HOSTILE	9.5	2	K_0307	Triticum_aestivum	53	88	90	74	64	128	32.8	45	1.5	1.1	750	36.0			
270	2014	2014		KUMARGANJ-SODIC	KUMARGANJ-SODIC		NEPZ	SODIC	HOSTILE	9.5	2	GW_322	Triticum_aestivum	54	81	88	61	50	128	32.4	44	1.2	0.9	475	45.3			
271	2014	2014		KUMARGANJ-SODIC	KUMARGANJ-SODIC		NEPZ	SODIC	HOSTILE	9.5	2	DBW_51	Triticum_aestivum	55	83	88	69	40	129	28	40	1.1	0.7	450	37.8			
272	2014	2014		KUMARGANJ-SODIC	KUMARGANJ-SODIC		NEPZ	SODIC	HOSTILE	9.5	2	PDW_314	Triticum_turgidum	56	88	94	59	45	128	24.8	33	0.8	0.6	325	44.6			
273	2014	2014		KUMARGANJ-SODIC	KUMARGANJ-SODIC		NEPZ	SODIC	HOSTILE	9.5	2	MACS_6222	Triticum_aestivum	57	87	93	57	35	125	29.2	44	1.3	0.6	400	37.5			
274	2014	2014		KUMARGANJ-SODIC	KUMARGANJ-SODIC		NEPZ	SODIC	HOSTILE	9.5	2	HD_2932	Triticum_aestivum	58	83	90	64	50	128	34.2	40	1.4	1.2	825	36.4			
275	2014	2014		KUMARGANJ-SODIC	KUMARGANJ-SODIC		NEPZ	SODIC	HOSTILE	9.5	2	KRL_213	Triticum_aestivum	59	84	90	63	49	129	28.2	47	1.2	1.0	750	32.0			
276	2014	2014		KUMARGANJ-SODIC	KUMARGANJ-SODIC		NEPZ	SODIC	HOSTILE	9.5	2	RAJ_4229	Triticum_aestivum	60	80	88	57	52	123	32.7	39	1.1	0.8	500	41.0			
277	2014	2014		KUMARGANJ-SODIC	KUMARGANJ-SODIC		NEPZ	SODIC	HOSTILE	9.5	2	WH_1105	Triticum_aestivum	61	85	90	60	35	127	28.2	61	1.5	0.8	475	43.2			
278	2014	2014		KUMARGANJ-SODIC	KUMARGANJ-SODIC		NEPZ	SODIC	HOSTILE	9.5	2	HD_2967	Triticum_aestivum	62	85	93	61	45	129	29.7	43	1.3	0.8	500	40.0			
279	2014	2014		KUMARGANJ-SODIC	KUMARGANJ-SODIC		NEPZ	SODIC	HOSTILE	9.5	2	HD_2733	Triticum_aestivum	63	86	92	51	51	129	26.4	25	0.7	0.7	450	38.9			
280	2014	2014		KUMARGANJ-SODIC	KUMARGANJ-SODIC		NEPZ	SODIC	HOSTILE	9.5	2	NW_4018	Triticum_aestivum	64	88	93	72	49	127	29.1	52	1.4	0.8	500	40.0			
281	2014	2014		KUMARGANJ-SODIC	KUMARGANJ-SODIC		NEPZ	SODIC	HOSTILE	9.5	2	HI_1563	Triticum_aestivum	65	80	88	70	52	125	32.2	43	1.2	0.8	525	38.1			
282	2014	2014		KUMARGANJ-SODIC	KUMARGANJ-SODIC		NEPZ	SODIC	HOSTILE	9.5	2	DBW_14	Triticum_aestivum	66	78	84	56	54	125	30.7	30	1.2	0.8	600	33.3			
283	2014	2014		KUMARGANJ-SODIC	KUMARGANJ-SODIC		NEPZ	SODIC	HOSTILE	9.5	2	HI_8498	Triticum_turgidum	67	88	93	60	49	128	32.3	33	1.0	0.8	475	42.1			
284	2014	2014		KUMARGANJ-SODIC	KUMARGANJ-SODIC		NEPZ	SODIC	HOSTILE	9.5	2	BH_1146	Triticum_aestivum	68	90	96	73	73	127	26.2	31	0.8	0.8	500	41.0			
285	2014	2014		KUMARGANJ-SODIC	KUMARGANJ-SODIC		NEPZ	SODIC	HOSTILE	9.5	2	NW_1067	Triticum_aestivum	69	88	93	63	53	131	29.7	46	1.2	1.4	900	38.9			
286	2014	2014		KUMARGANJ-SODIC	KUMARGANJ-SODIC		NEPZ	SODIC	HOSTILE	9.5	2	RW_3684	Triticum_aestivum	70	88	93	66	46	129	28.7	42	1.0	0.8	500	38.0			
287	2014	2014		KUMARGANJ-SODIC	KUMARGANJ-SODIC		NEPZ	SODIC	HOSTILE	9.5	2	KRL_19	Triticum_aestivum	71	78	84	62	51	123	29.3	43	1.3	1.2	900	32.8			
288	2014	2014		KUMARGANJ-SODIC	KUMARGANJ-SODIC		NEPZ	SODIC	HOSTILE	9.5	2	HW_2044	Triticum_aestivum	72	78	85	64	77	124	38	38	1.4	1.2	625	49.1			
289	2014	2014		PUNDIBARI	PUNDIBARI		NEPZ	ACIDIC	HOSTILE	5.3	1	RW_3684	Triticum_aestivum	1	76	83	118	54	128	41.0	41	1.7	3.0	1950	38.7			
290	2014	2014		PUNDIBARI	PUNDIBARI		NEPZ	ACIDIC	HOSTILE	5.3	1	DBW_14	Triticum_aestivum	2	61	73	104	50	118	42.0	52	1.8	3.6	1800	50.6			
291	2014	2014		PUNDIBARI	PUNDIBARI		NEPZ	ACIDIC	HOSTILE	5.3	1	KRL_1-4	Triticum_aestivum	3	62	71	118	54	126	40.0	43	1.6	4.1	2050	50.1			
292	2014	2014		PUNDIBARI	PUNDIBARI		NEPZ	ACIDIC	HOSTILE	5.3	1	HW_2044	Triticum_aestivum	4	60	67	107	48	122	39.0	33	1.5	3.4	1750	48.2			
293	2014	2014		PUNDIBARI	PUNDIBARI		NEPZ	ACIDIC	HOSTILE	5.3	1	PDW_314	Triticum_turgidum	5	74	85	117	45	127	44.0	39	1.7	1.7	1450	29.9			
294	2014	2014		PUNDIBARI	PUNDIBARI		NEPZ	ACIDIC	HOSTILE	5.3	1	KRL_213	Triticum_aestivum	6	72	77	119	45	128	44.0	62	3.4	3.0	1950	38.2			
295	2014	2014		PUNDIBARI	PUNDIBARI		NEPZ	ACIDIC	HOSTILE	5.3	1	HI_8498	Triticum_turgidum	7	70	78	120	44	129	42.0	30	1.7	2.6	1550	41.9			
296	2014	2014		PUNDIBARI	PUNDIBARI		NEPZ	ACIDIC	HOSTILE	5.3	1	GW_322	Triticum_aestivum	8	70	77	121	56	129	36.0	48	1.7	3.5	1950	45.4			
297	2014	2014		PUNDIBARI	PUNDIBARI		NEPZ	ACIDIC	HOSTILE	5.3	1	DBW_71	Triticum_aestivum	9	66	74	122	45	130	38.0	46	2.2	3.2	1850	42.7			
298	2014	2014		PUNDIBARI	PUNDIBARI		NEPZ	ACIDIC	HOSTILE	5.3	1	WH_1105	Triticum_aestivum	10	71	76	122	48	130	37.0	64	2.4	3.0	1700	44.5			
299	2014	2014		PUNDIBARI	PUNDIBARI		NEPZ	ACIDIC	HOSTILE	5.3	1	RAJ_4229	Triticum_aestivum	11	63	72	120	52	128	42.0	51	1.8	3.4	1800	46.8			
300	2014	2014		PUNDIBARI	PUNDIBARI		NEPZ	ACIDIC	HOSTILE	5.3	1	BH_1146	Triticum_aestivum	12	66	74	124	50	131	43.0	40	1.8	2.8	2350	29.3			
301	2014	2014		PUNDIBARI	PUNDIBARI		NEPZ	ACIDIC	HOSTILE	5.3	1	HD_2009	Triticum_aestivum	13	64	69	122	42	129	43.0	70	3.0	3.3	1850	44.7			
302	2014	2014		PUNDIBARI	PUNDIBARI		NEPZ	ACIDIC	HOSTILE	5.3	1	DBW_17	Triticum_aestivum	14	75	82	130	46	137	44.0	34	1.6	3.7	2300	39.7			
303	2014	2014		PUNDIBARI	PUNDIBARI		NEPZ	ACIDIC	HOSTILE	5.3	1	WH_1021	Triticum_aestivum	15	68	75	120	44	127	44.0	37	1.6	3.3	1950	41.8			
304	2014	2014		PUNDIBARI	PUNDIBARI		NEPZ	ACIDIC	HOSTILE	5.3	1	UP_262	Triticum_aestivum	16	68	74	120	46	128	35.0	42	2.1	3.6	2400	37.3			
305	2014	2014		PUNDIBARI	PUNDIBARI		NEPZ	ACIDIC	HOSTILE	5.3	1	HD_2967	Triticum_aestivum	17	74	79	123	54	130	43.0	54	2.3	4.3	2350	45.4			
306	2014	2014		PUNDIBARI	PUNDIBARI		NEPZ	ACIDIC	HOSTILE	5.3	1	DBW_16	Triticum_aestivum	18	80	87	124	56	130	40.0	34	1.4	3.2	2250	35.0			
307	2014	2014		PUNDIBARI	PUNDIBARI		NEPZ	ACIDIC	HOSTILE	5.3	1	NW_1067	Triticum_aestivum	19	69	78	95	43	117	35.0	61	2.1	2.8	1850	37.9			
308	2014	2014		PUNDIBARI	PUNDIBARI		NEPZ	ACIDIC	HOSTILE	5.3	1	HI_1563	Triticum_aestivum	20	61	73	103	42	118	44.0	32	1.5	3.3	2150	38.6			
309	2014	2014		PUNDIBARI	PUNDIBARI		NEPZ	ACIDIC	HOSTILE	5.3	1	KRL_3-4	Triticum_aestivum	21	73	79	121	50	128	34.0	43	1.5	2.4	1670	36.1			
310	2014	2014		PUNDIBARI	PUNDIBARI		NEPZ	ACIDIC	HOSTILE	5.3	1	DBW_46	Triticum_aestivum	22	71	79	121	48	128	46.0	42	1.9	3.6	2220	41.0			
311	2014	2014		PUNDIBARI	PUNDIBARI		NEPZ	ACIDIC	HOSTILE	5.3	1	NW_4092	Triticum_aestivum	23	77	85	119	52	127	38.0	36	1.3	3.1	1950	39.5			
312	2014	2014		PUNDIBARI	PUNDIBARI		NEPZ	ACIDIC	HOSTILE	5.3	1	HD_2733	Triticum_aestivum	24	73	82	117	50	126	44.0	39	1.7	4.5	2550	44.4			
313	2014	2014		PUNDIBARI	PUNDIBARI		NEPZ	ACIDIC	HOSTILE	5.3	1	KRL_210	Triticum_aestivum	25	66	74	115	58	125	41.0	41	1.7	4.4	2320	46.9			
314	2014	2014		PUNDIBARI	PUNDIBARI		NEPZ	ACIDIC	HOSTILE	5.3	1	NW_4018	Triticum_aestivum	26	76	83	123	62	130	35.0	31	1.1	3.4	1870	44.9			
315	2014	2014		PUNDIBARI	PUNDIBARI		NEPZ	ACIDIC	HOSTILE	5.3	1	RAJ_4238	Triticum_aestivum	27	65	74	111	50	120	44.0	43	3.3	3.6	1950	45.8			
316	2014	2014		PUNDIBARI	PUNDIBARI		NEPZ	ACIDIC	HOSTILE	5.3	1	KHARCHIA_65	Triticum_aestivum	28	69	76	119	54	128	41.0	36	1.5	1.7	1750	24.3			
317	2014	2014		PUNDIBARI	PUNDIBARI		NEPZ	ACIDIC	HOSTILE	5.3	1	CBW_38	Triticum_aestivum	29	74	82	121	56	128	41.0	50	2.0	3.3	2050	40.7			
318	2014	2014		PUNDIBARI	PUNDIBARI		NEPZ	ACIDIC	HOSTILE	5.3	1	KRL_19	Triticum_aestivum	30	65	74	117	56	125	33.0	58	1.9	3.4	1850	45.4			
319	2014	2014		PUNDIBARI	PUNDIBARI		NEPZ	ACIDIC	HOSTILE	5.3	1	DBW_51	Triticum_aestivum	31	75	83	98	62	114	40.0	45	1.8	3.1	2100	36.9			
320	2014	2014		PUNDIBARI	PUNDIBARI		NEPZ	ACIDIC	HOSTILE	5.3	1	K_0307	Triticum_aestivum	32	69	76	115	52	125	43.0	58	2.5	4.1	2500	41.1			
321	2014	2014		PUNDIBARI	PUNDIBARI		NEPZ	ACIDIC	HOSTILE	5.3	1	DPW_621-50	Triticum_aestivum	33	74	80	121	56	128	36.0	50	1.6	3.5	2150	41.0			
322	2014	2014		PUNDIBARI	PUNDIBARI		NEPZ	ACIDIC	HOSTILE	5.3	1	HD_2932	Triticum_aestivum	34	67	75	122	64	129	36.0	29	1.0	4.0	2350	42.7			
323	2014	2014		PUNDIBARI	PUNDIBARI		NEPZ	ACIDIC	HOSTILE	5.3	1	MACS_6222	Triticum_aestivum	35	71	78	127	70	134	39.0	66	3.3	3.8	2050	46.4			
324	2014	2014		PUNDIBARI	PUNDIBARI		NEPZ	ACIDIC	HOSTILE	5.3	1	DBW_39	Triticum_aestivum	36	69	80	118	60	127	42.0	72	3.0	3.8	2200	43.3			
325	2014	2014		PUNDIBARI	PUNDIBARI		NEPZ	ACIDIC	HOSTILE	5.3	2	MACS_6222	Triticum_aestivum	37	70	78	127	44	134	36.0	34	1.2	3.7	2250	41.1			
326	2014	2014		PUNDIBARI	PUNDIBARI		NEPZ	ACIDIC	HOSTILE	5.3	2	WH_1021	Triticum_aestivum	38	66	75	120	52	127	43.0	54	2.3	3.7	2100	44.0			
327	2014	2014		PUNDIBARI	PUNDIBARI		NEPZ	ACIDIC	HOSTILE	5.3	2	HD_2733	Triticum_aestivum	39	76	82	117	50	126	40.0	34	1.4	3.9	2310	42.4			
328	2014	2014		PUNDIBARI	PUNDIBARI		NEPZ	ACIDIC	HOSTILE	5.3	2	HW_2044	Triticum_aestivum	40	58	67	107	56	122	35.0	30	1.1	3.4	1700	50.1			
329	2014	2014		PUNDIBARI	PUNDIBARI		NEPZ	ACIDIC	HOSTILE	5.3	2	BH_1146	Triticum_aestivum	41	64	74	124	52	131	41.0	32	1.3	2.8	2500	28.4			
330	2014	2014		PUNDIBARI	PUNDIBARI		NEPZ	ACIDIC	HOSTILE	5.3	2	KRL_19	Triticum_aestivum	42	67	74	117	58	125	35.0	34	1.4	2.4	2150	28.3			
331	2014	2014		PUNDIBARI	PUNDIBARI		NEPZ	ACIDIC	HOSTILE	5.3	2	DBW_71	Triticum_aestivum	43	68	74	122	56	130	39.0	35	1.4	4.4	2450	45.2			
332	2014	2014		PUNDIBARI	PUNDIBARI		NEPZ	ACIDIC	HOSTILE	5.3	2	DPW_621-50	Triticum_aestivum	44	72	80	121	60	128	37.0	57	2.1	3.8	2150	44.5			
333	2014	2014		PUNDIBARI	PUNDIBARI		NEPZ	ACIDIC	HOSTILE	5.3	2	HD_2967	Triticum_aestivum	45	76	79	123	48	130	42.0	48	2.0	4.2	2450	43.1			
334	2014	2014		PUNDIBARI	PUNDIBARI		NEPZ	ACIDIC	HOSTILE	5.3	2	HI_1563	Triticum_aestivum	46	60	73	103	52	118	43.0	34	1.5	4.0	2250	44.9			
335	2014	2014		PUNDIBARI	PUNDIBARI		NEPZ	ACIDIC	HOSTILE	5.3	2	NW_4018	Triticum_aestivum	47	74	83	123	50	130	36.0	46	1.7	3.0	2150	34.4			
336	2014	2014		PUNDIBARI	PUNDIBARI		NEPZ	ACIDIC	HOSTILE	5.3	2	KRL_213	Triticum_aestivum	48	70	77	119	62	128	42.0	43	1.8	4.2	2180	47.6			
337	2014	2014		PUNDIBARI	PUNDIBARI		NEPZ	ACIDIC	HOSTILE	5.3	2	KHARCHIA_65	Triticum_aestivum	49	70	76	119	54	128	36.0	32	1.2	1.7	1850	23.1			
338	2014	2014		PUNDIBARI	PUNDIBARI		NEPZ	ACIDIC	HOSTILE	5.3	2	RW_3684	Triticum_aestivum	50	77	83	118	56	128	39.0	38	1.5	3.7	2500	36.5			
339	2014	2014		PUNDIBARI	PUNDIBARI		NEPZ	ACIDIC	HOSTILE	5.3	2	NW_4092	Triticum_aestivum	51	79	85	119	60	127	41.0	68	2.8	3.4	2250	37.6			
340	2014	2014		PUNDIBARI	PUNDIBARI		NEPZ	ACIDIC	HOSTILE	5.3	2	GW_322	Triticum_aestivum	52	72	77	121	52	129	40.0	60	2.4	3.5	2000	43.3			
341	2014	2014		PUNDIBARI	PUNDIBARI		NEPZ	ACIDIC	HOSTILE	5.3	2	UP_262	Triticum_aestivum	53	67	74	120	56	128	34.0	47	1.6	4.2	2400	43.3			
342	2014	2014		PUNDIBARI	PUNDIBARI		NEPZ	ACIDIC	HOSTILE	5.3	2	DBW_51	Triticum_aestivum	54	73	83	98	64	114	42.0	48	2.4	3.2	2100	37.7			
343	2014	2014		PUNDIBARI	PUNDIBARI		NEPZ	ACIDIC	HOSTILE	5.3	2	RAJ_4238	Triticum_aestivum	55	67	74	111	56	120	43.0	42	1.8	3.7	2150	43.3			
344	2014	2014		PUNDIBARI	PUNDIBARI		NEPZ	ACIDIC	HOSTILE	5.3	2	DBW_17	Triticum_aestivum	56	76	82	130	54	137	40.0	48	1.9	3.8	2140	44.8			
345	2014	2014		PUNDIBARI	PUNDIBARI		NEPZ	ACIDIC	HOSTILE	5.3	2	KRL_1-4	Triticum_aestivum	57	64	71	118	54	126	46.0	34	1.6	4.0	2150	46.7			
346	2014	2014		PUNDIBARI	PUNDIBARI		NEPZ	ACIDIC	HOSTILE	5.3	2	WH_1105	Triticum_aestivum	58	70	76	122	52	130	41.0	43	1.8	3.2	1800	44.0			
347	2014	2014		PUNDIBARI	PUNDIBARI		NEPZ	ACIDIC	HOSTILE	5.3	2	NW_1067	Triticum_aestivum	59	67	78	95	58	117	40.0	45	1.8	3.3	2000	41.2			
348	2014	2014		PUNDIBARI	PUNDIBARI		NEPZ	ACIDIC	HOSTILE	5.3	2	DBW_39	Triticum_aestivum	60	70	80	118	58	127	43.0	47	2.3	4.1	2200	46.0			
349	2014	2014		PUNDIBARI	PUNDIBARI		NEPZ	ACIDIC	HOSTILE	5.3	2	HD_2009	Triticum_aestivum	61	65	69	122	42	129	41.0	43	1.8	2.4	1650	36.4			
350	2014	2014		PUNDIBARI	PUNDIBARI		NEPZ	ACIDIC	HOSTILE	5.3	2	K_0307	Triticum_aestivum	62	71	76	115	54	125	40.0	34	1.4	4.1	2300	44.6			
351	2014	2014		PUNDIBARI	PUNDIBARI		NEPZ	ACIDIC	HOSTILE	5.3	2	KRL_3-4	Triticum_aestivum	63	71	79	121	50	128	39.0	32	1.3	2.3	2250	25.7			
352	2014	2014		PUNDIBARI	PUNDIBARI		NEPZ	ACIDIC	HOSTILE	5.3	2	PDW_314	Triticum_turgidum	64	76	85	117	48	127	47.0	27	1.5	3.6	1950	45.6			
353	2014	2014		PUNDIBARI	PUNDIBARI		NEPZ	ACIDIC	HOSTILE	5.3	2	KRL_210	Triticum_aestivum	65	68	74	115	56	125	44.0	32	1.4	3.9	2450	39.7			
354	2014	2014		PUNDIBARI	PUNDIBARI		NEPZ	ACIDIC	HOSTILE	5.3	2	HI_8498	Triticum_turgidum	66	68	78	120	56	129	42.0	52	2.2	3.9	1780	55.2			
355	2014	2014		PUNDIBARI	PUNDIBARI		NEPZ	ACIDIC	HOSTILE	5.3	2	DBW_46	Triticum_aestivum	67	72	79	121	54	128	44.0	46	1.9	2.3	2450	23.8			
356	2014	2014		PUNDIBARI	PUNDIBARI		NEPZ	ACIDIC	HOSTILE	5.3	2	RAJ_4229	Triticum_aestivum	68	65	72	120	56	128	44.0	44	1.9	3.3	1800	46.1			
357	2014	2014		PUNDIBARI	PUNDIBARI		NEPZ	ACIDIC	HOSTILE	5.3	2	CBW_38	Triticum_aestivum	69	75	82	121	64	128	43.0	43	1.9	3.3	2300	35.5			
358	2014	2014		PUNDIBARI	PUNDIBARI		NEPZ	ACIDIC	HOSTILE	5.3	2	DBW_14	Triticum_aestivum	70	60	73	104	60	118	41.0	38	1.6	3.9	1820	53.5			
359	2014	2014		PUNDIBARI	PUNDIBARI		NEPZ	ACIDIC	HOSTILE	5.3	2	HD_2932	Triticum_aestivum	71	65	75	122	46	129	43.0	36	1.6	3.5	1800	48.3			
360	2014	2014		PUNDIBARI	PUNDIBARI		NEPZ	ACIDIC	HOSTILE	5.3	2	DBW_16	Triticum_aestivum	72	82	87	124	52	130	43.0	36	1.8	4.1	2100	49.1			
361	2014	2014		MALDA	MALDA		NEPZ	NORMAL	NOT_HOSTILE	7.7	1	RW_3684	Triticum_aestivum	1	69	78	121		113	38.7	51	2.0	3.0	2160	34.3			
362	2014	2014		MALDA	MALDA		NEPZ	NORMAL	NOT_HOSTILE	7.7	1	DBW_14	Triticum_aestivum	2	58	72	94		105	39.1	45	1.8	3.2	2105	38.0			
363	2014	2014		MALDA	MALDA		NEPZ	NORMAL	NOT_HOSTILE	7.7	1	KRL_1-4	Triticum_aestivum	3	62	71	110		112	51.3	39	2.0	3.5	2125	40.9			
364	2014	2014		MALDA	MALDA		NEPZ	NORMAL	NOT_HOSTILE	7.7	1	HW_2044	Triticum_aestivum	4	60	71	113		108	60.2	27	1.7	3.4	2965	28.5			
365	2014	2014		MALDA	MALDA		NEPZ	NORMAL	NOT_HOSTILE	7.7	1	PDW_314	Triticum_turgidum	5	67	74	107		114	45.3	41	1.8	3.6	2285	38.9			
366	2014	2014		MALDA	MALDA		NEPZ	NORMAL	NOT_HOSTILE	7.7	1	KRL_213	Triticum_aestivum	6	71	74	105		114	34.7	64	2.2	3.9	2105	46.1			
367	2014	2014		MALDA	MALDA		NEPZ	NORMAL	NOT_HOSTILE	7.7	1	HI_8498	Triticum_turgidum	7	60	71	107		112	50.8	48	2.4	3.2	2545	31.0			
368	2014	2014		MALDA	MALDA		NEPZ	NORMAL	NOT_HOSTILE	7.7	1	GW_322	Triticum_aestivum	8	67	80	116		112	34.4	49	1.7	3.2	2230	35.9			
369	2014	2014		MALDA	MALDA		NEPZ	NORMAL	NOT_HOSTILE	7.7	1	DBW_71	Triticum_aestivum	9	64	74	109		110	39.3	52	2.0	3.5	2095	41.5			
370	2014	2014		MALDA	MALDA		NEPZ	NORMAL	NOT_HOSTILE	7.7	1	WH_1105	Triticum_aestivum	10	65	77	108		112	36.6	60	2.2	2.6	1800	35.6			
371	2014	2014		MALDA	MALDA		NEPZ	NORMAL	NOT_HOSTILE	7.7	1	RAJ_4229	Triticum_aestivum	11	62	71	100		112	33.8	45	1.5	2.7	1730	38.4			
372	2014	2014		MALDA	MALDA		NEPZ	NORMAL	NOT_HOSTILE	7.7	1	BH_1146	Triticum_aestivum	12	63	71	167		109	41.7	45	1.9	2.6	1830	35.2			
373	2014	2014		MALDA	MALDA		NEPZ	NORMAL	NOT_HOSTILE	7.7	1	HD_2009	Triticum_aestivum	13	62	70	137		109	37.7	57	2.2	2.7	2285	29.5			
374	2014	2014		MALDA	MALDA		NEPZ	NORMAL	NOT_HOSTILE	7.7	1	DBW_17	Triticum_aestivum	14	71	80	102		115	38.4	47	1.8	2.9	1715	42.0			
375	2014	2014		MALDA	MALDA		NEPZ	NORMAL	NOT_HOSTILE	7.7	1	WH_1021	Triticum_aestivum	15	64	75	119		110	42.9	46	2.0	3.2	2030	39.4			
376	2014	2014		MALDA	MALDA		NEPZ	NORMAL	NOT_HOSTILE	7.7	1	UP_262	Triticum_aestivum	16	62	71	126		110	37.6	36	1.4	3.0	2120	35.4			
377	2014	2014		MALDA	MALDA		NEPZ	NORMAL	NOT_HOSTILE	7.7	1	HD_2967	Triticum_aestivum	17	71	80	114		115	42.1	55	2.3	3.0	1770	42.9			
378	2014	2014		MALDA	MALDA		NEPZ	NORMAL	NOT_HOSTILE	7.7	1	DBW_16	Triticum_aestivum	18	73	82	107		115	40.9	42	1.7	3.3	2050	40.2			
379	2014	2014		MALDA	MALDA		NEPZ	NORMAL	NOT_HOSTILE	7.7	1	NW_1067	Triticum_aestivum	19	65	75	98		112	33.7	49	1.6	3.0	2040	37.3			
380	2014	2014		MALDA	MALDA		NEPZ	NORMAL	NOT_HOSTILE	7.7	1	HI_1563	Triticum_aestivum	20	63	76	112		111	41.7	45	1.9	3.0	1925	39.5			
381	2014	2014		MALDA	MALDA		NEPZ	NORMAL	NOT_HOSTILE	7.7	1	KRL_3-4	Triticum_aestivum	21	69	79	151		113	39.7	34	1.3	1.2	1640	18.0			
382	2014	2014		MALDA	MALDA		NEPZ	NORMAL	NOT_HOSTILE	7.7	1	DBW_46	Triticum_aestivum	22	69	77	129		112	44.8	50	2.3	3.1	1950	39.2			
383	2014	2014		MALDA	MALDA		NEPZ	NORMAL	NOT_HOSTILE	7.7	1	NW_4092	Triticum_aestivum	23	71	80	116		115	38.0	57	2.2	2.7	1705	39.3			
384	2014	2014		MALDA	MALDA		NEPZ	NORMAL	NOT_HOSTILE	7.7	1	HD_2733	Triticum_aestivum	24	71	80	108		115	33.8	47	1.6	3.5	2140	40.7			
385	2014	2014		MALDA	MALDA		NEPZ	NORMAL	NOT_HOSTILE	7.7	1	KRL_210	Triticum_aestivum	25	63	71	112		108	39.2	50	2.0	3.6	2300	39.1			
386	2014	2014		MALDA	MALDA		NEPZ	NORMAL	NOT_HOSTILE	7.7	1	NW_4018	Triticum_aestivum	26	71	75	115		113	33.1	57	1.9	2.4	1545	38.2			
387	2014	2014		MALDA	MALDA		NEPZ	NORMAL	NOT_HOSTILE	7.7	1	RAJ_4238	Triticum_aestivum	27	62	71	100		108	37.1	44	1.6	2.4	1470	41.5			
388	2014	2014		MALDA	MALDA		NEPZ	NORMAL	NOT_HOSTILE	7.7	1	KHARCHIA_65	Triticum_aestivum	28	65	76	165		113	56.8	23	1.3	1.6	1700	23.2			
389	2014	2014		MALDA	MALDA		NEPZ	NORMAL	NOT_HOSTILE	7.7	1	CBW_38	Triticum_aestivum	29	70	79	120		112	37.0	39	1.4	3.1	1945	40.4			
390	2014	2014		MALDA	MALDA		NEPZ	NORMAL	NOT_HOSTILE	7.7	1	KRL_19	Triticum_aestivum	30	63	72	97		108	31.9	55	1.7	2.7	2060	33.0			
391	2014	2014		MALDA	MALDA		NEPZ	NORMAL	NOT_HOSTILE	7.7	1	DBW_51	Triticum_aestivum	31	69	79	115		115	37.5	64	2.4	3.5	2090	41.9			
392	2014	2014		MALDA	MALDA		NEPZ	NORMAL	NOT_HOSTILE	7.7	1	K_0307	Triticum_aestivum	32	69	79	122		115	40.9	48	2.0	3.5	1935	44.7			
393	2014	2014		MALDA	MALDA		NEPZ	NORMAL	NOT_HOSTILE	7.7	1	DPW_621-50	Triticum_aestivum	33	71	80	108		115	43.9	48	2.1	2.5	1900	33.2			
394	2014	2014		MALDA	MALDA		NEPZ	NORMAL	NOT_HOSTILE	7.7	1	HD_2932	Triticum_aestivum	34	64	74	105		112	36.7	41	1.5	3.8	2390	39.5			
395	2014	2014		MALDA	MALDA		NEPZ	NORMAL	NOT_HOSTILE	7.7	1	MACS_6222	Triticum_aestivum	35	69	79	109		114	35.1	61	2.1	2.8	2018	34.2			
396	2014	2014		MALDA	MALDA		NEPZ	NORMAL	NOT_HOSTILE	7.7	1	DBW_39	Triticum_aestivum	36	69	79	111		114	49.7	50	2.5	4.1	2350	43.2			
397	2014	2014		MALDA	MALDA		NEPZ	NORMAL	NOT_HOSTILE	7.7	2	MACS_6222	Triticum_aestivum	37	67	79	110		112	39.8	58	2.3	3.2	2125	37.9			
398	2014	2014		MALDA	MALDA		NEPZ	NORMAL	NOT_HOSTILE	7.7	2	WH_1021	Triticum_aestivum	38	63	74	112		108	46.0	42	1.9	3.5	2070	42.8			
399	2014	2014		MALDA	MALDA		NEPZ	NORMAL	NOT_HOSTILE	7.7	2	HD_2733	Triticum_aestivum	39	71	80	108		114	46.8	37	1.7	3.5	2050	42.4			
400	2014	2014		MALDA	MALDA		NEPZ	NORMAL	NOT_HOSTILE	7.7	2	HW_2044	Triticum_aestivum	40	60	69	112		108	40.0	51	2.0	2.8	1570	43.9			
401	2014	2014		MALDA	MALDA		NEPZ	NORMAL	NOT_HOSTILE	7.7	2	BH_1146	Triticum_aestivum	41	64	72	163		108	41.7	35	1.5	2.1	1500	35.3			
402	2014	2014		MALDA	MALDA		NEPZ	NORMAL	NOT_HOSTILE	7.7	2	KRL_19	Triticum_aestivum	42	63	74	109		110	29.4	69	2.0	2.2	1645	33.4			
403	2014	2014		MALDA	MALDA		NEPZ	NORMAL	NOT_HOSTILE	7.7	2	DBW_71	Triticum_aestivum	43	63	73	109		112	39.2	44	1.7	3.9	2255	43.0			
404	2014	2014		MALDA	MALDA		NEPZ	NORMAL	NOT_HOSTILE	7.7	2	DPW_621-50	Triticum_aestivum	44	69	79	107		115	40.8	47	1.9	2.5	1840	34.0			
405	2014	2014		MALDA	MALDA		NEPZ	NORMAL	NOT_HOSTILE	7.7	2	HD_2967	Triticum_aestivum	45	70	80	111		115	44.3	53	2.4	3.8	2200	43.2			
406	2014	2014		MALDA	MALDA		NEPZ	NORMAL	NOT_HOSTILE	7.7	2	HI_1563	Triticum_aestivum	46	60	72	110		109	45.0	48	2.2	3.7	2310	40.0			
407	2014	2014		MALDA	MALDA		NEPZ	NORMAL	NOT_HOSTILE	7.7	2	NW_4018	Triticum_aestivum	47	71	82	114		114	37.3	57	2.1	2.8	1830	38.3			
408	2014	2014		MALDA	MALDA		NEPZ	NORMAL	NOT_HOSTILE	7.7	2	KRL_213	Triticum_aestivum	48	71	82	110		114	36.0	54	2.0	3.9	2350	41.1			
409	2014	2014		MALDA	MALDA		NEPZ	NORMAL	NOT_HOSTILE	7.7	2	KHARCHIA_65	Triticum_aestivum	49	65	75	165		112	37.4	50	1.9	1.1	2050	13.2			
410	2014	2014		MALDA	MALDA		NEPZ	NORMAL	NOT_HOSTILE	7.7	2	RW_3684	Triticum_aestivum	50	71	81	116		114	35.7	51	1.8	2.7	1720	39.0			
411	2014	2014		MALDA	MALDA		NEPZ	NORMAL	NOT_HOSTILE	7.7	2	NW_4092	Triticum_aestivum	51	72	82	115		115	35.1	65	2.3	3.2	2125	37.2			
412	2014	2014		MALDA	MALDA		NEPZ	NORMAL	NOT_HOSTILE	7.7	2	GW_322	Triticum_aestivum	52	67	79	114		112	39.4	50	2.0	3.5	2215	39.1			
413	2014	2014		MALDA	MALDA		NEPZ	NORMAL	NOT_HOSTILE	7.7	2	UP_262	Triticum_aestivum	53	63	71	122		110	41.2	37	1.5	2.8	1850	37.8			
414	2014	2014		MALDA	MALDA		NEPZ	NORMAL	NOT_HOSTILE	7.7	2	DBW_51	Triticum_aestivum	54	72	81	114		114	37.9	61	2.3	3.5	2145	40.6			
415	2014	2014		MALDA	MALDA		NEPZ	NORMAL	NOT_HOSTILE	7.7	2	RAJ_4238	Triticum_aestivum	55	60	69	100		107	42.2	43	1.8	3.4	1965	42.7			
416	2014	2014		MALDA	MALDA		NEPZ	NORMAL	NOT_HOSTILE	7.7	2	DBW_17	Triticum_aestivum	56	70	79	98		114	37.9	51	1.9	3.3	1800	45.8			
417	2014	2014		MALDA	MALDA		NEPZ	NORMAL	NOT_HOSTILE	7.7	2	KRL_1-4	Triticum_aestivum	57	62	72	105		112	46.7	42	2.0	3.4	1940	43.3			
418	2014	2014		MALDA	MALDA		NEPZ	NORMAL	NOT_HOSTILE	7.7	2	WH_1105	Triticum_aestivum	58	64	76	105		110	35.4	66	2.3	3.2	1975	40.0			
419	2014	2014		MALDA	MALDA		NEPZ	NORMAL	NOT_HOSTILE	7.7	2	NW_1067	Triticum_aestivum	59	68	79	99		112	35.5	50	1.8	2.9	1745	41.3			
420	2014	2014		MALDA	MALDA		NEPZ	NORMAL	NOT_HOSTILE	7.7	2	DBW_39	Triticum_aestivum	60	71	80	113		114	49.6	34	1.7	3.8	2240	42.0			
421	2014	2014		MALDA	MALDA		NEPZ	NORMAL	NOT_HOSTILE	7.7	2	HD_2009	Triticum_aestivum	61	63	73	146		112	41.9	52	2.2	3.4	2440	34.8			
422	2014	2014		MALDA	MALDA		NEPZ	NORMAL	NOT_HOSTILE	7.7	2	K_0307	Triticum_aestivum	62	68	78	128		114	41.0	49	2.0	2.9	1810	40.1			
423	2014	2014		MALDA	MALDA		NEPZ	NORMAL	NOT_HOSTILE	7.7	2	KRL_3-4	Triticum_aestivum	63	68	76	149		114	33.9	42	1.4	1.5	1900	20.0			
424	2014	2014		MALDA	MALDA		NEPZ	NORMAL	NOT_HOSTILE	7.7	2	PDW_314	Triticum_turgidum	64	67	78	104		114	46.1	44	2.0	2.5	2030	30.5			
425	2014	2014		MALDA	MALDA		NEPZ	NORMAL	NOT_HOSTILE	7.7	2	KRL_210	Triticum_aestivum	65	65	77	117		112	32.9	65	2.1	3.3	2085	39.8			
426	2014	2014		MALDA	MALDA		NEPZ	NORMAL	NOT_HOSTILE	7.7	2	HI_8498	Triticum_turgidum	66	64	77	107		113	48.4	49	2.4	2.4	2220	27.3			
427	2014	2014		MALDA	MALDA		NEPZ	NORMAL	NOT_HOSTILE	7.7	2	DBW_46	Triticum_aestivum	67	65	79	129		113	45.1	45	2.0	4.1	2715	38.1			
428	2014	2014		MALDA	MALDA		NEPZ	NORMAL	NOT_HOSTILE	7.7	2	RAJ_4229	Triticum_aestivum	68	60	69	100		107	38.1	45	1.7	3.6	2165	41.6			
429	2014	2014		MALDA	MALDA		NEPZ	NORMAL	NOT_HOSTILE	7.7	2	CBW_38	Triticum_aestivum	69	69	79	120		112	39.3	44	1.7	3.7	2255	40.8			
430	2014	2014		MALDA	MALDA		NEPZ	NORMAL	NOT_HOSTILE	7.7	2	DBW_14	Triticum_aestivum	70	60	72	93		106	44.6	39	1.8	3.2	2145	36.8			
431	2014	2014		MALDA	MALDA		NEPZ	NORMAL	NOT_HOSTILE	7.7	2	HD_2932	Triticum_aestivum	71	65	75	106		112	40.1	41	1.6	3.2	2070	38.6			
432	2014	2014		MALDA	MALDA		NEPZ	NORMAL	NOT_HOSTILE	7.7	2	DBW_16	Triticum_aestivum	72	75	60	102		116	37.8	41	1.6	3.4	2441	35.2			
433	2015		2015	KARNAL		KARNAL	NWPZ	NORMAL	NOT_HOSTILE	7.5	1	WH_1105	Triticum_aestivum	1	89	96	85	90	132	37.5	77	2.9	5.9	3500	42.2			
434	2015		2015	KARNAL		KARNAL	NWPZ	NORMAL	NOT_HOSTILE	7.5	1	NW_4092	Triticum_aestivum	2	94	101	84	89	139	45.7	48	2.2	5.9	5100	28.7			
435	2015		2015	KARNAL		KARNAL	NWPZ	NORMAL	NOT_HOSTILE	7.5	1	DBW_16	Triticum_aestivum	3	91	98	76	111	136	40.9	51	2.1	5.2	4600	28.0			
436	2015		2015	KARNAL		KARNAL	NWPZ	NORMAL	NOT_HOSTILE	7.5	1	NW_4018	Triticum_aestivum	4	92	98	95	88	134	47.2	65	3.1	5.9	3900	37.5			
437	2015		2015	KARNAL		KARNAL	NWPZ	NORMAL	NOT_HOSTILE	7.5	1	HD_2733	Triticum_aestivum	5	90	97	81	101	133	55.3	53	2.9	4.1	3400	30.2			
438	2015		2015	KARNAL		KARNAL	NWPZ	NORMAL	NOT_HOSTILE	7.5	1	DBW_39	Triticum_aestivum	6	88	93	84	118	132	46.7	52	2.4	6.8	4300	39.5			
439	2015		2015	KARNAL		KARNAL	NWPZ	NORMAL	NOT_HOSTILE	7.5	1	CBW_38	Triticum_aestivum	7	88	95	87	77	131	42.6	49	2.1	6.9	4600	37.5			
440	2015		2015	KARNAL		KARNAL	NWPZ	NORMAL	NOT_HOSTILE	7.5	1	NW_1067	Triticum_aestivum	8	89	97	83	100	132	34.4	50	1.7	6.2	4200	37.2			
441	2015		2015	KARNAL		KARNAL	NWPZ	NORMAL	NOT_HOSTILE	7.5	1	DBW_51	Triticum_aestivum	9	87	94	83	87	130	44.0	51	2.3	5.7	4000	35.8			
442	2015		2015	KARNAL		KARNAL	NWPZ	NORMAL	NOT_HOSTILE	7.5	1	KRL_19	Triticum_aestivum	10	86	93	79	90	127	41.3	56	2.3	4.5	3300	34.0			
443	2015		2015	KARNAL		KARNAL	NWPZ	NORMAL	NOT_HOSTILE	7.5	1	HI_1563	Triticum_aestivum	11	85	93	88	81	128	42.8	45	1.9	4.4	3300	33.2			
444	2015		2015	KARNAL		KARNAL	NWPZ	NORMAL	NOT_HOSTILE	7.5	1	KHARCHIA_65	Triticum_aestivum	12	86	92	110	111	133	39.4	48	1.9	2.8	3700	18.7			
445	2015		2015	KARNAL		KARNAL	NWPZ	NORMAL	NOT_HOSTILE	7.5	1	HW_2044	Triticum_aestivum	13	83	89	87	125	127	50.0	55	2.7	5.8	3900	37.1			
446	2015		2015	KARNAL		KARNAL	NWPZ	NORMAL	NOT_HOSTILE	7.5	1	DBW_46	Triticum_aestivum	14	91	98	91	101	133	44.6	41	1.8	5.5	4100	33.4			
447	2015		2015	KARNAL		KARNAL	NWPZ	NORMAL	NOT_HOSTILE	7.5	1	RAJ_4238	Triticum_aestivum	15	85	92	85	112	127	46.4	44	2.0	4.7	3400	34.4			
448	2015		2015	KARNAL		KARNAL	NWPZ	NORMAL	NOT_HOSTILE	7.5	1	RAJ_4229	Triticum_aestivum	16	84	92	83	128	127	45.5	26	1.2	4.7	3300	35.7			
449	2015		2015	KARNAL		KARNAL	NWPZ	NORMAL	NOT_HOSTILE	7.5	1	HI_8498	Triticum_turgidum	17	93	101	82	80	142	58.3	36	2.1	4.6	3650	31.4			
450	2015		2015	KARNAL		KARNAL	NWPZ	NORMAL	NOT_HOSTILE	7.5	1	KRL_1-4	Triticum_aestivum	18	85	91	89	65	130	45.9	51	2.4	5.9	3900	37.5			
451	2015		2015	KARNAL		KARNAL	NWPZ	NORMAL	NOT_HOSTILE	7.5	1	UP_262	Triticum_aestivum	19	87	92	93	105	134	48.8	52	2.5	5.0	4100	30.2			
452	2015		2015	KARNAL		KARNAL	NWPZ	NORMAL	NOT_HOSTILE	7.5	1	DPW_621-50	Triticum_aestivum	20	91	97	85	76	139	46.5	70	3.3	6.4	4200	38.4			
453	2015		2015	KARNAL		KARNAL	NWPZ	NORMAL	NOT_HOSTILE	7.5	1	KRL_213	Triticum_aestivum	21	92	97	79	121	136	36.9	74	2.7	5.8	4100	35.6			
454	2015		2015	KARNAL		KARNAL	NWPZ	NORMAL	NOT_HOSTILE	7.5	1	KRL_3-4	Triticum_aestivum	22	86	92	110	104	132	28.0	38	1.1	1.9	2700	17.4			
455	2015		2015	KARNAL		KARNAL	NWPZ	NORMAL	NOT_HOSTILE	7.5	1	HD_2967	Triticum_aestivum	23	90	97	83	123	135	50.5	59	3.0	5.7	4700	30.3			
456	2015		2015	KARNAL		KARNAL	NWPZ	NORMAL	NOT_HOSTILE	7.5	1	GW_322	Triticum_aestivum	24	86	94	86	117	131	44.7	52	2.3	5.4	4000	34.0			
457	2015		2015	KARNAL		KARNAL	NWPZ	NORMAL	NOT_HOSTILE	7.5	1	WH_1021	Triticum_aestivum	25	87	94	83	120	130	46.5	47	2.2	5.5	4800	28.7			
458	2015		2015	KARNAL		KARNAL	NWPZ	NORMAL	NOT_HOSTILE	7.5	1	HD_2932	Triticum_aestivum	26	90	97	87	93	134	42.7	50	2.1	6.1	4600	33.0			
459	2015		2015	KARNAL		KARNAL	NWPZ	NORMAL	NOT_HOSTILE	7.5	1	MACS_6222	Triticum_aestivum	27	93	99	85	75	136	44.9	58	2.6	5.5	4400	31.5			
460	2015		2015	KARNAL		KARNAL	NWPZ	NORMAL	NOT_HOSTILE	7.5	1	DBW_17	Triticum_aestivum	28	88	93	79	107	131	44.0	43	1.9	5.9	3700	39.5			
461	2015		2015	KARNAL		KARNAL	NWPZ	NORMAL	NOT_HOSTILE	7.5	1	RW_3684	Triticum_aestivum	29	92	97	88	103	133	46.7	55	2.6	7.3	4900	37.3			
462	2015		2015	KARNAL		KARNAL	NWPZ	NORMAL	NOT_HOSTILE	7.5	1	PDW_314	Triticum_turgidum	30	96	104	85	121	139	47.8	56	2.7	6.6	4500	36.6			
463	2015		2015	KARNAL		KARNAL	NWPZ	NORMAL	NOT_HOSTILE	7.5	1	DBW_14	Triticum_aestivum	31	85	93	80	70	127	42.2	51	2.2	5.3	3400	38.8			
464	2015		2015	KARNAL		KARNAL	NWPZ	NORMAL	NOT_HOSTILE	7.5	1	KRL_210	Triticum_aestivum	32	86	92	88	90	131	50.0	43	2.2	6.6	3700	44.8			
465	2015		2015	KARNAL		KARNAL	NWPZ	NORMAL	NOT_HOSTILE	7.5	1	HD_2009	Triticum_aestivum	33	90	95	101	100	130	46.4	68	3.1	4.6	3700	30.8			
466	2015		2015	KARNAL		KARNAL	NWPZ	NORMAL	NOT_HOSTILE	7.5	1	DBW_71	Triticum_aestivum	34	86	91	88	114	130	47.9	48	2.3	5.4	3600	37.7			
467	2015		2015	KARNAL		KARNAL	NWPZ	NORMAL	NOT_HOSTILE	7.5	1	BH_1146	Triticum_aestivum	35	89	92	123	106	130	46.9	31	1.5	3.7	4000	23.3			
468	2015		2015	KARNAL		KARNAL	NWPZ	NORMAL	NOT_HOSTILE	7.5	1	K_0307	Triticum_aestivum	36	90	97	91	129	134	44.3	59	2.6	5.0	5600	22.1			
469	2015		2015	KARNAL		KARNAL	NWPZ	NORMAL	NOT_HOSTILE	7.5	2	GW_322	Triticum_aestivum	37	86	94	85	103	132	44.1	49	2.2	4.9	3400	36.2			
470	2015		2015	KARNAL		KARNAL	NWPZ	NORMAL	NOT_HOSTILE	7.5	2	WH_1105	Triticum_aestivum	38	89	96	85	101	132	40.9	69	2.8	6.6	3700	44.3			
471	2015		2015	KARNAL		KARNAL	NWPZ	NORMAL	NOT_HOSTILE	7.5	2	DBW_17	Triticum_aestivum	39	89	94	78	112	132	47.2	47	2.2	5.4	3300	40.6			
472	2015		2015	KARNAL		KARNAL	NWPZ	NORMAL	NOT_HOSTILE	7.5	2	BH_1146	Triticum_aestivum	40	88	92	123	111	131	44.9	38	1.7	3.7	3700	24.8			
473	2015		2015	KARNAL		KARNAL	NWPZ	NORMAL	NOT_HOSTILE	7.5	2	RAJ_4229	Triticum_aestivum	41	85	92	83	81	128	43.4	45	2.0	3.7	2800	33.3			
474	2015		2015	KARNAL		KARNAL	NWPZ	NORMAL	NOT_HOSTILE	7.5	2	NW_1067	Triticum_aestivum	42	90	97	84	103	133	34.9	55	1.9	6.5	4400	37.0			
475	2015		2015	KARNAL		KARNAL	NWPZ	NORMAL	NOT_HOSTILE	7.5	2	HI_1563	Triticum_aestivum	43	84	93	91	91	130	28.3	55	1.6	3.5	3200	27.7			
476	2015		2015	KARNAL		KARNAL	NWPZ	NORMAL	NOT_HOSTILE	7.5	2	RAJ_4238	Triticum_aestivum	44	84	92	87	117	128	34.5	49	1.7	3.4	3000	28.2			
477	2015		2015	KARNAL		KARNAL	NWPZ	NORMAL	NOT_HOSTILE	7.5	2	KRL_213	Triticum_aestivum	45	91	97	80	103	136	39.2	59	2.3	6.2	4200	36.9			
478	2015		2015	KARNAL		KARNAL	NWPZ	NORMAL	NOT_HOSTILE	7.5	2	HD_2932	Triticum_aestivum	46	89	96	88	115	135	36.2	52	1.9	5.8	4400	33.2			
479	2015		2015	KARNAL		KARNAL	NWPZ	NORMAL	NOT_HOSTILE	7.5	2	K_0307	Triticum_aestivum	47	90	97	91	127	135	47.4	50	2.4	4.2	4300	24.2			
480	2015		2015	KARNAL		KARNAL	NWPZ	NORMAL	NOT_HOSTILE	7.5	2	NW_4018	Triticum_aestivum	48	91	98	93	96	135	47.4	62	2.9	6.6	4400	37.3			
481	2015		2015	KARNAL		KARNAL	NWPZ	NORMAL	NOT_HOSTILE	7.5	2	WH_1021	Triticum_aestivum	49	87	94	89	135	130	42.2	50	2.1	5.8	4500	32.2			
482	2015		2015	KARNAL		KARNAL	NWPZ	NORMAL	NOT_HOSTILE	7.5	2	DBW_51	Triticum_aestivum	50	89	95	83	101	132	43.4	51	2.2	5.6	4500	31.1			
483	2015		2015	KARNAL		KARNAL	NWPZ	NORMAL	NOT_HOSTILE	7.5	2	HD_2733	Triticum_aestivum	51	90	97	80	135	134	49.5	38	1.9	4.7	4000	29.5			
484	2015		2015	KARNAL		KARNAL	NWPZ	NORMAL	NOT_HOSTILE	7.5	2	HW_2044	Triticum_aestivum	52	84	89	89	90	127	46.3	45	2.1	5.6	3500	40.0			

Abbreviations | **Description**

Samples!	Year!	Year1!	Year2!	Site_Name!	Site2014!	site2015!	ZONE!	Soil!	HOSTILE_NOT!	SOIL_pH!	Replication!	Genotype!	Species!	Plot_No!	DTH	DTA	PHT	PTM	DTM	TGW	GNS	GWS	GYD	BYD	HI
485	2015		2015	KARNAL		KARNAL	NWPZ	NORMAL	NOT_HOSTILE	7.5	2	DPW_621-50	Triticum_aestivum	53	88	98	83	97	137	47.4	63	3.0	6.4	4200	37.9
486	2015		2015	KARNAL		KARNAL	NWPZ	NORMAL	NOT_HOSTILE	7.5	2	DBW_71	Triticum_aestivum	54	86	91	88	89	131	42.6	44	1.9	6.7	4000	42.2
487	2015		2015	KARNAL		KARNAL	NWPZ	NORMAL	NOT_HOSTILE	7.5	2	KHARCHIA_65	Triticum_aestivum	55	88	94	112	128	136	42.6	43	1.8	2.6	3900	16.7
488	2015		2015	KARNAL		KARNAL	NWPZ	NORMAL	NOT_HOSTILE	7.5	2	UP_262	Triticum_aestivum	56	87	93	91	110	136	49.5	39	1.9	4.1	4000	25.8
489	2015		2015	KARNAL		KARNAL	NWPZ	NORMAL	NOT_HOSTILE	7.5	2	DBW_46	Triticum_aestivum	57	90	97	91	113	134	49.3	60	3.0	5.5	3900	35.1
490	2015		2015	KARNAL		KARNAL	NWPZ	NORMAL	NOT_HOSTILE	7.5	2	MACS_6222	Triticum_aestivum	58	94	100	86	106	137	46.1	56	2.6	5.2	3900	33.4
491	2015		2015	KARNAL		KARNAL	NWPZ	NORMAL	NOT_HOSTILE	7.5	2	DBW_16	Triticum_aestivum	59	90	97	79	108	134	33.6	49	1.6	4.9	3600	33.9
492	2015		2015	KARNAL		KARNAL	NWPZ	NORMAL	NOT_HOSTILE	7.5	2	HD_2009	Triticum_aestivum	60	89	94	99	102	131	48.9	47	2.3	4.7	4000	29.2
493	2015		2015	KARNAL		KARNAL	NWPZ	NORMAL	NOT_HOSTILE	7.5	2	KRL_210	Triticum_aestivum	61	89	92	90	103	132	49.9	43	2.1	6.9	4300	40.1
494	2015		2015	KARNAL		KARNAL	NWPZ	NORMAL	NOT_HOSTILE	7.5	2	CBW_38	Triticum_aestivum	62	97	97	89	98	131	48.2	43	2.1	5.3	4500	29.2
495	2015		2015	KARNAL		KARNAL	NWPZ	NORMAL	NOT_HOSTILE	7.5	2	PDW_314	Triticum_turgidum	63	85	104	85	110	139	48.4	53	2.6	6.0	4400	34.3
496	2015		2015	KARNAL		KARNAL	NWPZ	NORMAL	NOT_HOSTILE	7.5	2	KRL_3-4	Triticum_aestivum	64	93	91	109	112	132	29.2	43	1.3	2.6	3100	21.0
497	2015		2015	KARNAL		KARNAL	NWPZ	NORMAL	NOT_HOSTILE	7.5	2	HI_8498	Triticum_turgidum	65	94	101	81	117	141	63.8	41	2.6	3.9	3700	26.0
498	2015		2015	KARNAL		KARNAL	NWPZ	NORMAL	NOT_HOSTILE	7.5	2	NW_4092	Triticum_aestivum	66	85	101	84	119	140	44.8	50	2.3	6.9	5200	33.0
499	2015		2015	KARNAL		KARNAL	NWPZ	NORMAL	NOT_HOSTILE	7.5	2	KRL_1-4	Triticum_aestivum	67	85	91	89	81	131	47.7	53	2.5	5.7	3900	36.6
500	2015		2015	KARNAL		KARNAL	NWPZ	NORMAL	NOT_HOSTILE	7.5	2	DBW_14	Triticum_aestivum	68	89	93	80	94	128	45.0	49	2.2	5.0	3600	35.0
501	2015		2015	KARNAL		KARNAL	NWPZ	NORMAL	NOT_HOSTILE	7.5	2	DBW_39	Triticum_aestivum	69	90	94	83	114	133	49.9	52	2.6	6.2	3800	40.9
502	2015		2015	KARNAL		KARNAL	NWPZ	NORMAL	NOT_HOSTILE	7.5	2	HD_2967	Triticum_aestivum	70	90	97	84	108	135	50.2	49	2.5	5.2	3700	34.9
503	2015		2015	KARNAL		KARNAL	NWPZ	NORMAL	NOT_HOSTILE	7.5	2	KRL_19	Triticum_aestivum	71	87	94	80	101	129	33.9	52	1.8	4.1	3100	32.7
504	2015		2015	KARNAL		KARNAL	NWPZ	NORMAL	NOT_HOSTILE	7.5	2	RW_3684	Triticum_aestivum	72	91	97	91	91	133	45.8	54	2.5	7.2	4900	36.9
505	2015		2015	HISAR		HISAR	NWPZ	SALINE	HOSTILE	8.1	1	KRL_3-4	Triticum_aestivum	1	95	101	103	65	140	43.1	38	1.6	2.1	2000	25.8
506	2015		2015	HISAR		HISAR	NWPZ	SALINE	HOSTILE	8.1	1	KRL_19	Triticum_aestivum	2	95	101	72	20	140	32.3	50	1.6	0.8	700	30.0
507	2015		2015	HISAR		HISAR	NWPZ	SALINE	HOSTILE	8.1	1	GW_322	Triticum_aestivum	3	97	104	83	44	142	31.2	53	1.7	1.4	1100	31.3
508	2015		2015	HISAR		HISAR	NWPZ	SALINE	HOSTILE	8.1	1	NW_1067	Triticum_aestivum	4	97	105	90	81	142	35.8	40	1.4	3.9	2800	34.9
509	2015		2015	HISAR		HISAR	NWPZ	SALINE	HOSTILE	8.1	1	DBW_17	Triticum_aestivum	5	95	101	76	72	141	36.7	41	1.5	2.9	2100	34.1
510	2015		2015	HISAR		HISAR	NWPZ	SALINE	HOSTILE	8.1	1	HD_2967	Triticum_aestivum	6	98	105	92	76	143	40.4	53	2.2	3.8	2800	33.9
511	2015		2015	HISAR		HISAR	NWPZ	SALINE	HOSTILE	8.1	1	KRL_1-4	Triticum_aestivum	7	94	99	86	43	139	31.3	54	1.7	1.8	1500	30.3
512	2015		2015	HISAR		HISAR	NWPZ	SALINE	HOSTILE	8.1	1	HW_2044	Triticum_aestivum	8	92	99	85	45	135	32.1	37	1.2	1.5	1300	28.2
513	2015		2015	HISAR		HISAR	NWPZ	SALINE	HOSTILE	8.1	1	RAJ_4238	Triticum_aestivum	9	91	98	79	72	140	39.4	32	1.3	2.5	1900	32.7
514	2015		2015	HISAR		HISAR	NWPZ	SALINE	HOSTILE	8.1	1	HD_2932	Triticum_aestivum	10	97	104	88	72	143	34.8	40	1.4	3.3	2900	28.7
515	2015		2015	HISAR		HISAR	NWPZ	SALINE	HOSTILE	8.1	1	UP_262	Triticum_aestivum	11	96	103	92	51	142	41.4	37	1.5	2.1	2000	26.1
516	2015		2015	HISAR		HISAR	NWPZ	SALINE	HOSTILE	8.1	1	PDW_314	Triticum_turgidum	12	104	110	84	62	148	42.5	41	1.8	3.1	2400	32.1
517	2015		2015	HISAR		HISAR	NWPZ	SALINE	HOSTILE	8.1	1	BH_1146	Triticum_aestivum	13	92	99	124	94	139	37.6	36	1.3	2.5	2500	24.7
518	2015		2015	HISAR		HISAR	NWPZ	SALINE	HOSTILE	8.1	1	K_0307	Triticum_aestivum	14	101	107	93	32	143	36.3	49	1.8	2.0	1900	26.7
519	2015		2015	HISAR		HISAR	NWPZ	SALINE	HOSTILE	8.1	1	DBW_71	Triticum_aestivum	15	94	100	84	50	141	38.7	42	1.6	1.2	1100	26.4
520	2015		2015	HISAR		HISAR	NWPZ	SALINE	HOSTILE	8.1	1	NW_4092	Triticum_aestivum	16	99	106	95	54	143	29.2	54	1.6	1.3	1400	22.4
521	2015		2015	HISAR		HISAR	NWPZ	SALINE	HOSTILE	8.1	1	CBW_38	Triticum_aestivum	17	99	106	92	56	142	36.6	25	0.9	2.7	2100	32.0
522	2015		2015	HISAR		HISAR	NWPZ	SALINE	HOSTILE	8.1	1	DBW_46	Triticum_aestivum	18	98	104	96	79	141	36.4	57	2.1	2.7	2600	25.8
523	2015		2015	HISAR		HISAR	NWPZ	SALINE	HOSTILE	8.1	1	HD_2733	Triticum_aestivum	19	97	103	86	78	140	41.9	43	1.8	3.4	3500	24.1
524	2015		2015	HISAR		HISAR	NWPZ	SALINE	HOSTILE	8.1	1	DBW_51	Triticum_aestivum	20	96	103	94	70	140	34.7	50	1.7	3.0	2600	29.0
525	2015		2015	HISAR		HISAR	NWPZ	SALINE	HOSTILE	8.1	1	NW_4018	Triticum_aestivum	21	98	104	88	85	142	38.3	48	1.8	2.0	2400	21.3
526	2015		2015	HISAR		HISAR	NWPZ	SALINE	HOSTILE	8.1	1	HI_1563	Triticum_aestivum	22	92	98	89	43	135	36.8	31	1.1	2.1	2100	25.6
527	2015		2015	HISAR		HISAR	NWPZ	SALINE	HOSTILE	8.1	1	RAJ_4229	Triticum_aestivum	23	91	98	88	61	140	38.5	48	1.8	3.0	2300	32.8
528	2015		2015	HISAR		HISAR	NWPZ	SALINE	HOSTILE	8.1	1	HD_2009	Triticum_aestivum	24	94	101	100	114	142	40.1	37	1.5	2.6	3200	20.5
529	2015		2015	HISAR		HISAR	NWPZ	SALINE	HOSTILE	8.1	1	HI_8498	Triticum_turgidum	25	103	109	89	73	148	43.1	41	1.8	2.8	3000	23.7
530	2015		2015	HISAR		HISAR	NWPZ	SALINE	HOSTILE	8.1	1	RW_3684	Triticum_aestivum	26	98	104	97	83	143	43.8	43	1.9	3.5	3200	27.0
531	2015		2015	HISAR		HISAR	NWPZ	SALINE	HOSTILE	8.1	1	WH_1105	Triticum_aestivum	27	97	103	89	81	140	40.6	37	1.5	3.7	3100	29.8
532	2015		2015	HISAR		HISAR	NWPZ	SALINE	HOSTILE	8.1	1	DPW_621-50	Triticum_aestivum	28	99	104	91	117	142	40.1	55	2.2	3.9	3800	25.3
533	2015		2015	HISAR		HISAR	NWPZ	SALINE	HOSTILE	8.1	1	DBW_14	Triticum_aestivum	29	91	99	78	80	139	35.5	43	1.5	2.2	2300	23.6
534	2015		2015	HISAR		HISAR	NWPZ	SALINE	HOSTILE	8.1	1	KRL_210	Triticum_aestivum	30	94	101	94	94	143	45.8	59	2.7	5.0	4000	31.3
535	2015		2015	HISAR		HISAR	NWPZ	SALINE	HOSTILE	8.1	1	DBW_16	Triticum_aestivum	31	98	104	90	89	144	39.6	31	1.2	2.8	3600	19.7
536	2015		2015	HISAR		HISAR	NWPZ	SALINE	HOSTILE	8.1	1	MACS_6222	Triticum_aestivum	32	99	106	90	67	142	48.8	50	2.4	2.0	2500	20.4
537	2015		2015	HISAR		HISAR	NWPZ	SALINE	HOSTILE	8.1	1	WH_1021	Triticum_aestivum	33	94	100	98	132	139	41.3	60	2.5	3.4	3500	24.6
538	2015		2015	HISAR		HISAR	NWPZ	SALINE	HOSTILE	8.1	1	KRL_213	Triticum_aestivum	34	99	106	87	86	143	35.2	55	1.9	2.5	3000	21.2
539	2015		2015	HISAR		HISAR	NWPZ	SALINE	HOSTILE	8.1	1	KHARCHIA_65	Triticum_aestivum	35	94	99	112	109	138	39.6	37	1.5	1.5	3000	12.6
540	2015		2015	HISAR		HISAR	NWPZ	SALINE	HOSTILE	8.1	1	DBW_39	Triticum_aestivum	36	96	103	90	98	140	42.6	47	2.0	3.7	4000	23.1
541	2015		2015	HISAR		HISAR	NWPZ	SALINE	HOSTILE	8.1	2	WH_1021	Triticum_aestivum	37	93	99	99	85	140	41.9	48	2.0	3.4	3000	28.1
542	2015		2015	HISAR		HISAR	NWPZ	SALINE	HOSTILE	8.1	2	DBW_51	Triticum_aestivum	38	96	103	98	81	140	38.9	44	1.7	4.2	3000	35.3
543	2015		2015	HISAR		HISAR	NWPZ	SALINE	HOSTILE	8.1	2	DBW_46	Triticum_aestivum	39	97	104	98	74	142	45.9	34	1.6	3.1	2900	26.6
544	2015		2015	HISAR		HISAR	NWPZ	SALINE	HOSTILE	8.1	2	DPW_621-50	Triticum_aestivum	40	98	104	91	101	142	43.4	58	2.5	4.4	3700	30.1
545	2015		2015	HISAR		HISAR	NWPZ	SALINE	HOSTILE	8.1	2	HD_2932	Triticum_aestivum	41	98	104	92	92	141	41.2	45	1.9	3.8	3500	26.8
546	2015		2015	HISAR		HISAR	NWPZ	SALINE	HOSTILE	8.1	2	GW_322	Triticum_aestivum	42	96	103	83	80	143	38.3	49	1.9	2.7	2800	24.4
547	2015		2015	HISAR		HISAR	NWPZ	SALINE	HOSTILE	8.1	2	PDW_314	Triticum_turgidum	43	105	112	88	77	139	38.2	43	1.7	3.5	2900	30.2
548	2015		2015	HISAR		HISAR	NWPZ	SALINE	HOSTILE	8.1	2	KRL_3-4	Triticum_aestivum	44	94	100	104	97	135	42.8	33	1.4	3.2	3200	25.4
549	2015		2015	HISAR		HISAR	NWPZ	SALINE	HOSTILE	8.1	2	KHARCHIA_65	Triticum_aestivum	45	93	99	109	99	140	44.1	37	1.6	1.6	2400	16.9
550	2015		2015	HISAR		HISAR	NWPZ	SALINE	HOSTILE	8.1	2	HD_2009	Triticum_aestivum	46	95	103	108	96	143	42.2	58	2.4	2.2	3000	18.7
551	2015		2015	HISAR		HISAR	NWPZ	SALINE	HOSTILE	8.1	2	NW_4092	Triticum_aestivum	47	98	105	94	79	142	41.8	32	1.3	3.2	3000	26.8
552	2015		2015	HISAR		HISAR	NWPZ	SALINE	HOSTILE	8.1	2	RW_3684	Triticum_aestivum	48	98	103	99	86	148	45.5	45	2.0	3.7	3700	25.1
553	2015		2015	HISAR		HISAR	NWPZ	SALINE	HOSTILE	8.1	2	HW_2044	Triticum_aestivum	49	91	98	86	58	139	37.6	41	1.5	1.9	1900	25.0
554	2015		2015	HISAR		HISAR	NWPZ	SALINE	HOSTILE	8.1	2	DBW_14	Triticum_aestivum	50	90	98	80	64	143	38.2	36	1.4	2.5	2000	31.0
555	2015		2015	HISAR		HISAR	NWPZ	SALINE	HOSTILE	8.1	2	NW_4018	Triticum_aestivum	51	99	104	89	51	141	47.3	44	2.1	2.3	2500	23.2
556	2015		2015	HISAR		HISAR	NWPZ	SALINE	HOSTILE	8.1	2	BH_1146	Triticum_aestivum	52	92	100	122	82	143	40.0	32	1.3	2.4	3000	20.1
557	2015		2015	HISAR		HISAR	NWPZ	SALINE	HOSTILE	8.1	2	HD_2967	Triticum_aestivum	53	99	105	94	72	142	43.8	59	2.6	4.5	3700	30.3
558	2015		2015	HISAR		HISAR	NWPZ	SALINE	HOSTILE	8.1	2	KRL_213	Triticum_aestivum	54	100	106	89	62	141	36.9	62	2.3	3.2	3200	25.0
559	2015		2015	HISAR		HISAR	NWPZ	SALINE	HOSTILE	8.1	2	DBW_39	Triticum_aestivum	55	97	103	90	82	140	42.3	30	1.3	3.5	2900	30.1
560	2015		2015	HISAR		HISAR	NWPZ	SALINE	HOSTILE	8.1	2	HI_1563	Triticum_aestivum	56	91	99	89	58	140	37.3	35	1.3	1.7	1600	26.9
561	2015		2015	HISAR		HISAR	NWPZ	SALINE	HOSTILE	8.1	2	UP_262	Triticum_aestivum	57	95	102	94	90	142	43.8	47	2.1	2.8	3000	23.6
562	2015		2015	HISAR		HISAR	NWPZ	SALINE	HOSTILE	8.1	2	CBW_38	Triticum_aestivum	58	98	104	93	76	135	52.1	42	2.2	4.2	3700	28.1
563	2015		2015	HISAR		HISAR	NWPZ	SALINE	HOSTILE	8.1	2	DBW_17	Triticum_aestivum	59	94	101	82	115	140	43.8	39	1.7	2.9	2900	24.7
564	2015		2015	HISAR		HISAR	NWPZ	SALINE	HOSTILE	8.1	2	KRL_210	Triticum_aestivum	60	93	99	93	97	142	41.2	47	1.9	4.1	3800	27.1
565	2015		2015	HISAR		HISAR	NWPZ	SALINE	HOSTILE	8.1	2	MACS_6222	Triticum_aestivum	61	98	104	92	104	148	49.7	59	2.9	3.1	3000	25.7
566	2015		2015	HISAR		HISAR	NWPZ	SALINE	HOSTILE	8.1	2	HD_2733	Triticum_aestivum	62	97	102	87	104	143	42.1	23	1.0	3.6	3600	24.8
567	2015		2015	HISAR		HISAR	NWPZ	SALINE	HOSTILE	8.1	2	KRL_1-4	Triticum_aestivum	63	95	101	88	52	140	42.3	40	1.7	2.0	2000	25.1
568	2015		2015	HISAR		HISAR	NWPZ	SALINE	HOSTILE	8.1	2	K_0307	Triticum_aestivum	64	100	107	93	91	142	40.1	38	1.5	3.1	3500	22.4
569	2015		2015	HISAR		HISAR	NWPZ	SALINE	HOSTILE	8.1	2	KRL_19	Triticum_aestivum	65	95	101	77	61	139	33.3	58	1.9	1.8	2000	22.1
570	2015		2015	HISAR		HISAR	NWPZ	SALINE	HOSTILE	8.1	2	HI_8498	Triticum_turgidum	66	104	110	91	73	143	36.4	32	1.2	2.6	3000	21.5
571	2015		2015	HISAR		HISAR	NWPZ	SALINE	HOSTILE	8.1	2	WH_1105	Triticum_aestivum	67	96	102	90	88	144	41.1	63	2.6	4.3	3500	30.4
572	2015		2015	HISAR		HISAR	NWPZ	SALINE	HOSTILE	8.1	2	RAJ_4229	Triticum_aestivum	68	90	96	90	96	142	38.7	42	1.6	2.6	2500	25.7
573	2015		2015	HISAR		HISAR	NWPZ	SALINE	HOSTILE	8.1	2	NW_1067	Triticum_aestivum	69	97	103	91	98	139	36.4	46	1.7	4.0	3200	31.4
574	2015		2015	HISAR		HISAR	NWPZ	SALINE	HOSTILE	8.1	2	DBW_71	Triticum_aestivum	70	92	99	86	87	143	39.9	40	1.6	3.2	2800	28.4
575	2015		2015	HISAR		HISAR	NWPZ	SALINE	HOSTILE	8.1	2	RAJ_4238	Triticum_aestivum	71	91	98	81	81	138	37.8	47	1.8	2.9	2600	27.7
576	2015		2015	HISAR		HISAR	NWPZ	SALINE	HOSTILE	8.1	2	DBW_16	Triticum_aestivum	72	97	103	92	86	140	37.6	49	1.8	3.4	3000	28.0
577	2015		2015	KUMARGANJ-RECLAIMED		KUMARGANJ-RECLAIMED	NEPZ	NORMAL	NOT_HOSTILE	8.3	1	PDW_314	Triticum_turgidum	1	80	84	70	110	120	32.8	22	0.7	2.4	1750	34.3
578	2015		2015	KUMARGANJ-RECLAIMED		KUMARGANJ-RECLAIMED	NEPZ	NORMAL	NOT_HOSTILE	8.3	1	HD_2967	Triticum_aestivum	2	79	83	89	97	119	34.0	49	1.7	4.0	2450	40.8
579	2015		2015	KUMARGANJ-RECLAIMED		KUMARGANJ-RECLAIMED	NEPZ	NORMAL	NOT_HOSTILE	8.3	1	DPW_621-50	Triticum_aestivum	3	78	82	86	99	119	40.6	35	1.6	3.5	2500	35.0
580	2015		2015	KUMARGANJ-RECLAIMED		KUMARGANJ-RECLAIMED	NEPZ	NORMAL	NOT_HOSTILE	8.3	1	DBW_17	Triticum_aestivum	4	76	80	80	100	122	38.0	44	1.7	3.8	2150	44.2
581	2015		2015	KUMARGANJ-RECLAIMED		KUMARGANJ-RECLAIMED	NEPZ	NORMAL	NOT_HOSTILE	8.3	1	K_0307	Triticum_aestivum	5	76	79	92	113	124	39.9	36	1.5	4.6	2750	41.8
582	2015		2015	KUMARGANJ-RECLAIMED		KUMARGANJ-RECLAIMED	NEPZ	NORMAL	NOT_HOSTILE	8.3	1	BH_1146	Triticum_aestivum	6	73	77	120	125	127	35.9	33	1.3	3.4	2500	34.0
583	2015		2015	KUMARGANJ-RECLAIMED		KUMARGANJ-RECLAIMED	NEPZ	NORMAL	NOT_HOSTILE	8.3	1	RAJ_4229	Triticum_aestivum	7	70	73	76	121	119	35.4	32	1.2	2.4	1500	40.0
584	2015		2015	KUMARGANJ-RECLAIMED		KUMARGANJ-RECLAIMED	NEPZ	NORMAL	NOT_HOSTILE	8.3	1	DBW_14	Triticum_aestivum	8	68	73	72	92	119	40.2	31	1.3	3.1	1850	41.9
585	2015		2015	KUMARGANJ-RECLAIMED		KUMARGANJ-RECLAIMED	NEPZ	NORMAL	NOT_HOSTILE	8.3	1	DBW_46	Triticum_aestivum	9	78	81	92	107	121	34.2	39	1.4	3.3	2000	41.3
586	2015		2015	KUMARGANJ-RECLAIMED		KUMARGANJ-RECLAIMED	NEPZ	NORMAL	NOT_HOSTILE	8.3	1	KHARCHIA_65	Triticum_aestivum	10	73	77	105	134	119	38.1	22	0.6	1.2	1600	18.8
587	2015		2015	KUMARGANJ-RECLAIMED		KUMARGANJ-RECLAIMED	NEPZ	NORMAL	NOT_HOSTILE	8.3	1	DBW_16	Triticum_aestivum	11	79	82	84	106	119	28.3	44	1.3	3.6	2250	40.0
588	2015		2015	KUMARGANJ-RECLAIMED		KUMARGANJ-RECLAIMED	NEPZ	NORMAL	NOT_HOSTILE	8.3	1	HI_8498	Triticum_turgidum	12	78	83	72	78	123	48.7	28	1.4	2.8	1750	40.0
589	2015		2015	KUMARGANJ-RECLAIMED		KUMARGANJ-RECLAIMED	NEPZ	NORMAL	NOT_HOSTILE	8.3	1	MACS_6222	Triticum_aestivum	13	75	80	84	85	121	36.4	45	1.7	3.4	2100	40.5
590	2015		2015	KUMARGANJ-RECLAIMED		KUMARGANJ-RECLAIMED	NEPZ	NORMAL	NOT_HOSTILE	8.3	1	DBW_71	Triticum_aestivum	14	72	75	79	100	119	38.9	38	1.5	3.6	2100	42.9
591	2015		2015	KUMARGANJ-RECLAIMED		KUMARGANJ-RECLAIMED	NEPZ	NORMAL	NOT_HOSTILE	8.3	1	NW_4018	Triticum_aestivum	15	79	83	95	91	121	34.8	54	1.9	3.2	2300	34.8
592	2015		2015	KUMARGANJ-RECLAIMED		KUMARGANJ-RECLAIMED	NEPZ	NORMAL	NOT_HOSTILE	8.3	1	NW_4092	Triticum_aestivum	16	79	83	93	94	121	34.3	43	1.5	3.2	2250	35.6
593	2015		2015	KUMARGANJ-RECLAIMED		KUMARGANJ-RECLAIMED	NEPZ	NORMAL	NOT_HOSTILE	8.3	1	WH_1021	Triticum_aestivum	17	77	80	90	98	118	31.9	43	1.4	3.0	2250	33.3
594	2015		2015	KUMARGANJ-RECLAIMED		KUMARGANJ-RECLAIMED	NEPZ	NORMAL	NOT_HOSTILE	8.3	1	KRL_19	Triticum_aestivum	18	75	78	83	93	117	23.5	49	1.2	2.6	1800	36.1
595	2015		2015	KUMARGANJ-RECLAIMED		KUMARGANJ-RECLAIMED	NEPZ	NORMAL	NOT_HOSTILE	8.3	1	DBW_51	Triticum_aestivum	19	76	81	89	74	118	37.0	42	1.9	2.8	1750	40.0
596	2015		2015	KUMARGANJ-RECLAIMED		KUMARGANJ-RECLAIMED	NEPZ	NORMAL	NOT_HOSTILE	8.3	1	HI_1563	Triticum_aestivum	20	72	76	83	98	117	36.6	39	1.5	3.3	2425	34.0
597	2015		2015	KUMARGANJ-RECLAIMED		KUMARGANJ-RECLAIMED	NEPZ	NORMAL	NOT_HOSTILE	8.3	1	CBW_38	Triticum_aestivum	21	80	84	90	126	121	40.0	39	1.1	4.0	2850	35.1
598	2015		2015	KUMARGANJ-RECLAIMED		KUMARGANJ-RECLAIMED	NEPZ	NORMAL	NOT_HOSTILE	8.3	1	GW_322	Triticum_aestivum	22	73	77	79	97	119	33.5	42	1.6	3.0	2000	37.5
599	2015		2015	KUMARGANJ-RECLAIMED		KUMARGANJ-RECLAIMED	NEPZ	NORMAL	NOT_HOSTILE	8.3	1	RW_3684	Triticum_aestivum	23	80	84	92	113	123	33.7	42	1.4	3.6	2700	33.3
600	2015		2015	KUMARGANJ-RECLAIMED		KUMARGANJ-RECLAIMED	NEPZ	NORMAL	NOT_HOSTILE	8.3	1	KRL_3-4	Triticum_aestivum	24	74	78	105	105	120	40.9	35	1.5	2.9	2250	32.2
601	2015		2015	KUMARGANJ-RECLAIMED		KUMARGANJ-RECLAIMED	NEPZ	NORMAL	NOT_HOSTILE	8.3	1	KRL_1-4	Triticum_aestivum	25	69	74	85	61	117	42.3	38	1.6	2.3	1500	38.3
602	2015		2015	KUMARGANJ-RECLAIMED		KUMARGANJ-RECLAIMED	NEPZ	NORMAL	NOT_HOSTILE	8.3	1	UP_262	Triticum_aestivum	26	73	77	94	76	119	38.4	36	1.4	3.4	2225	38.2
603	2015		2015	KUMARGANJ-RECLAIMED		KUMARGANJ-RECLAIMED	NEPZ	NORMAL	NOT_HOSTILE	8.3	1	HD_2932	Triticum_aestivum	27	76	80	84	117	120	36.0	38	1.4	3.2	2300	34.8
604	2015		2015	KUMARGANJ-RECLAIMED		KUMARGANJ-RECLAIMED	NEPZ	NORMAL	NOT_HOSTILE	8.3	1	HW_2044	Triticum_aestivum	28	79	84	90	101	117	38.1	40	1.6	2.8	1950	35.9
605	2015		2015	KUMARGANJ-RECLAIMED		KUMARGANJ-RECLAIMED	NEPZ	NORMAL	NOT_HOSTILE	8.3	1	HD_2009	Triticum_aestivum	29	77	81	102	107	119	35.4	35	1.3	2.4	2025	29.6
606	2015		2015	KUMARGANJ-RECLAIMED		KUMARGANJ-RECLAIMED	NEPZ	NORMAL	NOT_HOSTILE	8.3	1	HD_2733	Triticum_aestivum	30	74	80	83	98	118	41.6	38	1.6	3.9	2300	42.4
607	2015		2015	KUMARGANJ-RECLAIMED		KUMARGANJ-RECLAIMED	NEPZ	NORMAL	NOT_HOSTILE	8.3	1	DBW_39	Triticum_aestivum	31	75	79	82	79	119	41.7	42	1.8	2.9	1750	41.4
608	2015		2015	KUMARGANJ-RECLAIMED		KUMARGANJ-RECLAIMED	NEPZ	NORMAL	NOT_HOSTILE	8.3	1	NW_1067	Triticum_aestivum	32	79	83	75	109	121	32.4	42	1.3	2.5	2100	29.8
609	2015		2015	KUMARGANJ-RECLAIMED		KUMARGANJ-RECLAIMED	NEPZ	NORMAL	NOT_HOSTILE	8.3	1	WH_1105	Triticum_aestivum	33	77	80	83	90	119	34.6	53	1.8	3.4	2075	41.0
610	2015		2015	KUMARGANJ-RECLAIMED		KUMARGANJ-RECLAIMED	NEPZ	NORMAL	NOT_HOSTILE	8.3	1	RAJ_4238	Triticum_aestivum	34	72	77	80	115	118	32.8	34	1.0	2.6	2000	32.5
611	2015		2015	KUMARGANJ-RECLAIMED		KUMARGANJ-RECLAIMED	NEPZ	NORMAL	NOT_HOSTILE	8.3	1	KRL_210	Triticum_aestivum	35	74	79	84	120	117	36.2	42	1.5	4.0	2750	36.4
612	2015		2015	KUMARGANJ-RECLAIMED		KUMARGANJ-RECLAIMED	NEPZ	NORMAL	NOT_HOSTILE	8.3	1	KRL_213	Triticum_aestivum	36	78	81	81	112	118	30.5	54	1.7	4.4	2600	42.3
613	2015		2015	KUMARGANJ-RECLAIMED		KUMARGANJ-RECLAIMED	NEPZ	NORMAL	NOT_HOSTILE	8.3	2	KRL_3-4	Triticum_aestivum	37	73	77	103	94	118	38.5	32	1.0	2.8	2100	33.3
614	2015		2015	KUMARGANJ-RECLAIMED		KUMARGANJ-RECLAIMED	NEPZ	NORMAL	NOT_HOSTILE	8.3	2	UP_262	Triticum_aestivum	38	74	79	90	100	118	40.0	45	1.8	3.7	2350	39.1
615	2015		2015	KUMARGANJ-RECLAIMED		KUMARGANJ-RECLAIMED	NEPZ	NORMAL	NOT_HOSTILE	8.3	2	KHARCHIA_65	Triticum_aestivum	39	73	78	108	116	119	42.5	23	1.4	1.8	2100	21.0
616	2015		2015	KUMARGANJ-RECLAIMED		KUMARGANJ-RECLAIMED	NEPZ	NORMAL	NOT_HOSTILE	8.3	2	DBW_17	Triticum_aestivum	40	76	80	79	104	122	39.7	43	1.8	3.2	2100	38.1
617	2015		2015	KUMARGANJ-RECLAIMED		KUMARGANJ-RECLAIMED	NEPZ	NORMAL	NOT_HOSTILE	8.3	2	DBW_71	Triticum_aestivum	41	71	75	82	108	118	43.1	37	1.6	4.1	2300	44.3
618	2015		2015	KUMARGANJ-RECLAIMED		KUMARGANJ-RECLAIMED	NEPZ	NORMAL	NOT_HOSTILE	8.3	2	DBW_39	Triticum_aestivum	42	74	77	86	98	117	42.6	43	1.9	4.0	2075	48.2
619	2015		2015	KUMARGANJ-RECLAIMED		KUMARGANJ-RECLAIMED	NEPZ	NORMAL	NOT_HOSTILE	8.3	2	WH_1021	Triticum_aestivum	43	78	82	91	75	119	35.5	44	1.8	2.6	2000	32.5
620	2015		2015	KUMARGANJ-RECLAIMED		KUMARGANJ-RECLAIMED	NEPZ	NORMAL	NOT_HOSTILE	8.3	2	KRL_210	Triticum_aestivum	44	73	78	91	78	118	34.3	36	1.3	3.4	2275	37.4
621	2015		2015	KUMARGANJ-RECLAIMED		KUMARGANJ-RECLAIMED	NEPZ	NORMAL	NOT_HOSTILE	8.3	2	DBW_16	Triticum_aestivum	45	78	81	83	86	120	29.8	44	1.3	3.3	2250	36.4
622	2015		2015	KUMARGANJ-RECLAIMED		KUMARGANJ-RECLAIMED	NEPZ	NORMAL	NOT_HOSTILE	8.3	2	HD_2009	Triticum_aestivum	46	75	79	105	70	119	40.4	47	1.9	2.8	2325	30.1
623	2015		2015	KUMARGANJ-RECLAIMED		KUMARGANJ-RECLAIMED	NEPZ	NORMAL	NOT_HOSTILE	8.3	2	CBW_38	Triticum_aestivum	47	79	83	96	115	120	40.0	36	1.4	3.8	2825	34.0
624	2015		2015	KUMARGANJ-RECLAIMED		KUMARGANJ-RECLAIMED	NEPZ	NORMAL	NOT_HOSTILE	8.3	2	DPW_621-50	Triticum_aestivum	48	75	81	90	78	118	54.8	33	1.7	3.8	2475	38.0
625	2015		2015	KUMARGANJ-RECLAIMED		KUMARGANJ-RECLAIMED	NEPZ	NORMAL	NOT_HOSTILE	8.3	2	DBW_46	Triticum_aestivum	49	75	80	91	102	120	47.4	38	1.6	2.8	2200	31.8
626	2015		2015	KUMARGANJ-RECLAIMED		KUMARGANJ-RECLAIMED	NEPZ	NORMAL	NOT_HOSTILE	8.3	2	RAJ_4238	Triticum_aestivum	50	73	78	75	79	120	37.2	25	0.9	2.6	1850	35.7
627	2015		2015	KUMARGANJ-RECLAIMED		KUMARGANJ-RECLAIMED	NEPZ	NORMAL	NOT_HOSTILE	8.3	2	NW_4092	Triticum_aestivum	51	78	82	90	76	121	42.8	40	1.7	3.2	2350	34.0
628	2015		2015	KUMARGANJ-RECLAIMED		KUMARGANJ-RECLAIMED	NEPZ	NORMAL	NOT_HOSTILE	8.3	2	KRL_1-4	Triticum_aestivum	52	68	74	84	103	118	38.9	35	1.4	2.5	1750	35.4
629	2015		2015	KUMARGANJ-RECLAIMED		KUMARGANJ-RECLAIMED	NEPZ	NORMAL	NOT_HOSTILE	8.3	2	K_0307	Triticum_aestivum	53	74	79	88	84	123	38.8	37	1.5	2.8	2100	33.3
630	2015		2015	KUMARGANJ-RECLAIMED		KUMARGANJ-RECLAIMED	NEPZ	NORMAL	NOT_HOSTILE	8.3	2	GW_322	Triticum_aestivum	54	74	78	83	99	120	35.3	42	1.5	3.3	2200	37.3
631	2015		2015	KUMARGANJ-RECLAIMED		KUMARGANJ-RECLAIMED	NEPZ	NORMAL	NOT_HOSTILE	8.3	2	DBW_51	Triticum_aestivum	55	77	82	90	72	120	39.4	56	2.5	3.0	1900	40.0
632	2015		2015	KUMARGANJ-RECLAIMED		KUMARGANJ-RECLAIMED	NEPZ	NORMAL	NOT_HOSTILE	8.3	2	PDW_314	Triticum_turgidum	56	80	85	75	76	122	34.2	27	1.0	2.0	1625	30.8
633	2015		2015	KUMARGANJ-RECLAIMED		KUMARGANJ-RECLAIMED	NEPZ	NORMAL	NOT_HOSTILE	8.3	2	MACS_6222	Triticum_aestivum	57	77	81	82	85	121	32.5	46	1.4	3.0	2250	32.9
634	2015		2015	KUMARGANJ-RECLAIMED		KUMARGANJ-RECLAIMED	NEPZ	NORMAL	NOT_HOSTILE	8.3	2	HD_2932	Triticum_aestivum	58	76	81	85	94	119	35.9	43	1.6	2.6	2000	33.0
635	2015		2015	KUMARGANJ-RECLAIMED		KUMARGANJ-RECLAIMED	NEPZ	NORMAL	NOT_HOSTILE	8.3	2	KRL_213	Triticum_aestivum	59	76	80	81	97	120	31.5	56	1.8	4.0	2325	43.0
636	2015		2015	KUMARGANJ-RECLAIMED		KUMARGANJ-RECLAIMED	NEPZ	NORMAL	NOT_HOSTILE	8.3	2	RAJ_4229	Triticum_aestivum	60	72	75	73	116	118	38.8	31	1.2	2.6	1800	36.7
637	2015		2015	KUMARGANJ-RECLAIMED		KUMARGANJ-RECLAIMED	NEPZ	NORMAL	NOT_HOSTILE	8.3	2	WH_1105	Triticum_aestivum	61	75	79	82	75	118	30.8	45	1.6	2.8	1575	44.4
638	2015		2015	KUMARGANJ-RECLAIMED		KUMARGANJ-RECLAIMED	NEPZ	NORMAL	NOT_HOSTILE	8.3	2	HD_2967	Triticum_aestivum	62	76	80	86	83	119	37.3	42	1.6	3.4	2250	38.2
639	2015		2015	KUMARGANJ-RECLAIMED		KUMARGANJ-RECLAIMED	NEPZ	NORMAL	NOT_HOSTILE	8.3	2	HD_2733	Triticum_aestivum	63	75	81	85	105	120	45.2	35	1.6	3.4	2200	38.2
640	2015		2015	KUMARGANJ-RECLAIMED		KUMARGANJ-RECLAIMED	NEPZ	NORMAL	NOT_HOSTILE	8.3	2	NW_4018	Triticum_aestivum	64	79	82	86	101	122	30.0	51	1.5	2.4	2075	28.9
641	2015		2015	KUMARGANJ-RECLAIMED		KUMARGANJ-RECLAIMED	NEPZ	NORMAL	NOT_HOSTILE	8.3	2	HI_1563	Triticum_aestivum	65	73	77	88	94	116	32.6	34	1.2	3.0	2350	31.5
642	2015		2015	KUMARGANJ-RECLAIMED		KUMARGANJ-RECLAIMED	NEPZ	NORMAL	NOT_HOSTILE	8.3	2	DBW_14	Triticum_aestivum	66	70	74	76	103	119	38.1	40	1.6	3.2	1500	53.3
643	2015		2015	KUMARGANJ-RECLAIMED		KUMARGANJ-RECLAIMED	NEPZ	NORMAL	NOT_HOSTILE	8.3	2	HI_8498	Triticum_turgidum	67	75	79	68	76	120	45.9	36	1.7	2.0	1500	33.3
644	2015		2015	KUMARGANJ-RECLAIMED		KUMARGANJ-RECLAIMED	NEPZ	NORMAL	NOT_HOSTILE	8.3	2	BH_1146	Triticum_aestivum	68	73	76	115	74	125	32.5	34	1.0	3.2	2250	35.6
645	2015		2015	KUMARGANJ-RECLAIMED		KUMARGANJ-RECLAIMED	NEPZ	NORMAL	NOT_HOSTILE	8.3	2	NW_1067	Triticum_aestivum	69	78	81	76	114	122	36.7	41	1.5	3.0	2275	32.5
646	2015		2015	KUMARGANJ-RECLAIMED		KUMARGANJ-RECLAIMED	NEPZ	NORMAL	NOT_HOSTILE	8.3	2	RW_3684	Triticum_aestivum	70	78	81	90	87	121	35.0	40	1.5	3.4	2500	34.4
647	2015		2015	KUMARGANJ-RECLAIMED		KUMARGANJ-RECLAIMED	NEPZ	NORMAL	NOT_HOSTILE	8.3	2	KRL_19	Triticum_aestivum	71	74	77	83	104	119	25.1	44	1.1	2.4	2000	30.0
648	2015		2015	KUMARGANJ-RECLAIMED		KUMARGANJ-RECLAIMED	NEPZ	NORMAL	NOT_HOSTILE	8.3	2	HW_2044	Triticum_aestivum	72	75	80	86	123	117	35.8	36	1.2	3.9	2500	39.2
649	2015		2015	KUMARGANJ-SODIC		KUMARGANJ-SODIC	NEPZ	SODIC	HOSTILE	9.5	1	PDW_314	Triticum_turgidum	1	78	81	65	38	117	20.2	25	0.5	0.5	350	35.7
650	2015		2015	KUMARGANJ-SODIC		KUMARGANJ-SODIC	NEPZ	SODIC	HOSTILE	9.5	1	HD_2967	Triticum_aestivum	2	77	80	75	30	115	35.0	40	1.4	1.1	525	52.4
651	2015		2015	KUMARGANJ-SODIC		KUMARGANJ-SODIC	NEPZ	SODIC	HOSTILE	9.5	1	DPW_621-50	Triticum_aestivum	3	77	81	61	33	114	31.8	49	1.6	1.5	750	50.0
652	2015		2015	KUMARGANJ-SODIC		KUMARGANJ-SODIC	NEPZ	SODIC	HOSTILE	9.5	1	DBW_17	Triticum_aestivum	4	76	80	74	37	116	25.0	31	0.8	0.6	350	42.9
653	2015		2015	KUMARGANJ-SODIC		KUMARGANJ-SODIC	NEPZ	SODIC	HOSTILE	9.5	1	K_0307	Triticum_aestivum	5	75	79	85	46	118	29.9	32	1.0	0.6	375	40.0
654	2015		2015	KUMARGANJ-SODIC		KUMARGANJ-SODIC	NEPZ	SODIC	HOSTILE	9.5	1	BH_1146	Triticum_aestivum	6	71	75	82	42	115	29.8	34	1.3	1.6	950	42.1
655	2015		2015	KUMARGANJ-SODIC		KUMARGANJ-SODIC	NEPZ	SODIC	HOSTILE	9.5	1	RAJ_4229	Triticum_aestivum	7	70	73	63	31	113	32.7	36	1.2	1.0	600	41.7
656	2015		2015	KUMARGANJ-SODIC		KUMARGANJ-SODIC	NEPZ	SODIC	HOSTILE	9.5	1	DBW_14	Triticum_aestivum	8	66	70	61	47	113	38.6	37	1.5	1.0	500	50.0
657	2015		2015	KUMARGANJ-SODIC		KUMARGANJ-SODIC	NEPZ	SODIC	HOSTILE	9.5	1	DBW_46	Triticum_aestivum	9	77	81	72	35	116	38.2	45	1.8	1.1	550	50.0
658	2015		2015	KUMARGANJ-SODIC		KUMARGANJ-SODIC	NEPZ	SODIC	HOSTILE	9.5	1	KHARCHIA_65	Triticum_aestivum	10	70	74	85	67	117	33.5	41	1.6	1.1	600	45.8
659	2015		2015	KUMARGANJ-SODIC		KUMARGANJ-SODIC	NEPZ	SODIC	HOSTILE	9.5	1	DBW_16	Triticum_aestivum	11	80	85	60	27	116	31.5	40	1.3	0.8	450	44.4
660	2015		2015	KUMARGANJ-SODIC		KUMARGANJ-SODIC	NEPZ	SODIC	HOSTILE	9.5	1	HI_8498	Triticum_turgidum	12	77	82	62	32	117	33.2	44	1.5	1.0	650	38.5
661	2015		2015	KUMARGANJ-SODIC		KUMARGANJ-SODIC	NEPZ	SODIC	HOSTILE	9.5	1	MACS_6222	Triticum_aestivum	13	74	80	72	43	115	35.0	19	1.2	1.0	600	41.7
662	2015		2015	KUMARGANJ-SODIC		KUMARGANJ-SODIC	NEPZ	SODIC	HOSTILE	9.5	1	DBW_71	Triticum_aestivum	14	70	75	63	38	114	34.2	40	1.4	0.7	350	50.0
663	2015		2015	KUMARGANJ-SODIC		KUMARGANJ-SODIC	NEPZ	SODIC	HOSTILE	9.5	1	NW_4018	Triticum_aestivum	15	77	81	71	30	116	32.8	42	1.4	1.0	485	51.5
664	2015		2015	KUMARGANJ-SODIC		KUMARGANJ-SODIC	NEPZ	SODIC	HOSTILE	9.5	1	NW_4092	Triticum_aestivum	16	78	83	72	35	115	38.8	42	1.7	1.1	600	45.8
665	2015		2015	KUMARGANJ-SODIC		KUMARGANJ-SODIC	NEPZ	SODIC	HOSTILE	9.5	1	WH_1021	Triticum_aestivum	17	75	79	72	38	114	28.7	45	1.3	1.1	600	45.8
666	2015		2015	KUMARGANJ-SODIC		KUMARGANJ-SODIC	NEPZ	SODIC	HOSTILE	9.5	1	KRL_19	Triticum_aestivum	18	73	78	61	36	113	21.5	62	1.4	1.4	975	35.9
667	2015		2015	KUMARGANJ-SODIC		KUMARGANJ-SODIC	NEPZ	SODIC	HOSTILE	9.5	1	DBW_51	Triticum_aestivum	19	74	79	73	31	114	28.0	43	1.2	1.2	750	40.0
668	2015		2015	KUMARGANJ-SODIC		KUMARGANJ-SODIC	NEPZ	SODIC	HOSTILE	9.5	1	HI_1563	Triticum_aestivum	20	70	75	74	46	113	33.8	43	1.5	1.2	700	42.9
669	2015		2015	KUMARGANJ-SODIC		KUMARGANJ-SODIC	NEPZ	SODIC	HOSTILE	9.5	1	CBW_38	Triticum_aestivum	21	81	85	65	32	116	30.2	25	0.8	0.7	380	46.1
670	2015		2015	KUMARGANJ-SODIC		KUMARGANJ-SODIC	NEPZ	SODIC	HOSTILE	9.5	1	GW_322	Triticum_aestivum	22	71	74	73	37	114	28.4	41	1.2	1.3	700	46.4
671	2015		2015	KUMARGANJ-SODIC		KUMARGANJ-SODIC	NEPZ	SODIC	HOSTILE	9.5	1	RW_3684	Triticum_aestivum	23	78	82	71	23	118	28.9	33	0.9	1.0	600	41.7
672	2015		2015	KUMARGANJ-SODIC		KUMARGANJ-SODIC	NEPZ	SODIC	HOSTILE	9.5	1	KRL_3-4	Triticum_aestivum	24	72	77	80	54	115	38.3	43	1.7	1.6	975	41.0
673	2015		2015	KUMARGANJ-SODIC		KUMARGANJ-SODIC	NEPZ	SODIC	HOSTILE	9.5	1	KRL_1-4	Triticum_aestivum	25	70	74	86	40	112	27.9	37	1.1	1.5	825	46.7
674	2015		2015	KUMARGANJ-SODIC		KUMARGANJ-SODIC	NEPZ	SODIC	HOSTILE	9.5	1	UP_262	Triticum_aestivum	26	70	75	83	48	115	32.4	30	1.0	1.1	600	45.8
675	2015		2015	KUMARGANJ-SODIC		KUMARGANJ-SODIC	NEPZ	SODIC	HOSTILE	9.5	1	HD_2932	Triticum_aestivum	27	74	78	75	58	116	36.3	48	1.8	1.6	750	53.3
676	2015		2015	KUMARGANJ-SODIC		KUMARGANJ-SODIC	NEPZ	SODIC	HOSTILE	9.5	1	HW_2044	Triticum_aestivum	28	77	81	73	43	114	37.8	41	1.6	1.4	850	41.2
677	2015		2015	KUMARGANJ-SODIC		KUMARGANJ-SODIC	NEPZ	SODIC	HOSTILE	9.5	1	HD_2009	Triticum_aestivum	29	74	77	82	38	116	37.0	51	1.9	1.4	800	43.8
678	2015		2015	KUMARGANJ-SODIC		KUMARGANJ-SODIC	NEPZ	SODIC	HOSTILE	9.5	1	HD_2733	Triticum_aestivum	30	72	75	75	69	115	34.7	36	1.3	1.9	1075	44.2
679	2015		2015	KUMARGANJ-SODIC		KUMARGANJ-SODIC	NEPZ	SODIC	HOSTILE	9.5	1	DBW_39	Triticum_aestivum	31	73	78	65	42	115	33.0	35	1.2	0.8	500	40.0
680	2015		2015	KUMARGANJ-SODIC		KUMARGANJ-SODIC	NEPZ	SODIC	HOSTILE	9.5	1	NW_1067	Triticum_aestivum	32	80	85	62	53	117	28.8	34	1.0	1.4	750	46.7
681	2015		2015	KUMARGANJ-SODIC		KUMARGANJ-SODIC	NEPZ	SODIC	HOSTILE	9.5	1	WH_1105	Triticum_aestivum	33	77	81	66	41	116	36.7	42	1.6	1.1	600	45.8
682	2015		2015	KUMARGANJ-SODIC		KUMARGANJ-SODIC	NEPZ	SODIC	HOSTILE	9.5	1	RAJ_4238	Triticum_aestivum	34	72	80	73	70	114	37.4	42	1.6	2.5	1075	58.1
683	2015		2015	KUMARGANJ-SODIC		KUMARGANJ-SODIC	NEPZ	SODIC	HOSTILE	9.5	1	KRL_210	Triticum_aestivum	35	72	74	73	39	114	41.1	40	1.7	1.3	725	44.8
684	2015		2015	KUMARGANJ-SODIC		KUMARGANJ-SODIC	NEPZ	SODIC	HOSTILE	9.5	1	KRL_213	Triticum_aestivum	36	77	81	70	43	115	27.5	55	1.6	2.0	1200	41.7
685	2015		2015	KUMARGANJ-SODIC		KUMARGANJ-SODIC	NEPZ	SODIC	HOSTILE	9.5	2	KRL_3-4	Triticum_aestivum	37	70	75	90	57	113	36.5	48	1.9	1.2	1200	25.0
686	2015		2015	KUMARGANJ-SODIC		KUMARGANJ-SODIC	NEPZ	SODIC	HOSTILE	9.5	2	UP_262	Triticum_aestivum	38	68	72	82	65	113	30.4	30	1.3	2.0	1400	35.7
687	2015		2015	KUMARGANJ-SODIC		KUMARGANJ-SODIC	NEPZ	SODIC	HOSTILE	9.5	2	KHARCHIA_65	Triticum_aestivum	39	71	76	84	49	115	33.5	37	1.5	1.2	1100	27.3
688	2015		2015	KUMARGANJ-SODIC		KUMARGANJ-SODIC	NEPZ	SODIC	HOSTILE	9.5	2	DBW_17	Triticum_aestivum	40	77	83	64	72	114	27.9	34	0.9	1.2	800	37.5
689	2015		2015	KUMARGANJ-SODIC		KUMARGANJ-SODIC	NEPZ	SODIC	HOSTILE	9.5	2	DBW_71	Triticum_aestivum	41	71	77	71	56	114	34.8	40	1.1	2.3	1000	57.5
690	2015		2015	KUMARGANJ-SODIC		KUMARGANJ-SODIC	NEPZ	SODIC	HOSTILE	9.5	2	DBW_39	Triticum_aestivum	42	74	79	66	41	115	33.4	35	1.3	1.4	1350	25.9
691	2015		2015	KUMARGANJ-SODIC		KUMARGANJ-SODIC	NEPZ	SODIC	HOSTILE	9.5	2	WH_1021	Triticum_aestivum	43	73	78	81	62	116	30.7	48	1.4	2.0	1500	33.3
692	2015		2015	KUMARGANJ-SODIC		KUMARGANJ-SODIC	NEPZ	SODIC	HOSTILE	9.5	2	KRL_210	Triticum_aestivum	44	73	76	72	67	115	40.5	38	1.6	2.4	1700	35.3
693	2015		2015	KUMARGANJ-SODIC		KUMARGANJ-SODIC	NEPZ	SODIC	HOSTILE	9.5	2	DBW_16	Triticum_aestivum	45	77	87	65	56	116	30.8	45	1.2	1.2	1000	30.0
694	2015		2015	KUMARGANJ-SODIC		KUMARGANJ-SODIC	NEPZ	SODIC	HOSTILE	9.5	2	HD_2009	Triticum_aestivum	46	72	75	90	59	115	39.6	57	1.8	2.0	1500	33.3
695	2015		2015	KUMARGANJ-SODIC		KUMARGANJ-SODIC	NEPZ	SODIC	HOSTILE	9.5	2	CBW_38	Triticum_aestivum	47	80	84	71	75	116	28.0	21	0.9	2.1	1775	29.6
696	2015		2015	KUMARGANJ-SODIC		KUMARGANJ-SODIC	NEPZ	SODIC	HOSTILE	9.5	2	DPW_621-50	Triticum_aestivum	48	77	82	68	40	116	29.6	45	1.5	1.6	1300	30.8
697	2015		2015	KUMARGANJ-SODIC		KUMARGANJ-SODIC	NEPZ	SODIC	HOSTILE	9.5	2	DBW_46	Triticum_aestivum	49	75	82	81	47	114	36.2	41	1.5	1.5	1125	33.3
698	2015		2015	KUMARGANJ-SODIC		KUMARGANJ-SODIC	NEPZ	SODIC	HOSTILE	9.5	2	RAJ_4238	Triticum_aestivum	50	70	78	72	52	113	31.7	45	1.5	2.4	1150	52.2
699	2015		2015	KUMARGANJ-SODIC		KUMARGANJ-SODIC	NEPZ	SODIC	HOSTILE	9.5	2	NW_4092	Triticum_aestivum	51	77	84	79	45	117	40.5	46	1.8	1.6	1200	33.3
700	2015		2015	KUMARGANJ-SODIC		KUMARGANJ-SODIC	NEPZ	SODIC	HOSTILE	9.5	2	KRL_1-4	Triticum_aestivum	52	69	75	76	59	113	25.9	35	1.2	1.3	1200	27.1
701	2015		2015	KUMARGANJ-SODIC		KUMARGANJ-SODIC	NEPZ	SODIC	HOSTILE	9.5	2	K_0307	Triticum_aestivum	53	74	78	82	54	115	33.4	41	1.3	2.0	1700	29.4
702	2015		2015	KUMARGANJ-SODIC		KUMARGANJ-SODIC	NEPZ	SODIC	HOSTILE	9.5	2	GW_322	Triticum_aestivum	54	72	76	71	71	113	30.6	30	1.2	1.6	1300	30.8
703	2015		2015	KUMARGANJ-SODIC		KUMARGANJ-SODIC	NEPZ	SODIC	HOSTILE	9.5	2	DBW_51	Triticum_aestivum	55	72	76	80	42	114	30.4	40	1.4	1.3	1100	29.5
704	2015		2015	KUMARGANJ-SODIC		KUMARGANJ-SODIC	NEPZ	SODIC	HOSTILE	9.5	2	PDW_314	Triticum_turgidum	56	81	85	70	48	118	22.5	31	0.7	0.8	850	23.5
705	2015		2015	KUMARGANJ-SODIC		KUMARGANJ-SODIC	NEPZ	SODIC	HOSTILE	9.5	2	MACS_6222	Triticum_aestivum	57	73	78	73	35	116	33.2	26	1.1	1.0	700	35.7
706	2015		2015	KUMARGANJ-SODIC		KUMARGANJ-SODIC	NEPZ	SODIC	HOSTILE	9.5	2	HD_2932	Triticum_aestivum	58	71	76	74	67	115	36.3	53	1.7	2.5	1500	41.7
707	2015		2015	KUMARGANJ-SODIC		KUMARGANJ-SODIC	NEPZ	SODIC	HOSTILE	9.5	2	KRL_213	Triticum_aestivum	59	76	80	74	65	116	29.6	60	1.4	2.0	1300	38.5
708	2015		2015	KUMARGANJ-SODIC		KUMARGANJ-SODIC	NEPZ	SODIC	HOSTILE	9.5	2	RAJ_4229	Triticum_aestivum	60	71	74	69	50	114	35.8	40	1.2	1.1	700	39.3
709	2015		2015	KUMARGANJ-SODIC		KUMARGANJ-SODIC	NEPZ	SODIC	HOSTILE	9.5	2	WH_1105	Triticum_aestivum	61	76	82	69	43	115	29.9	44	1.6	1.6	1150	34.8
710	2015		2015	KUMARGANJ-SODIC		KUMARGANJ-SODIC	NEPZ	SODIC	HOSTILE	9.5	2	HD_2967	Triticum_aestivum	62	74	78	72	48	116	34.5	38	1.2	2.0	1400	35.7
711	2015		2015	KUMARGANJ-SODIC		KUMARGANJ-SODIC	NEPZ	SODIC	HOSTILE	9.5	2	HD_2733	Triticum_aestivum	63	70	74	65	57	115	31.9	41	1.5	1.5	850	44.1
712	2015		2015	KUMARGANJ-SODIC		KUMARGANJ-SODIC	NEPZ	SODIC	HOSTILE	9.5	2	NW_4018	Triticum_aestivum	64	76	82	77	54	117	30.9	43	1.6	1.4	1200	29.2
713	2015		2015	KUMARGANJ-SODIC		KUMARGANJ-SODIC	NEPZ	SODIC	HOSTILE	9.5	2	HI_1563	Triticum_aestivum	65	69	74	78	53	113	36.1	47	1.3	1.9	1350	35.2
714	2015		2015	KUMARGANJ-SODIC		KUMARGANJ-SODIC	NEPZ	SODIC	HOSTILE	9.5	2	DBW_14	Triticum_aestivum	66	68	69	65	51	113	40.7	32	1.6	1.4	950	36.8
715	2015		2015	KUMARGANJ-SODIC		KUMARGANJ-SODIC	NEPZ	SODIC	HOSTILE	9.5	2	HI_8498	Triticum_turgidum	67	75	80	69	36	117	35.7	48	1.4	1.2	1000	30.0
716	2015		2015	KUMARGANJ-SODIC		KUMARGANJ-SODIC	NEPZ	SODIC	HOSTILE	9.5	2	BH_1146	Triticum_aestivum	68	74	77	85	99	113	27.1	39	1.4	2.9	2000	36.3
717	2015		2015	KUMARGANJ-SODIC		KUMARGANJ-SODIC	NEPZ	SODIC	HOSTILE	9.5	2	NW_1067	Triticum_aestivum	69	78	83	69	63	116	25.8	38	1.1	2.0	1500	33.3
718	2015		2015	KUMARGANJ-SODIC		KUMARGANJ-SODIC	NEPZ	SODIC	HOSTILE	9.5	2	RW_3684	Triticum_aestivum	70	80	83	80	54	117	26.7	39	1.1	1.2	1100	27.3
719	2015		2015	KUMARGANJ-SODIC		KUMARGANJ-SODIC	NEPZ	SODIC	HOSTILE	9.5	2	KRL_19	Triticum_aestivum	71	70	79	63	56	114	20.3	52	1.2	1.9	1400	33.9
720	2015		2015	KUMARGANJ-SODIC		KUMARGANJ-SODIC	NEPZ	SODIC	HOSTILE	9.5	2	HW_2044	Triticum_aestivum	72	75	80	72	61	113	37.0	40	1.5	1.6	1125	35.6
721	2015		2015	PUNDIBARI		PUNDIBARI	NEPZ	ACIDIC	HOSTILE	5.3	1	RW_3684	Triticum_aestivum	1	80	87	97	105	118	47.9	50	2.4	5.2	7000	18.4
722	2015		2015	PUNDIBARI		PUNDIBARI	NEPZ	ACIDIC	HOSTILE	5.3	1	DBW_14	Triticum_aestivum	2	57	62	72	116	95	44.7	53	2.4	4.2	2900	35.9
723	2015		2015	PUNDIBARI		PUNDIBARI	NEPZ	ACIDIC	HOSTILE	5.3	1	KRL_1-4	Triticum_aestivum	3	64	71	82	89	106	40.2	54	2.2	3.6	2900	31.4
724	2015		2015	PUNDIBARI		PUNDIBARI	NEPZ	ACIDIC	HOSTILE	5.3	1	HW_2044	Triticum_aestivum	4	60	66	89	133	104	43.6	50	2.2	4.0	3400	29.7
725	2015		2015	PUNDIBARI		PUNDIBARI	NEPZ	ACIDIC	HOSTILE	5.3	1	PDW_314	Triticum_turgidum	5	79	88	76	69	118	47.6	50	2.4	3.2	2700	29.6
726	2015		2015	PUNDIBARI		PUNDIBARI	NEPZ	ACIDIC	HOSTILE	5.3	1	KRL_213	Triticum_aestivum	6	78	87	86	121	118	36.4	77	2.8	6.0	6500	23.1
727	2015		2015	PUNDIBARI		PUNDIBARI	NEPZ	ACIDIC	HOSTILE	5.3	1	HI_8498	Triticum_turgidum	7	71	81	80	66	108	47.5	64	3.0	3.2	2400	32.9

Samples!	Year!	Year1!	Year2!	Site_Name!	Site2014!	site2015!	ZONE!	Soil!	HOSTILE_NOT!	SOIL_ pH!	Replication!	Genotype!	Species!	Plot_No!	DTH	DTA	PHT	PTM	DTM	TGW	GNS	GWS	GYD	BYD	HI		Abbreviations	Description
728	2015		2015	PUNDIBARI		PUNDIBARI	NEPZ	ACIDIC	HOSTILE	5.3	1	GW_322	Triticum_aestivum	8	72	81	83	95	111	43.2	61	2.6	4.0	4400	22.7			
729	2015		2015	PUNDIBARI		PUNDIBARI	NEPZ	ACIDIC	HOSTILE	5.3	1	DBW_71	Triticum_aestivum	9	65	72	85	106	104	41.9	46	1.9	6.4	4200	38.1			
730	2015		2015	PUNDIBARI		PUNDIBARI	NEPZ	ACIDIC	HOSTILE	5.3	1	WH_1105	Triticum_aestivum	10	72	79	88	71	109	37.8	78	3.0	3.7	2900	32.1			
731	2015		2015	PUNDIBARI		PUNDIBARI	NEPZ	ACIDIC	HOSTILE	5.3	1	RAJ_4229	Triticum_aestivum	11	61	64	82	127	106	38.0	43	1.6	4.0	3000	33.3			
732	2015		2015	PUNDIBARI		PUNDIBARI	NEPZ	ACIDIC	HOSTILE	5.3	1	BH_1146	Triticum_aestivum	12	68	75	117	120	111	40.1	33	1.3	5.2	6300	20.6			
733	2015		2015	PUNDIBARI		PUNDIBARI	NEPZ	ACIDIC	HOSTILE	5.3	1	HD_2009	Triticum_aestivum	13	62	69	114	114	110	52.6	60	3.2	5.0	5300	23.8			
734	2015		2015	PUNDIBARI		PUNDIBARI	NEPZ	ACIDIC	HOSTILE	5.3	1	DBW_17	Triticum_aestivum	14	80	87	84	102	117	41.0	56	2.3	4.4	8100	13.5			
735	2015		2015	PUNDIBARI		PUNDIBARI	NEPZ	ACIDIC	HOSTILE	5.3	1	WH_1021	Triticum_aestivum	15	65	72	93	117	104	48.5	40	1.9	4.0	4300	23.3			
736	2015		2015	PUNDIBARI		PUNDIBARI	NEPZ	ACIDIC	HOSTILE	5.3	1	UP_262	Triticum_aestivum	16	69	74	102	117	110	50.2	51	2.6	5.1	5800	22.1			
737	2015		2015	PUNDIBARI		PUNDIBARI	NEPZ	ACIDIC	HOSTILE	5.3	1	HD_2967	Triticum_aestivum	17	76	81	93	102	116	43.4	57	2.5	5.2	7100	18.3			
738	2015		2015	PUNDIBARI		PUNDIBARI	NEPZ	ACIDIC	HOSTILE	5.3	1	DBW_16	Triticum_aestivum	18	85	91	92	119	119	38.6	50	1.9	5.7	9200	15.4			
739	2015		2015	PUNDIBARI		PUNDIBARI	NEPZ	ACIDIC	HOSTILE	5.3	1	NW_1067	Triticum_aestivum	19	71	78	90	110	113	34.8	47	1.6	3.7	4100	22.7			
740	2015		2015	PUNDIBARI		PUNDIBARI	NEPZ	ACIDIC	HOSTILE	5.3	1	HI_1563	Triticum_aestivum	20	58	63	93	77	104	44.7	49	2.2	3.6	3700	24.3			
741	2015		2015	PUNDIBARI		PUNDIBARI	NEPZ	ACIDIC	HOSTILE	5.3	1	KRL_3-4	Triticum_aestivum	21	72	78	124	91	119	39.6	40	1.6	4.0	6700	14.9			
742	2015		2015	PUNDIBARI		PUNDIBARI	NEPZ	ACIDIC	HOSTILE	5.3	1	DBW_46	Triticum_aestivum	22	77	86	100	91	114	46.7	50	2.3	4.0	5100	19.6			
743	2015		2015	PUNDIBARI		PUNDIBARI	NEPZ	ACIDIC	HOSTILE	5.3	1	NW_4092	Triticum_aestivum	23	78	86	99	71	114	43.7	65	2.8	3.8	5100	18.6			
744	2015		2015	PUNDIBARI		PUNDIBARI	NEPZ	ACIDIC	HOSTILE	5.3	1	HD_2733	Triticum_aestivum	24	79	87	87	89	117	46.9	42	2.0	3.7	6600	13.9			
745	2015		2015	PUNDIBARI		PUNDIBARI	NEPZ	ACIDIC	HOSTILE	5.3	1	KRL_210	Triticum_aestivum	25	71	78	96	91	106	39.1	53	2.1	4.5	4500	25.1			
746	2015		2015	PUNDIBARI		PUNDIBARI	NEPZ	ACIDIC	HOSTILE	5.3	1	NW_4018	Triticum_aestivum	26	80	86	94	90	115	40.0	64	2.6	4.0	4600	21.7			
747	2015		2015	PUNDIBARI		PUNDIBARI	NEPZ	ACIDIC	HOSTILE	5.3	1	RAJ_4238	Triticum_aestivum	27	58	65	81	133	95	41.2	40	1.6	3.4	3100	27.4			
748	2015		2015	PUNDIBARI		PUNDIBARI	NEPZ	ACIDIC	HOSTILE	5.3	1	KHARCHIA_65	Triticum_aestivum	28	71	79	119	99	116	43.9	45	2.0	3.7	3900	23.6			
749	2015		2015	PUNDIBARI		PUNDIBARI	NEPZ	ACIDIC	HOSTILE	5.3	1	CBW_38	Triticum_aestivum	29	74	81	95	126	114	36.5	37	1.4	4.2	5100	20.8			
750	2015		2015	PUNDIBARI		PUNDIBARI	NEPZ	ACIDIC	HOSTILE	5.3	1	KRL_19	Triticum_aestivum	30	60	65	79	105	104	42.8	50	2.1	3.6	3300	27.6			
751	2015		2015	PUNDIBARI		PUNDIBARI	NEPZ	ACIDIC	HOSTILE	5.3	1	DBW_51	Triticum_aestivum	31	76	82	91	72	114	41.8	60	2.5	3.9	4200	23.3			
752	2015		2015	PUNDIBARI		PUNDIBARI	NEPZ	ACIDIC	HOSTILE	5.3	1	K_0307	Triticum_aestivum	32	71	78	95	90	113	40.8	46	1.9	4.8	5000	24.0			
753	2015		2015	PUNDIBARI		PUNDIBARI	NEPZ	ACIDIC	HOSTILE	5.3	1	DPW_621-50	Triticum_aestivum	33	76	81	86	99	115	41.1	56	2.3	4.0	4800	20.8			
754	2015		2015	PUNDIBARI		PUNDIBARI	NEPZ	ACIDIC	HOSTILE	5.3	1	HD_2932	Triticum_aestivum	34	65	73	89	114	106	46.0	45	2.1	4.6	4700	24.3			
755	2015		2015	PUNDIBARI		PUNDIBARI	NEPZ	ACIDIC	HOSTILE	5.3	1	MACS_6222	Triticum_aestivum	35	73	83	89	89	113	40.6	57	2.3	4.0	4800	20.8			
756	2015		2015	PUNDIBARI		PUNDIBARI	NEPZ	ACIDIC	HOSTILE	5.3	1	DBW_39	Triticum_aestivum	36	76	82	98	72	113	47.3	59	2.8	4.2	5400	19.3			
757	2015		2015	PUNDIBARI		PUNDIBARI	NEPZ	ACIDIC	HOSTILE	5.3	2	MACS_6222	Triticum_aestivum	37	71	81	75	93	110	40.9	49	2.0	3.2	4300	18.6			
758	2015		2015	PUNDIBARI		PUNDIBARI	NEPZ	ACIDIC	HOSTILE	5.3	2	WH_1021	Triticum_aestivum	38	69	75	80	121	109	42.1	54	2.3	3.6	4100	22.0			
759	2015		2015	PUNDIBARI		PUNDIBARI	NEPZ	ACIDIC	HOSTILE	5.3	2	HD_2733	Triticum_aestivum	39	80	87	85	93	108	49.0	44	2.1	3.7	5000	18.6			
760	2015		2015	PUNDIBARI		PUNDIBARI	NEPZ	ACIDIC	HOSTILE	5.3	2	HW_2044	Triticum_aestivum	40	61	66	80	137	108	41.5	44	1.8	2.7	3200	21.3			
761	2015		2015	PUNDIBARI		PUNDIBARI	NEPZ	ACIDIC	HOSTILE	5.3	2	BH_1146	Triticum_aestivum	41	68	75	127	124	110	45.8	37	1.7	3.7	6100	15.1			
762	2015		2015	PUNDIBARI		PUNDIBARI	NEPZ	ACIDIC	HOSTILE	5.3	2	KRL_19	Triticum_aestivum	42	60	67	78	109	109	35.8	50	1.8	3.3	4000	20.8			
763	2015		2015	PUNDIBARI		PUNDIBARI	NEPZ	ACIDIC	HOSTILE	5.3	2	DBW_71	Triticum_aestivum	43	69	76	82	110	111	41.3	52	2.1	4.2	5000	21.0			
764	2015		2015	PUNDIBARI		PUNDIBARI	NEPZ	ACIDIC	HOSTILE	5.3	2	DPW_621-50	Triticum_aestivum	44	78	84	85	103	116	43.9	54	2.4	3.2	5000	16.2			
765	2015		2015	PUNDIBARI		PUNDIBARI	NEPZ	ACIDIC	HOSTILE	5.3	2	HD_2967	Triticum_aestivum	45	78	86	82	106	115	43.8	57	2.5	3.6	4800	18.8			
766	2015		2015	PUNDIBARI		PUNDIBARI	NEPZ	ACIDIC	HOSTILE	5.3	2	HI_1563	Triticum_aestivum	46	61	68	81	79	109	40.7	44	1.8	2.8	3100	22.9			
767	2015		2015	PUNDIBARI		PUNDIBARI	NEPZ	ACIDIC	HOSTILE	5.3	2	NW_4018	Triticum_aestivum	47	81	87	100	92	115	40.2	54	2.2	3.8	5300	17.9			
768	2015		2015	PUNDIBARI		PUNDIBARI	NEPZ	ACIDIC	HOSTILE	5.3	2	KRL_213	Triticum_aestivum	48	79	86	86	118	117	34.2	59	2.0	4.9	5100	23.9			
769	2015		2015	PUNDIBARI		PUNDIBARI	NEPZ	ACIDIC	HOSTILE	5.3	2	KHARCHIA_65	Triticum_aestivum	49	71	79	123	97	119	44.8	41	1.8	3.8	6200	15.3			
770	2015		2015	PUNDIBARI		PUNDIBARI	NEPZ	ACIDIC	HOSTILE	5.3	2	RW_3684	Triticum_aestivum	50	78	85	94	102	114	41.7	50	2.1	4.6	5900	19.3			
771	2015		2015	PUNDIBARI		PUNDIBARI	NEPZ	ACIDIC	HOSTILE	5.3	2	NW_4092	Triticum_aestivum	51	78	86	96	69	115	42.8	56	2.4	4.7	6800	17.4			
772	2015		2015	PUNDIBARI		PUNDIBARI	NEPZ	ACIDIC	HOSTILE	5.3	2	GW_322	Triticum_aestivum	52	72	79	94	93	111	45.2	46	2.1	4.0	5800	17.2			
773	2015		2015	PUNDIBARI		PUNDIBARI	NEPZ	ACIDIC	HOSTILE	5.3	2	UP_262	Triticum_aestivum	53	71	75	104	115	110	48.0	51	2.5	5.3	5900	22.4			
774	2015		2015	PUNDIBARI		PUNDIBARI	NEPZ	ACIDIC	HOSTILE	5.3	2	DBW_51	Triticum_aestivum	54	78	86	102	70	115	45.2	58	2.6	4.1	5000	20.4			
775	2015		2015	PUNDIBARI		PUNDIBARI	NEPZ	ACIDIC	HOSTILE	5.3	2	RAJ_4238	Triticum_aestivum	55	57	62	75	130	97	52.9	36	1.9	5.3	4200	31.4			
776	2015		2015	PUNDIBARI		PUNDIBARI	NEPZ	ACIDIC	HOSTILE	5.3	2	DBW_17	Triticum_aestivum	56	76	84	74	99	114	41.6	47	1.9	4.2	4600	23.0			
777	2015		2015	PUNDIBARI		PUNDIBARI	NEPZ	ACIDIC	HOSTILE	5.3	2	KRL_1-4	Triticum_aestivum	57	58	68	78	90	99	51.6	43	2.2	3.6	3200	27.8			
778	2015		2015	PUNDIBARI		PUNDIBARI	NEPZ	ACIDIC	HOSTILE	5.3	2	WH_1105	Triticum_aestivum	58	69	78	84	72	104	38.3	48	1.8	3.2	3100	25.8			
779	2015		2015	PUNDIBARI		PUNDIBARI	NEPZ	ACIDIC	HOSTILE	5.3	2	NW_1067	Triticum_aestivum	59	68	78	84	112	106	39.7	44	1.7	3.4	4100	20.7			
780	2015		2015	PUNDIBARI		PUNDIBARI	NEPZ	ACIDIC	HOSTILE	5.3	2	DBW_39	Triticum_aestivum	60	74	81	92	73	112	48.8	39	1.9	4.6	5000	23.2			
781	2015		2015	PUNDIBARI		PUNDIBARI	NEPZ	ACIDIC	HOSTILE	5.3	2	HD_2009	Triticum_aestivum	61	65	71	97	119	109	38.9	42	1.6	3.6	4100	21.7			
782	2015		2015	PUNDIBARI		PUNDIBARI	NEPZ	ACIDIC	HOSTILE	5.3	2	K_0307	Triticum_aestivum	62	71	76	101	95	111	45.6	46	2.1	4.5	5300	21.3			
783	2015		2015	PUNDIBARI		PUNDIBARI	NEPZ	ACIDIC	HOSTILE	5.3	2	KRL_3-4	Triticum_aestivum	63	74	81	121	96	119	46.1	37	1.7	4.2	6500	16.0			
784	2015		2015	PUNDIBARI		PUNDIBARI	NEPZ	ACIDIC	HOSTILE	5.3	2	PDW_314	Triticum_turgidum	64	76	83	77	73	116	49.7	35	1.8	2.8	3000	23.7			
785	2015		2015	PUNDIBARI		PUNDIBARI	NEPZ	ACIDIC	HOSTILE	5.3	2	KRL_210	Triticum_aestivum	65	71	78	92	96	110	42.3	45	1.9	4.8	6100	19.8			
786	2015		2015	PUNDIBARI		PUNDIBARI	NEPZ	ACIDIC	HOSTILE	5.3	2	HI_8498	Triticum_turgidum	66	71	81	80	70	112	52.3	37	1.9	2.6	3100	21.0			
787	2015		2015	PUNDIBARI		PUNDIBARI	NEPZ	ACIDIC	HOSTILE	5.3	2	DBW_46	Triticum_aestivum	67	74	82	105	96	113	46.7	37	1.7	4.6	7700	14.8			
788	2015		2015	PUNDIBARI		PUNDIBARI	NEPZ	ACIDIC	HOSTILE	5.3	2	RAJ_4229	Triticum_aestivum	68	58	63	79	132	95	46.3	39	1.8	4.2	3700	28.4			
789	2015		2015	PUNDIBARI		PUNDIBARI	NEPZ	ACIDIC	HOSTILE	5.3	2	CBW_38	Triticum_aestivum	69	75	81	95	128	113	42.3	43	1.8	4.1	6100	16.9			
790	2015		2015	PUNDIBARI		PUNDIBARI	NEPZ	ACIDIC	HOSTILE	5.3	2	DBW_14	Triticum_aestivum	70	55	60	73	121	90	49.7	39	1.9	3.6	3900	23.3			
791	2015		2015	PUNDIBARI		PUNDIBARI	NEPZ	ACIDIC	HOSTILE	5.3	2	HD_2932	Triticum_aestivum	71	62	68	95	119	104	46.4	49	2.3	4.2	4700	22.3			
792	2015		2015	PUNDIBARI		PUNDIBARI	NEPZ	ACIDIC	HOSTILE	5.3	2	DBW_16	Triticum_aestivum	72	82	88	91	125	119	39.5	54	2.1	4.8	6300	18.9			
793	2015		2015	MALDA		MALDA	NEPZ	NORMAL	NOT_HOSTILE	7.7	1	RW_3684	Triticum_aestivum	1	76	86	110	85	109	35.5	44	1.4	5.3	4150	31.7			
794	2015		2015	MALDA		MALDA	NEPZ	NORMAL	NOT_HOSTILE	7.7	1	DBW_14	Triticum_aestivum	2	62	75	94	90	107	31.8	42	1.4	5.3	3950	33.7			
795	2015		2015	MALDA		MALDA	NEPZ	NORMAL	NOT_HOSTILE	7.7	1	KRL_1-4	Triticum_aestivum	3	65	76	105	78	110	33.6	45	1.4	6.4	4300	37.2			
796	2015		2015	MALDA		MALDA	NEPZ	NORMAL	NOT_HOSTILE	7.7	1	HW_2044	Triticum_aestivum	4	62	74	108	63	111	33.2	38	1.4	5.7	4400	32.5			
797	2015		2015	MALDA		MALDA	NEPZ	NORMAL	NOT_HOSTILE	7.7	1	PDW_314	Triticum_turgidum	5	74	87	104	101	113	43.2	43	1.7	6.2	4900	31.6			
798	2015		2015	MALDA		MALDA	NEPZ	NORMAL	NOT_HOSTILE	7.7	1	KRL_213	Triticum_aestivum	6	78	87	100	87	115	32.1	52	1.7	5.7	4150	34.3			
799	2015		2015	MALDA		MALDA	NEPZ	NORMAL	NOT_HOSTILE	7.7	1	HI_8498	Triticum_turgidum	7	67	86	99	79	109	47.5	48	1.6	4.0	2850	35.1			
800	2015		2015	MALDA		MALDA	NEPZ	NORMAL	NOT_HOSTILE	7.7	1	GW_322	Triticum_aestivum	8	71	88	96	102	111	32.9	45	1.6	5.5	4450	30.9			
801	2015		2015	MALDA		MALDA	NEPZ	NORMAL	NOT_HOSTILE	7.7	1	DBW_71	Triticum_aestivum	9	67	81	100	89	111	34.5	47	1.5	6.5	4800	33.8			
802	2015		2015	MALDA		MALDA	NEPZ	NORMAL	NOT_HOSTILE	7.7	1	WH_1105	Triticum_aestivum	10	71	83	111	65	111	32.1	60	1.6	4.5	3550	31.8			
803	2015		2015	MALDA		MALDA	NEPZ	NORMAL	NOT_HOSTILE	7.7	1	RAJ_4229	Triticum_aestivum	11	65	73	100	64	103	33.3	43	1.5	5.4	3950	34.4			
804	2015		2015	MALDA		MALDA	NEPZ	NORMAL	NOT_HOSTILE	7.7	1	BH_1146	Triticum_aestivum	12	65	74	163	82	107	38.8	34	1.4	5.2	5000	26.1			
805	2015		2015	MALDA		MALDA	NEPZ	NORMAL	NOT_HOSTILE	7.7	1	HD_2009	Triticum_aestivum	13	71	83	130	87	109	36.9	48	1.6	4.5	3650	30.8			
806	2015		2015	MALDA		MALDA	NEPZ	NORMAL	NOT_HOSTILE	7.7	1	DBW_17	Triticum_aestivum	14	81	88	93	79	115	39.7	45	1.6	4.9	3400	36.2			
807	2015		2015	MALDA		MALDA	NEPZ	NORMAL	NOT_HOSTILE	7.7	1	WH_1021	Triticum_aestivum	15	67	81	116	78	112	34.2	43	1.3	6.1	4700	32.6			
808	2015		2015	MALDA		MALDA	NEPZ	NORMAL	NOT_HOSTILE	7.7	1	UP_262	Triticum_aestivum	16	67	81	119	92	112	38.8	33	1.5	5.3	4100	32.3			
809	2015		2015	MALDA		MALDA	NEPZ	NORMAL	NOT_HOSTILE	7.7	1	HD_2967	Triticum_aestivum	17	78	87	109	90	119	37.4	56	1.7	5.6	3850	36.5			
810	2015		2015	MALDA		MALDA	NEPZ	NORMAL	NOT_HOSTILE	7.7	1	DBW_16	Triticum_aestivum	18	81	90	95	80	119	36.5	41	1.3	5.7	4300	33.0			
811	2015		2015	MALDA		MALDA	NEPZ	NORMAL	NOT_HOSTILE	7.7	1	NW_1067	Triticum_aestivum	19	74	87	94	75	112	33.9	41	1.3	3.9	2900	33.4			
812	2015		2015	MALDA		MALDA	NEPZ	NORMAL	NOT_HOSTILE	7.7	1	HI_1563	Triticum_aestivum	20	65	78	106	86	109	34.2	44	1.5	5.2	3950	32.9			
813	2015		2015	MALDA		MALDA	NEPZ	NORMAL	NOT_HOSTILE	7.7	1	KRL_3-4	Triticum_aestivum	21	74	87	144	42	119	22.2	31	1.1	1.6	3850	10.5			
814	2015		2015	MALDA		MALDA	NEPZ	NORMAL	NOT_HOSTILE	7.7	1	DBW_46	Triticum_aestivum	22	74	89	116	81	115	37.1	36	1.3	3.6	3300	27.6			
815	2015		2015	MALDA		MALDA	NEPZ	NORMAL	NOT_HOSTILE	7.7	1	NW_4092	Triticum_aestivum	23	79	88	111	90	119	40.3	44	1.5	5.3	4400	30.2			
816	2015		2015	MALDA		MALDA	NEPZ	NORMAL	NOT_HOSTILE	7.7	1	HD_2733	Triticum_aestivum	24	71	87	103	85	115	46.2	32	1.2	4.7	4500	26.1			
817	2015		2015	MALDA		MALDA	NEPZ	NORMAL	NOT_HOSTILE	7.7	1	KRL_210	Triticum_aestivum	25	71	84	100	67	109	36.7	42	1.4	4.5	3250	34.3			
818	2015		2015	MALDA		MALDA	NEPZ	NORMAL	NOT_HOSTILE	7.7	1	NW_4018	Triticum_aestivum	26	78	88	107	81	109	39.4	40	1.3	4.0	3350	30.0			
819	2015		2015	MALDA		MALDA	NEPZ	NORMAL	NOT_HOSTILE	7.7	1	RAJ_4238	Triticum_aestivum	27	65	81	97	99	103	33.0	39	1.4	4.8	3450	34.6			
820	2015		2015	MALDA		MALDA	NEPZ	NORMAL	NOT_HOSTILE	7.7	1	KHARCHIA_65	Triticum_aestivum	28	71	84	156	53	113	31.5	35	1.4	2.6	3050	21.1			
821	2015		2015	MALDA		MALDA	NEPZ	NORMAL	NOT_HOSTILE	7.7	1	CBW_38	Triticum_aestivum	29	76	86	114	93	112	37.5	38	1.4	4.9	3750	32.4			
822	2015		2015	MALDA		MALDA	NEPZ	NORMAL	NOT_HOSTILE	7.7	1	KRL_19	Triticum_aestivum	30	65	81	96	79	105	29.4	45	1.5	4.1	3250	31.2			
823	2015		2015	MALDA		MALDA	NEPZ	NORMAL	NOT_HOSTILE	7.7	1	DBW_51	Triticum_aestivum	31	79	87	107	69	112	38.4	49	1.7	4.3	3450	31.4			
824	2015		2015	MALDA		MALDA	NEPZ	NORMAL	NOT_HOSTILE	7.7	1	K_0307	Triticum_aestivum	32	76	86	115	81	111	38.6	38	1.5	4.8	3750	32.0			
825	2015		2015	MALDA		MALDA	NEPZ	NORMAL	NOT_HOSTILE	7.7	1	DPW_621-50	Triticum_aestivum	33	76	87	99	79	119	44.9	36	1.6	2.9	4250	17.3			
826	2015		2015	MALDA		MALDA	NEPZ	NORMAL	NOT_HOSTILE	7.7	1	HD_2932	Triticum_aestivum	34	69	84	99	87	105	33.1	46	1.6	4.6	3500	32.6			
827	2015		2015	MALDA		MALDA	NEPZ	NORMAL	NOT_HOSTILE	7.7	1	MACS_6222	Triticum_aestivum	35	71	87	106	86	111	34.8	44	1.5	4.9	3500	34.9			
828	2015		2015	MALDA		MALDA	NEPZ	NORMAL	NOT_HOSTILE	7.7	1	DBW_39	Triticum_aestivum	36	74	86	101	84	112	45.8	37	1.6	4.8	3900	30.9			
829	2015		2015	MALDA		MALDA	NEPZ	NORMAL	NOT_HOSTILE	7.7	2	MACS_6222	Triticum_aestivum	37	74	88	104	72	112	38.4	59	1.7	4.7	3300	35.5			
830	2015		2015	MALDA		MALDA	NEPZ	NORMAL	NOT_HOSTILE	7.7	2	WH_1021	Triticum_aestivum	38	69	81	103	70	109	41.2	43	1.4	5.3	3600	36.8			
831	2015		2015	MALDA		MALDA	NEPZ	NORMAL	NOT_HOSTILE	7.7	2	HD_2733	Triticum_aestivum	39	79	89	101	89	118	46.0	36	1.7	4.1	4500	22.8			
832	2015		2015	MALDA		MALDA	NEPZ	NORMAL	NOT_HOSTILE	7.7	2	HW_2044	Triticum_aestivum	40	60	72	106	83	111	34.6	40	1.2	4.5	3300	33.8			
833	2015		2015	MALDA		MALDA	NEPZ	NORMAL	NOT_HOSTILE	7.7	2	BH_1146	Triticum_aestivum	41	67	78	159	67	107	36.0	41	1.4	3.8	3950	24.3			
834	2015		2015	MALDA		MALDA	NEPZ	NORMAL	NOT_HOSTILE	7.7	2	KRL_19	Triticum_aestivum	42	69	81	97	70	104	28.3	53	1.5	4.1	3300	30.9			
835	2015		2015	MALDA		MALDA	NEPZ	NORMAL	NOT_HOSTILE	7.7	2	DBW_71	Triticum_aestivum	43	69	81	103	72	109	38.2	45	1.7	4.5	3000	37.2			
836	2015		2015	MALDA		MALDA	NEPZ	NORMAL	NOT_HOSTILE	7.7	2	DPW_621-50	Triticum_aestivum	44	76	87	105	79	118	35.8	42	1.5	4.2	3350	31.6			
837	2015		2015	MALDA		MALDA	NEPZ	NORMAL	NOT_HOSTILE	7.7	2	HD_2967	Triticum_aestivum	45	78	87	105	87	118	38.4	56	1.7	5.5	4300	31.9			
838	2015		2015	MALDA		MALDA	NEPZ	NORMAL	NOT_HOSTILE	7.7	2	HI_1563	Triticum_aestivum	46	65	87	114	84	109	32.9	40	1.2	4.8	4100	29.4			
839	2015		2015	MALDA		MALDA	NEPZ	NORMAL	NOT_HOSTILE	7.7	2	NW_4018	Triticum_aestivum	47	79	90	116	90	117	35.5	48	1.7	4.4	3750	29.2			
840	2015		2015	MALDA		MALDA	NEPZ	NORMAL	NOT_HOSTILE	7.7	2	KRL_213	Triticum_aestivum	48	79	87	100	86	117	31.8	56	1.4	6.0	4550	32.7			
841	2015		2015	MALDA		MALDA	NEPZ	NORMAL	NOT_HOSTILE	7.7	2	KHARCHIA_65	Triticum_aestivum	49	71	84	160	58	117	40.4	40	1.4	3.4	3400	25.0			
842	2015		2015	MALDA		MALDA	NEPZ	NORMAL	NOT_HOSTILE	7.7	2	RW_3684	Triticum_aestivum	50	77	87	114	82	113	34.4	47	1.7	4.8	3900	30.9			
843	2015		2015	MALDA		MALDA	NEPZ	NORMAL	NOT_HOSTILE	7.7	2	NW_4092	Triticum_aestivum	51	79	89	108	80	117	40.2	47	1.5	4.7	4050	29.3			
844	2015		2015	MALDA		MALDA	NEPZ	NORMAL	NOT_HOSTILE	7.7	2	GW_322	Triticum_aestivum	52	71	87	108	83	113	34.5	53	1.7	5.6	4200	33.6			
845	2015		2015	MALDA		MALDA	NEPZ	NORMAL	NOT_HOSTILE	7.7	2	UP_262	Triticum_aestivum	53	69	81	125	88	113	37.3	45	1.4	5.4	4250	31.5			
846	2015		2015	MALDA		MALDA	NEPZ	NORMAL	NOT_HOSTILE	7.7	2	DBW_51	Triticum_aestivum	54	76	88	116	90	117	38.4	59	1.7	5.0	4200	29.9			
847	2015		2015	MALDA		MALDA	NEPZ	NORMAL	NOT_HOSTILE	7.7	2	RAJ_4238	Triticum_aestivum	55	65	79	99	67	101	43.4	39	1.4	2.4	3050	19.7			
848	2015		2015	MALDA		MALDA	NEPZ	NORMAL	NOT_HOSTILE	7.7	2	DBW_17	Triticum_aestivum	56	79	89	90	79	113	34.1	36	1.3	3.6	2600	34.6			
849	2015		2015	MALDA		MALDA	NEPZ	NORMAL	NOT_HOSTILE	7.7	2	KRL_1-4	Triticum_aestivum	57	62	76	103	69	111	36.6	40	1.7	4.9	3450	35.7			
850	2015		2015	MALDA		MALDA	NEPZ	NORMAL	NOT_HOSTILE	7.7	2	WH_1105	Triticum_aestivum	58	71	84	103	75	112	32.3	58	1.7	4.2	3600	29.3			
851	2015		2015	MALDA		MALDA	NEPZ	NORMAL	NOT_HOSTILE	7.7	2	NW_1067	Triticum_aestivum	59	74	88	98	91	118	35.6	40	1.4	4.2	3600	29.4			
852	2015		2015	MALDA		MALDA	NEPZ	NORMAL	NOT_HOSTILE	7.7	2	DBW_39	Triticum_aestivum	60	74	86	110	88	113	42.4	41	1.7	5.7	4250	33.4			
853	2015		2015	MALDA		MALDA	NEPZ	NORMAL	NOT_HOSTILE	7.7	2	HD_2009	Triticum_aestivum	61	71	83	138	65	109	46.0	50	1.6	4.5	3550	31.5			
854	2015		2015	MALDA		MALDA	NEPZ	NORMAL	NOT_HOSTILE	7.7	2	K_0307	Triticum_aestivum	62	74	84	108	74	113	40.2	37	1.3	4.7	3600	32.5			
855	2015		2015	MALDA		MALDA	NEPZ	NORMAL	NOT_HOSTILE	7.7	2	KRL_3-4	Triticum_aestivum	63	74	87	144	39	113	19.6	30	1.1	1.4	4050	8.9			
856	2015		2015	MALDA		MALDA	NEPZ	NORMAL	NOT_HOSTILE	7.7	2	PDW_314	Triticum_turgidum	64	74	87	97	98	118	43.2	40	1.5	5.2	4500	28.9			
857	2015		2015	MALDA		MALDA	NEPZ	NORMAL	NOT_HOSTILE	7.7	2	KRL_210	Triticum_aestivum	65	71	84	115	97	112	33.6	39	1.3	6.2	5050	30.8			
858	2015		2015	MALDA		MALDA	NEPZ	NORMAL	NOT_HOSTILE	7.7	2	HI_8498	Triticum_turgidum	66	69	84	97	90	111	47.6	48	1.6	4.4	3700	29.6			
859	2015		2015	MALDA		MALDA	NEPZ	NORMAL	NOT_HOSTILE	7.7	2	DBW_46	Triticum_aestivum	67	74	88	122	63	113	43.1	41	1.6	4.0	3400	29.3			
860	2015		2015	MALDA		MALDA	NEPZ	NORMAL	NOT_HOSTILE	7.7	2	RAJ_4229	Triticum_aestivum	68	65	79	99	45	104	33.5	42	1.5	5.6	3850	36.6			
861	2015		2015	MALDA		MALDA	NEPZ	NORMAL	NOT_HOSTILE	7.7	2	CBW_38	Triticum_aestivum	69	76	86	111	87	113	36.1	34	1.4	6.0	4700	31.9			
862	2015		2015	MALDA		MALDA	NEPZ	NORMAL	NOT_HOSTILE	7.7	2	DBW_14	Triticum_aestivum	70	65	78	92	99	111	34.1	41	1.5	5.3	4000	33.3			
863	2015		2015	MALDA		MALDA	NEPZ	NORMAL	NOT_HOSTILE	7.7	2	HD_2932	Triticum_aestivum	71	69	84	107	97	107	32.2	46	1.6	5.9	4250	34.7			
864	2015		2015	MALDA		MALDA	NEPZ	NORMAL	NOT_HOSTILE	7.7	2	DBW_16	Triticum_aestivum	72	81	89	94	90	115	37.8	40	1.5	5.3	3800	34.7			
