# Supplementary material for: Characterising variation in wheat traits under hostile soil conditions in India
Source: PLoS One. 2017 Jun 12;12(6):e0179208. doi: 10.1371/journal.pone.0179208 (PMC5467898; doi:10.1371/journal.pone.0179208)
Supplement: S3 Table — (PDF) [file pone.0179208.s003.pdf]

Supplementary Table 3. Two -way analysis of variance (ANOVA) for assessing the variation in traits due to genotype and site factors between sites in each growing regions .

IIWBR, Karnal and Hisar

Analysis of variance

Variate: GYD

Source of variation	d.f.	s.s.	m.s.	v.r.	F pr.
Genotype	35	251.4918	7.1855	8.69	<.001
Site_Name	1	233.7554	233.7554	282.81	<.001
Genotype.Site_Name	35	64.2658	1.8362	2.22	<.001
Residual	216	178.5362	0.8266		
Total	287	728.0493			

Variate: TGW

Source of variation	d.f.	s.s.	m.s.	v.r.	F pr.
Genotype	35	3964.99	113.29	5.85	<.001
Site_Name	1	350.22	350.22	18.1	<.001
Genotype.Site_Name	35	1390.65	39.73	2.05	0.001
Residual	216	4179.83	19.35		
Total	287	9885.69			

Variate: GWS

Source of variation	d.f.	s.s.	m.s.	v.r.	F pr.
Genotype	35	21.4847	0.6138	4.1	<.001
Site_Name	1	0.999	0.999	6.68	0.01
Genotype.Site_Name	35	3.4093	0.0974	0.65	0.935
Residual	216	32.307	0.1496		
Total	287	58.2001			

Variate: HI

Source of variation	d.f.	s.s.	m.s.	v.r.	F pr.
Genotype	35	5988.87	171.11	9.92	<.001
Site_Name	1	1550.8	1550.8	89.89	<.001
Genotype.Site_Name	35	1867.7	53.36	3.09	<.001
Residual	216	3726.38	17.25		
Total	287	13133.75			

Variate: PHT

Source of variation	d.f.	s.s.	m.s.	v.r.	F pr.
Genotype	35	26853.38	767.24	13.97	<.001
Site_Name	1	3.74	3.74	0.07	0.795
Genotype.Site_Name	35	865.48	24.73	0.45	0.997
Residual	216	11864.83	54.93		
Total	287	39587.43			

Variate: DTM

Source of variation	d.f.	s.s.	m.s.	v.r.	F pr.
Genotype	35	1172.25	33.493	5.89	<.001
Site_Name	1	4125.347	4125.347	725.93	<.001
Genotype.Site_Name	35	408.403	11.669	2.05	0.001
Residual	216	1227.5	5.683		
Total	287	6933.5			

Variate: DTA

Source of variation	d.f.	s.s.	m.s.	v.r.	F pr.
Genotype	35	2687.486	76.785	9.1	<.001
Site_Name	1	2346.125	2346.125	278.06	<.001
Genotype.Site_Name	35	195.875	5.596	0.66	0.926
Residual	216	1822.5	8.438		
Total	287	7051.986			

Kumarganj-reclaimed and Kumarganj-sodic

Analysis of variance

Variate: GYD

Source of variation	d.f.	s.s.	m.s.	v.r.	F pr.
Genotype	35	16.7878	0.4797	0.91	0.611
Site_Name	1	113.7334	113.7334	216.75	<.001
Genotype.Site_Name	35	14.8553	0.4244	0.81	0.77
Residual	216	113.3421	0.5247		
Total	287	258.7186			

Variate: TGW

Source of variation	d.f.	s.s.	m.s.	v.r.	F pr.
Genotype	35	1925.19	55.01	4.28	<.001
Site_Name	1	1854.91	1854.91	144.39	<.001
Genotype.Site_Name	35	1164.91	33.28	2.59	<.001
Residual	216	2774.78	12.85		
Total	287	7719.8			

Variate: GWS

Source of variation	d.f.	s.s.	m.s.	v.r.	F pr.
Genotype	35	8.19862	0.23425	2.8	<.001
Site_Name	1	9.0185	9.0185	107.93	<.001
Genotype.Site_Name	35	6.21747	0.17764	2.13	<.001
Residual	216	18.048	0.08356		
Total	287	41.4826			

Variate: HI

Source of variation	d.f.	s.s.	m.s.	v.r.	F pr.
Genotype	35	1683.32	48.09	1.68	0.014
Site_Name	1	106	106	3.7	0.056
Genotype.Site_Name	35	917.31	26.21	0.91	0.611
Residual	216	6195.34	28.68		
Total	287	8901.97			

Variate: PHT

Source of variation	d.f.	s.s.	m.s.	v.r.	F pr.
Genotype	35	16944.95	484.14	8.68	<.001
Site_Name	1	10821.66	10821.66	194.02	<.001
Genotype.Site_Name	35	2724.37	77.84	1.4	0.08
Residual	216	12047.52	55.78		
Total	287	42538.5			

Variate: DTM

Source of variation	d.f.	s.s.	m.s.	v.r.	F pr.
Genotype	35	543.29	15.52	0.37	1
Site_Name	1	899.59	899.59	21.26	<.001
Genotype.Site_Name	35	110.29	3.15	0.07	1
Residual	216	9139.75	42.31		
Total	287	10692.91			

Variate: DTA

Source of variation	d.f.	s.s.	m.s.	v.r.	F pr.
Genotype	35	2177.54	62.22	1.35	0.103
Site_Name	1	65.17	65.17	1.41	0.236
Genotype.Site_Name	35	197.2	5.63	0.12	1
Residual	216	9954.25	46.08		
Total	287	12394.16			

Malda and Pundibari sites

Analysis of variance

Variate: GYD

Source of variation	d.f.	s.s.	m.s.	v.r.	F pr.
Genotype	35	74.5377	2.1296	2.43	<.001
Site_Name	1	1.4358	1.4358	1.64	0.202
Genotype.Site_Name	35	23.1255	0.6607	0.75	0.839
Residual	216	189.1562	0.8757		
Total	287	288.2552			

Variate: TGW

Source of variation	d.f.	s.s.	m.s.	v.r.	F pr.
Genotype	35	2705.16	77.29	4.43	<.001
Site_Name	1	840.71	840.71	48.21	<.001
Genotype.Site_Name	35	607.96	17.37	1	0.481
Residual	216	3767.09	17.44		
Total	287	7920.93			

Variate: GWS

Source of variation	d.f.	s.s.	m.s.	v.r.	F pr.
Genotype	35	12.0278	0.3437	2.28	<.001
Site_Name	1	5.5288	5.5288	36.74	<.001
Genotype.Site_Name	35	2.3313	0.0666	0.44	0.997
Residual	216	32.5063	0.1505		
Total	287	52.3942			

Variate: HI

Source of variation	d.f.	s.s.	m.s.	v.r.	F pr.
Genotype	35	4818.29	137.67	1.62	0.02
Site_Name	1	404.32	404.32	4.77	0.03
Genotype.Site_Name	35	1303.21	37.23	0.44	0.998
Residual	216	18308.82	84.76		
Total	287	24834.63			

Variate: PHT

Source of variation	d.f.	s.s.	m.s.	v.r.	F pr.
Genotype	35	37420.5	1069.2	6.68	<.001
Site_Name	1	4602.8	4602.8	28.78	<.001
Genotype.Site_Name	35	8017.3	229.1	1.43	0.065
Residual	216	34549	159.9		
Total	287	84589.6			

Variate: DTM

Source of variation	d.f.	s.s.	m.s.	v.r.	F pr.
Genotype	35	3433.4	98.1	1.83	0.005
Site_Name	1	3200	3200	59.6	<.001
Genotype.Site_Name	35	755.25	21.58	0.4	0.999
Residual	216	11598	53.69		
Total	287	18986.65			

Variate: DTA

Source of variation	d.f.	s.s.	m.s.	v.r.	F pr.
Genotype	35	6269.45	179.13	9.42	<.001
Site_Name	1	437.59	437.59	23.01	<.001
Genotype.Site_Name	35	816.29	23.32	1.23	0.192
Residual	216	4107.75	19.02		
Total	287	11631.08			

Abbreviations	Description
**GYD**	Grain yield t/ha
**TGW**	1000 grain weight,g
**GWS**	Grain weight per spike, g
**HI**	Harvest index, %
**PHT**	Plant height at maturity, cm
**DTM**	Days to maturity, days
**DTA**	Days to anthesis, days
